# Proteome-scale autoantibody profiling in PSC: Associations with clinical phenotypes and evidence for neuroendocrine deregulations

**DOI:** 10.1016/j.jhepr.2025.101719

**Published:** 2025-12-23

**Authors:** Martin Cornillet, Aiva Lundberg Båve, Dan Sun, Ghada Nouairia, Christina Villard, Aristeidis Grigoriadis, Erik von Seth, Hannes Jansson, María Bueno Álvez, Sofia Bergström, Peter Nilsson, Mathias Uhlén, Fredrik Edfors, Per Stål, SweHep consortium, Mårten Werner, Nils Nyhlin, Fredrik Rorsman, Johan Vessby, Nilsson Emma, Antonio Molinaro, Annika Bergquist, Stergios Kechagias, Maria Antonella Burza, Lina Lindström, Ernesto Sparrelid, Niklas K. Björkström, Jonas Halfvarson, Annika Bergquist

**Affiliations:** 1 Department of Medicine Huddinge, Karolinska Institute, Stockholm, Sweden; 2 Department of Upper Abdominal Diseases, Karolinska University Hospital, Stockholm, Sweden; 3 Department of Transplantation Surgery, Karolinska University Hospital, Stockholm, Sweden; 4 Department of Radiology, Karolinska University Hospital, Stockholm, Sweden; 5 Division of Surgery and Oncology, Department of Clinical Science, Intervention and Technology, Karolinska Institute, Karolinska University Hospital, Stockholm, Sweden; 6 Department of Protein Science, SciLifeLab, KTH Royal Institute of Technology, Stockholm, Sweden; 7 Department of Gastroenterology, Faculty of Medicine and Health, Orebro University, Orebro Sweden

**Keywords:** primary sclerosing cholangitis, autoantibody, liver transplantation, SUPRIM cohort, neuro-endocrine, cryptic antigen, epitope drifting

## Abstract

**Background & Aims:**

Primary sclerosing cholangitis (PSC) is a rare cholestatic liver disease with heterogeneous phenotypes and progression. Autoimmune traits, such as the presence of autoantibodies, are suspected to drive its heterogeneity.

**Methods:**

We performed a proteome-scale autoantibody screen of IgG and IgA isotypes using >42,100 protein fragments. This was followed by a validation of 1,153 selected autoantibodies, in serum samples from 466 patients with PSC in a longitudinal setting using the SUPRIM cohort and 214 controls.

**Results:**

We identified autoantibodies associated with clinical phenotypes, biochemical and clinical severity, comorbidities, and disease progression (*e.g.* alkaline phosphatase and albumin level *p <*e-10, presence of hepatobiliary malignancies *p <*0.001, seroconversion before transplantation *p <*0.001). Rather than a single universal autoantibody marker, small patient subgroups were positive for various autoantibodies with variable specificity. Global analysis of autoantigen targets revealed an overrepresentation of proteins normally expressed in immune-privileged sites, including the brain, testis, and retina. When interrogating tissue-specific autoantigen co-expression linked to expression and splicing quantitative trait loci of PSC risk variants, the thyroid emerged as an additional relevant tissue. We also detected increased autoantibody diversity associated with PSC duration and end-stage disease, already observable several years before liver transplantation. Multiomics analysis across body compartments confirmed neuroendocrine dysregulation in PSC. Our results are provided as a resource for further studies.

**Conclusions:**

Overall, our data support the cryptic antigen and epitope-drifting autoimmune theories and indicate that neuroendocrine dysregulation may contribute to PSC pathogenesis.

**Impact and implications:**

From a proteome-scale profiling of the SUPRIM cohort, we provide a short list of autoantibodies associated with clinical phenotypes and progression, along with the peptide sequences used to capture them. We identify across multiple datasets neuroendocrine deregulations in primary sclerosing cholangitis and provide a short list of related key plasma proteins. These data and technical details should facilitate validation studies, investigations of related pathophysiological mechanisms and development of low-cost tools for diagnostic or prognostic purposes.

## Highlights

• Proteome-scale profiling in the SUPRIM cohort identifies autoantibodies linked to clinical phenotypes, progression, and comorbidities in PSC.
• Autoantigen targets are enriched in immune-privileged tissues, with thyroid, brain, testis, and retina implicated.
• Analysis of the autoantibody targets, quantitative trait loci of PSC risk variants, and plasma proteomics provide evidence for neuroendocrine deregulations in PSC"

## Graphical abstract

**Figure:**
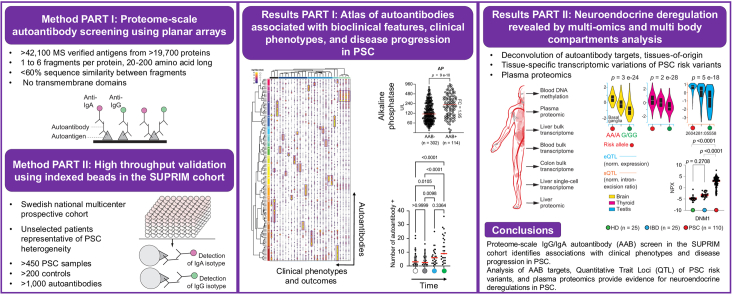

## Introduction

Primary sclerosing cholangitis (PSC) is a rare cholestatic liver disease charactized by multifocal biliary strictures and a variable degree of fibrosis, progressing over several decades. It is mostly studied and managed as a single disease entity although the clinical presentation and progression are highly heterogeneous.[1], [2], [3], [4], [5] PSC can affect both sexes but is predominantly diagnosed in males in their thirties, although the age at diagnosis spans from childhood^6^^,^^7^ to late adulthood.^8^ A large proportion of patients also have inflammatory bowel disease (IBD) with specific characteristics.^9^ As the disease progresses, many patients require liver transplantation, with recurrence in the graft frequently observed.^10^ Although the median time from diagnosis to liver transplantation is around 20 years, some patients rapidly progress and undergo transplantation during the first years after diagnosis.^11^ Moreover, a wide spectrum of additional clinical complications, such as bacterial cholangitis, liver decompensation and hepatobiliary (HB) cancers, mostly cholangiocarcinoma (CCA) may occur. Additional factors contributing to clinical heterogeneity include the involvement of small or large bile ducts, cholangiographic appearance,^12^ features of autoimmune hepatitis, circulating IgG4 levels^13^ and liver function test results.^12^^,^[14], [15], [16], [17] These observations strongly suggest that PSC represents a patchwork of multiple, smaller disease entities. To date, no biological explanation exists for this complex heterogeneity, limiting basic research, clinical management, and therapeutic development.

Although the etiology remains unknown, PSC displays several autoimmune features. Genome-wide association studies have identified the human leukocyte antigen (HLA) complex as the strongest genetic risk factor, with additional associations linked to genes involved in immune-mediated diseases.^18^ In addition, the presence of autoantibodies in patients with PSC has historically been reported despite poor specificity, sensitivity, and clinical significance. Among them are anti-smooth muscle, anti-nuclear, and anti-neutrophil cytoplasmic autoantibodies.[19], [20], [21] Besides IBD and autoimmune hepatitis, the presence of other concomitant autoimmune diseases is common in patients with PSC[22], [23], [24] and immunomodulatory drugs might be effective in some patient subsets.[25], [26], [27], [28] However, although an autoimmune etiology of PSC was postulated in the eighties,^29^ the identification of autoreactive T or B cells and the mechanisms underlying central or peripheral immune tolerance defects have not been demonstrated.

Taken together, the autoimmune features and clinical heterogeneity of PSC have fueled the search for pathogenic autoantibodies as a “holy grail”.^30^ Clinically, such autoantibodies could provide diagnostic and prognostic tools, and mechanistically, they could reveal autoreactive lymphocytes and immune tolerance defects. Therapeutically, they could allow patient stratification for personalized interventions, such as antigen-specific B- or T-cell depletion.[31], [32], [33] Indeed, in other autoimmune diseases – such as rheumatoid arthritis, pemphigus, myasthenia gravis, and myositis – pathogenic autoantibodies define clinical subtypes and guide therapeutic options.[34], [35], [36] However, challenges in studying PSC have limited large-scale, matched clinical and autoantibody analyses. These challenges include the low prevalence of PSC, complex clinical phenotypes, and the need for standardized, longitudinal clinical assessments. Previous studies have typically analyzed small cohorts and a limited number of antigens.^30^

To tackle these challenges, we combined proteome-scale autoantibody profiling^37^ with a national multicenter prospective study,^38^ providing an Atlas of autoantibody profiles associated with clinical phenotypes and progression in PSC.

## Materials and methods

### Planar arrays

Whole proteome protein fragment arrays were generated as previously described.^37^ Briefly, each array consists of 58,000 spots, representing 42,000 unique protein fragments (two glass slides x 21k array), which covers approximately 94% of the human proteome from a gene centric point of view.^39^^,^^40^ The antigens were produced within the Human Protein Atlas project^41^ (www.proteinatlas.org) and consist of protein fragments of approximately 20-200 aa selected based on sequence uniqueness within the human proteome (expressed in *Escherichia coli* with a six histidine and albumin binding protein tag, His6ABP). The samples (sera pools) were diluted 1:12.5 in assay buffer, incubated in assay buffer for 15 min at room temperature, then transferred to the slides containing the antigen arrays to incubate for 1 h at room temperature on the bench without shaking. For autoantibody quantification, anti-human IgA Alexa 647 (α chain specific, Jackson, 109-605-011, 1.4 mg/ml, diluted 1:15,000) and goat anti-human IgG Alexa 647 (H+L, Life Technology, #A21445, 2 mg/ml, diluted 1:15,000) were sequentially incubated in the dark for 1 h at room temperature on a shake table. The slides were scanned using a CapitalBio LuxScan HT24 instrument following each incubation. The values from the IgA scan were subtracted from the resulting values from the IgG scan, to estimate reactivities unique for IgG. A comparison to an internal database was also made per immunoglobulin type (IgA: three pools. IgG: 22 individual samples and 20 pools) to give an indication if reactive antigens were selectively reactive in this study or more generally reactive.

### Bead arrays

Three suspension bead array assays were prepared separately, one for IgA and two for IgG. The antigens and technical controls were covalently coupled to color-coded magnetic beads (MagPlex, Luminex Corp., Austin, TX) using NHS- and EDC-based chemistry. The samples were diluted 1:250 in assay buffer. The diluted samples were mixed with the suspension bead array and incubated for 2 h. Any antibody binding to the beads was fixed by incubating the beads in 0.2% paraformaldehyde for 10 min. For the assays measuring IgG, Goat Anti-Human IgG Fc Secondary Antibody PE (12-4998-82, eBioscience™, Invitrogen) and, for the assay measuring IgA, Goat Anti-Human IgA Antibody DyLight 550 Conjugated (A80-102D3, Bethyl Laboratories) were applied for 30 min to enable readout using a FlexMap 3D instrument (Luminex Corp., Austin, TX).

### Patients

In the first phase (planar array), 33 patients with PSC were included as described in the results section and supplementary material. Deviation from the selection criteria was noticed retrospectively as one patient from the “progressor” group had received adalimumab before sampling. The rationale for the selection and grouping of patients with PSC was based on the hypothesis that an unknown autoantibody could drive PSC phenotypes. Groups were designed to mainly differ by one single feature that could be explained by the presence of an autoantibody, such as the presence of IBD or of advanced stage of the disease. Furthermore, matching at the group level was performed for several criteria that could potentially induce bias (such as total IgG and IgA levels, sex, age). In each group, we aimed to balance representativeness and heterogeneity. For example, various types of IBD were included to reflect the clinical setting, and both advanced and early disease stages were included within the non-IBD or the CCA groups. The group sizes were chosen to balance having too few individuals, which risks poor representativeness and missing low-prevalence autoantibodies, and too many individuals, which risks diluting low-prevalence autoantibodies below the detection limit. We estimated that groups of six to eight individuals would provide reasonably good representation of each phenotype while allowing detection of autoantibodies with a prevalence as low as 12.5% (if at least one of eight patients had the autoantibody). A total of 33 patients were included on five arrays which we considered as a reasonable number of individuals and replicates to identify autoantibodies in PSC. In the second phase (bead-based approach), we used sera from 419 individuals with PSC from the SUPRIM cohort,^42^ collected prospectively with yearly data on biochemistry, clinical parameters, MRI, interventions, and outcomes. This national multicenter prospective collection of patients with PSC represents the heterogeneity of PSC phenotypes and progression.^38^ Sera were analyzed at baseline for most individuals (n = 405) and at the timepoint closest to any severe event (liver transplantation, HB cancer, or death) in 47. Small duct PSC was diagnosed in cases with clinical findings of PSC (cholestasis without any other cause), presence of IBD, and a biopsy with typical findings of PSC. An autoimmune hepatitis (AIH) overlap diagnosis was assigned in cases with typical features of AIH, including elevated transaminases, increased total IgG, and a liver biopsy showing interface hepatitis, in addition to PSC cholangiographic changes.^5^ A diagnosis of cirrhosis was assigned in cases with clinical or radiological signs of cirrhosis, evidence of portal hypertension, and/or an elastography measurement >20 kPa or a biopsy confirming F4 fibrosis. Patients with PSC were considered as having active IBD when requiring intensified treatment (increased doses of 5-ASA, a course of steroids or introduction of azathioprine or biological treatment). Sera from healthy donors (n = 91), individuals with other liver diseases (n = 62) and individuals with IBD (n = 62) served as control samples. IBD controls were matched at a group level for age at IBD diagnosis, subtype of IBD and colectomy. Healthy donor controls were matched for sex and age at time of sampling. Liver disease controls comprised individuals with AIH (n = 15), primary biliary cholangitis (PBC) (n = 13), cirrhosis (alcoholic, metabolic or cryptogenic) (n = 14), non-PSC-CCA (n = 9), and non-PSC-cholangiopathy in need of endoscopic retrograde cholangiopancreatography (n = 10). Hepatitis B and C were exclusion criteria in the first phase of the study as well as in the second phase for the controls. Although this was not an exclusion criterion for patients with PSC in the second phase, only one patient was positive for HCV RNA by PCR at inclusion (2014), but was subsequently treated and considered cured in 2016. For the proteins in plasma (Olink), we included samples from 110 patients with PSC representative of the disease spectrum. Ethical approval was acquired from the Regional Ethical Board, Stockholm and Uppsala, Sweden (Dnr 06/245-1, 2011/2-6, 2012/2141-31/1, 2013/188-31/1, 2013/2084-31/1, 2021-05560-02).

### Statistical analysis

To detect associations between autoantibodies and PSC features, we analyzed whether the presence or absence of autoantibodies was associated with specific parameters. In these two groups (AAB-pos *vs.* AAB-neg), the proportions of binary parameters (such as male/female) were analyzed by Chi-squared test (R Studio 2022.02.3, function chisq.test). The levels of continuous parameters (such as liver enzymes) were analyzed using the non-parametric Mann-Whitney test (R Studio 2022.02.3, function wilcox.test) to avoid influence of outliers. To detect associations between autoantibodies and severe events, we compared the samples taken close to the events with samples taken at inclusion from patients who did not develop any event during follow-up. Some patients were reported with multiple events such as both “transplanted” and “death”, or “HB cancer” and “death”. In those cases, we assigned these patients to the single event that occurred first. “HB cancer” comprised CCA, gallbladder cancer, hepatocellular carcinoma, and “death” comprised various causes related to non-HB cancers (such as breast or colorectal) or others (such as COVID-19 or stroke). No data was imputed. The numbers of patients used for each analysis are available in Table S3. Details on data handling, including normalized steps, quality controls are provided in the supplementary materials and methods document. Within the highlighted clusters in Fig. 2, the top significant results were selected with a maximum of six AABs to illustrate the relationship with biological, radiological and clinical parameters in Fig. 3. The function ggballoonplot from ggpubr package was used for Fig. 2. The clustering was performed using the Ward’s method (function hclust, method ward.D2). ROC performances were computed based on logistic regression (Prisms 9). Analysis of interactions was not included in the models. ROC curves were mainly used to depict associations, not to establish predictive models to be used in a real-life clinical setting. Statistics for splicing and expression quantitative trait loci (sQTLs and eQTLs) were extracted from GTEx; co-expression analysis was performed using AnalyseR; chord and Sankey diagrams were generated with RAWGraphs 2.0; and odds ratios were computed using MedCalc. For the odds ratios, standard errors and 95% CIs were calculated according to Altman, 1991.^43^ *P* values were calculated according to Sheskin *et al.*, 2004 (p. 542).^44^ For autoimmune disease (AID), *p* values were selected based on the most significant associations with thyroid and skin-related AID (the most common in our cohort). For hepatic decompensation, *p* values were selected based on the most significant associations with either ascites, variceal bleeding, or encephalopathy at the time of sampling. Statistics for each of these parameters are provided individually in the supplementary material. Enrichment patterns were defined according to the Human Protein Atlas classification as follows: tissue enriched, at least four-fold higher mRNA levels in a particular tissue compared with any other tissue; group enriched, at least four-fold higher average mRNA levels in a group of 2–5 tissues compared with any other tissue; tissue enhanced, at least four-fold higher mRNA levels in a particular tissue compared with the average level in all other tissues; cell type-enriched genes, at least four-fold higher expression levels in one cell type compared with any other analyzed cell type; cell type group-enriched genes, enriched expression in a small number of cell types (2–10); and cell type-enhanced genes, genes with moderately elevated expression.

**Fig. 1:**
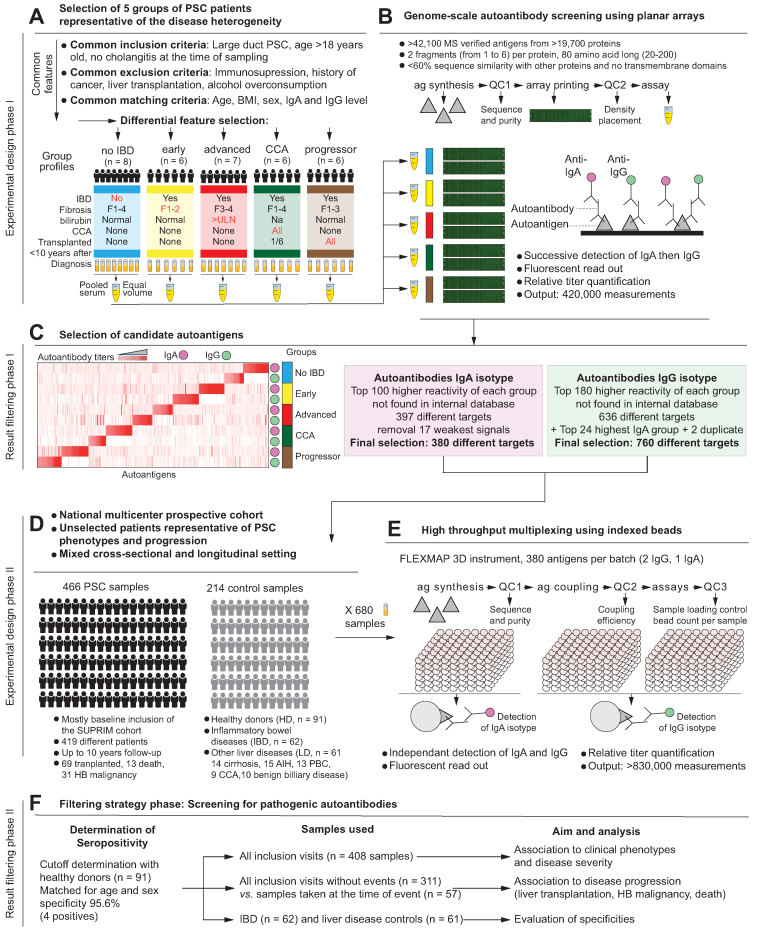
Study design, analytical pipeline, and workflow. (A) Selection of the samples used for the proteome-scale autoantibody profiling. Serum from representative groups of patients with PSC were pooled before the assay. (B) Workflow and output of the planar arrays. Autoantibodies from IgA the IgG isotypes were quantified after passive immunocapture on the printed arrays. (C) Results and selection of candidate autoantigens from phase I. Autoantibody titers for each group are depicted on the left side and strategy of selection for phase II on the right side. (D) Overview of the samples used for phase II of the study. Serum samples from prospectively collected patients with PSC, as well as from controls, were included. (E) Workflow and output of the bead-based assays. Autoantibodies from IgA the IgG isotypes were quantified after passive immunocapture on the antigen-coupled beads. (F) Strategy of analysis for phase II. Cut-off for antibody positivity was determined at high specificity with healthy donors and associated with an array of parameters of PSC severity and progression. ag, antigen; AIH, autoimmune hepatitis; CCA, cholangiocarcinoma; HB, hepatobiliary; IBD, inflammatory bowel disease; MS, mass spectrometry; PBC, primary biliary cholangitis; PSC, primary sclerosing cholangitis; QC, quality control.

**Fig. 2:**
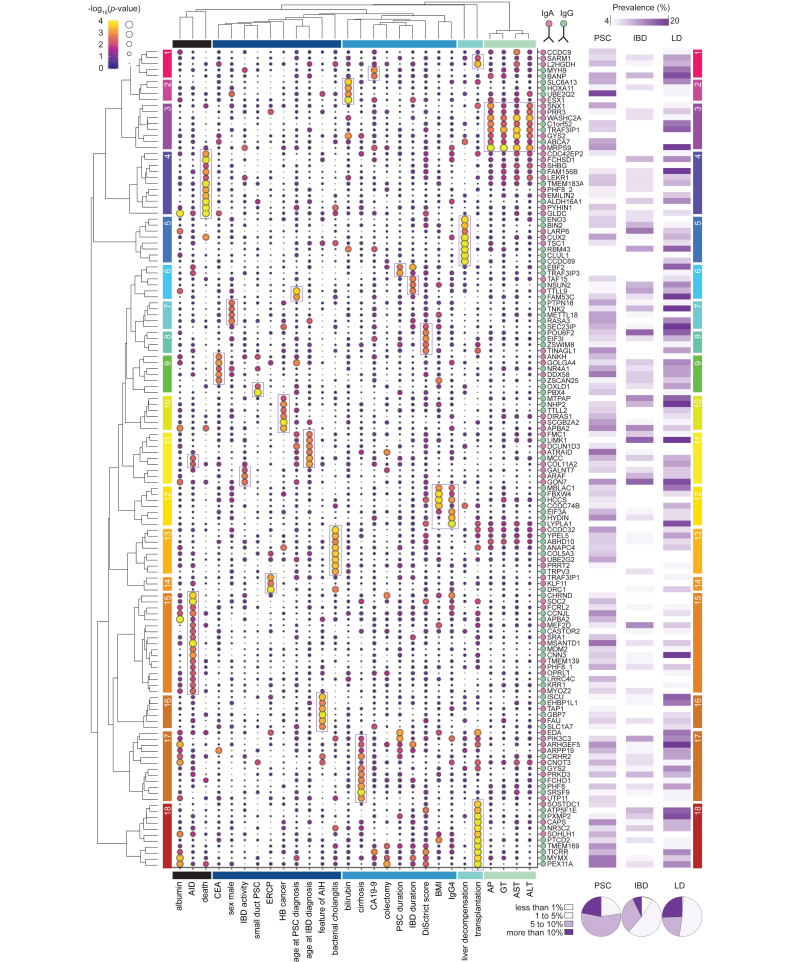
Autoantibody associations with clinical features, comorbidities, severity, and progression in PSC: An overview. Results from phase II are summarized by bubbles representing the significance level of associations between autoantibodies and PSC features. Autoantigens and autoantibody isotypes are indicated on the right side respectively with abbreviations of protein names, green and purple filled circles. Clusters of autoantibodies associated with a specific feature are numbered on the left side within colored rectangles and further visualized using boxes inside the bubble plot. Prevalence of autoantibodies in PSC and controls are indicated using shades of purple on the right side of the bubble plot and corresponding summary results are indicated in pie charts below it. In summary, each large yellow/orange bubble shows an association between the presence of an autoantibody (on the right) and a PSC feature (at the bottom). Chi-square and Mann-Whitney tests were used to detect associations. The size of the groups might differ and are given in Table S3. AID, autoimmune disease; AIH, autoimmune hepatitis; ALT, alanine aminotransferase; AP, alkaline phosphatase; AST, aspartate aminotransferase; CEA, carcinoembryonic antigen; CA-19-9, carbohydrate antigen 19-9; ERCP, endoscopic retrograde cholangiopancreatography; GT, gamma-glutamyltransferase; HB, hepatobiliary; IBD, inflammatory bowel disease; PSC, primary sclerosing cholangitis.

**Fig. 3:**
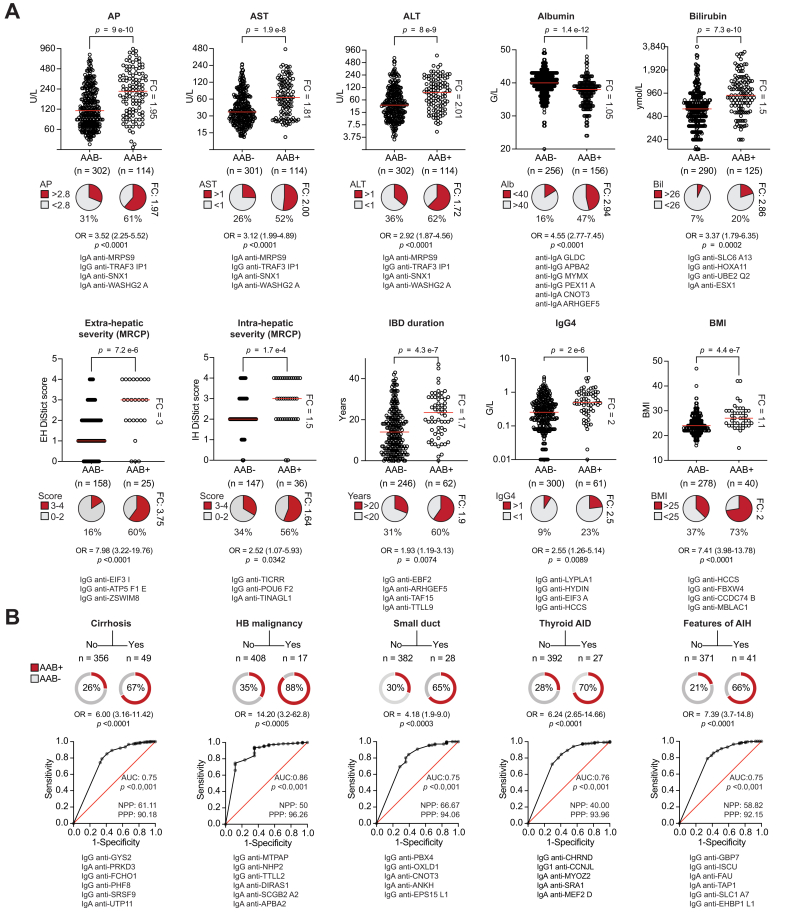
Autoantibody associations with clinical features, comorbidities, severity, and progression in PSC: Representative findings. Association of autoantibodies with continuous/discrete (A) and binary (B) parameters. (A) Median levels are depicted as well as the corresponding FC in patients found autoantibody positive and negative (AAB+/-) at baseline (AAB+ are patients positive for at least one of the autoantibodies listed below the graphs). The proportions of patients above or below indicated thresholds are illustrated in pie charts with the corresponding FC on the side and ORs below them. For the dot plots, Mann-Whitney tests were used. (B) Number of patients presenting the specific features are indicated, as well as the proportion of those autoantibody positive and negative (AAB+/-, pie charts) at baseline (AAB+ are patients positive for at least one of the autoantibodies listed below the graphs). HB malignancy compared samples of patients without and with HB malignancy diagnosed within 5 years after baseline inclusion of the prospective study (respectively samples at baseline or close to diagnosis). The corresponding receiver-operating characteristic curves are depicted below with AUC and NPP/PPP. ROC curves were mainly used to depict associations, not to establish predictive models. AAB, autoantibody; AID, autoimmune disease; AIH, autoimmune hepatitis; ALT, alanine aminotransferase; AP, alkaline phosphatase; AST, aspartate aminotransferase; FC, fold-change; HB, hepatobiliary; IBD, inflammatory bowel disease; MRCP, magnetic resonance cholangiopancreatography; NPP, negative predictive power; OR, odds ratio; PPP, positive predictive power; PSC, primary sclerosing cholangitis.

## Results

### Proteome-scale screening and longitudinal prospective sampling for identification of pathogenic autoantibodies in PSC

In the search for potential pathogenic autoantibodies in PSC, we developed a two-phase approach. In the first phase, we screened for autoantibodies associated with common PSC features representing both clinical challenges and biological knowledge gaps. We established five carefully selected groups of patients with PSC based on common inclusion and exclusion criteria, matching criteria, and distinguishing features (Fig. 1A), four of which included patients with IBD. The groups represented early and late stages of the disease (“early” or “advanced” with low or high fibrosis and bilirubin), cholangiocarcinoma (“CCA”), and relatively fast progressors toward liver transplantation (“progressor”, with normal bilirubin level at the time of sampling). In addition, a group representing patients with PSC without IBD (“no IBD”, with various fibrosis stages) was included. More detailed profiles of these groups are provided in Fig. S1. Sequential quantification of autoantibodies of IgA and IgG isotypes was performed towards the human proteome using custom-made planar arrays (Fig. 1B, Table S1). The top highest reactivities against autoantigens showed a non-overlapping pattern between the five groups both for IgA and IgG suggesting the presence of multiple autoantibodies in PSC rather than a single autoantibody hallmark of the disease (Fig. 1C, Table S1). Finally, 760 and 380 autoantigens targeted by IgG and IgA autoantibodies, respectively, were selected based on their highest titers to explore associations with disease severity.

In the second phase, we quantified the candidate autoantibodies in 466 serum samples from the SUPRIM cohort,^42^ and 214 control serum samples (healthy individuals, patients with IBD and patients with other liver diseases, Fig. 1D, Table 1). From the SUPRIM cohort, 419 patients with PSC were tested at the time of inclusion in the multicenter national prospective study. In 47 of these, a second sample taken closest to the end point events was assayed to enable a time-dependent analysis (28 transplants, five deaths and 14 HB malignancies). During follow-up (up to 10 years), 69 transplanted patients and 31 diagnosed with HB malignancy were included in the overall experiment. All the 380 IgA and 760 IgG autoantibodies were quantified in the 680 PSC and control samples using a bead-based approach (Fig. 1E, Table S2). As a rationale for data analysis, we expected pathogenic autoantibodies to be relatively rare in the general population and to associate with disease severity and/or progression. However, antibodies can be unspecific if present in other diseases, possibly if a common cause would trigger autoantibody generation without perpetuation of the autoantigenic target. Following this reasoning, we determined cut-offs for autoantibody positivity using healthy donors, and we analyzed their associations with PSC features (Fig. 1F, Table S3). Disease specificity was evaluated using patients with IBD and patients with other liver diseases.

**Table 1:** Clinical characteristics of patients with PSC, healthy donors, and diseased controls.

	PSC (n = 419)	Healthy donor (n = 91)	IBD controls (n = 62)	Liver disease control (n = 61)
Age (median, IQR)	39, 32-51	38, 27-54	46, 31-56	62, 45-73
Sex male (n, %, n, missing)	282, 68%,1	59, 65%, 0	49, 79%, 0	25, 41%, 0
BMI (median, IQR)	24, 22-27	-	-	-
PSC features				
Years with PSC (median, IQR)	8, 4-15	-	-	-
Age at PSC onset (median, IQR)	31, 21-40	-	-	-
Cirrhosis (n, %, n missing)	49, 12%, 14	0	0	22, 36%, 0
Cholangitis antibiotic (n, %, n missing)	21, 5%, 14	-	-	-
Jaundice intervention (n, %, n missing)	16, 4%, 14	-	-	-
Overlap AIH (n, %, n missing)	41,10%,14	-	-	-
IgG4 phenotype (n, %, n missing)	6, 2%, 58	-	-	-
Small duct PSC (n, %, n missing)	28, 7%, 9	-	-	-
Encephalopathy (n, %, n missing)	4, 1%, 4	-	-	-
Variceal bleeding (n, %, n missing)	6, 2%, 5	-	-	-
Ascites (n, %, n missing)	2, 0.5%, 4	-	-	-
IBD features				
IBD (n, %, n missing)	311, 74%, 2	0	62, 100%, 0	0
Age at IBD diagnosis (median IQR)	21, 15-32	-	24, 17-33	-
Years with IBD (median IQR)	16, 8-25	-	11, 5-30	-
Colectomy (n, %, n missing)	67, 16%, 84	-	10, 16%, 0	-
Active IBD (baseline) (n, %, n missing)	122, 29%, 90	-	NA	-
Biochemistry, (median (IQR)				
AST (U/L)	39 (26-73)	-	-	36 (24-50)
ALT (U/L)	51 (28-108)	-	-	29 (21-48)
AP (U/L)	126 (78-258)	-	-	84 (66-126)
GT (U/L)	126 (41-378)	-	-	60 (29-168)
Bilirubin (μmol/L)	11 (8-16)	-	-	10 (8-17)
Albumin (g/L)	39 (36-42)	-	-	36 (33-39)
CEA (μg/L)	1.4 (1-2.3)	-	-	-
CA199 (U/ml)	9.8 (5-20)	-	-	-
Autoimmune features (n, %, n missing)				
AID	75, 18%	0	2, 3%, 8	11, 18%, 41
Thyroid	27, 6%	-	-	4, 7%
Skin	26, 6%	-	1, 2%	4, 7%
Other AID	46, 11%	-	-	3, 5%
Hard endpoints (n, %)				
Transplantation	68, 16%	-	-	-
Hepatobiliary cancer	31, 7%	-	-	9, 15%
Death	14, 3%	-	-	-

AID, autoimmune disease; AIH, autoimmune hepatitis; ALT, alanine aminotransferase; AP, alkaline phosphatase; AST, aspartate aminotransferase; GT, gamma-glutamyl transferase; IBD, inflammatory bowel disease; PSC, primary sclerosing cholangitis.

In summary, we screened for both IgG and mucosal-relevant IgA autoantibodies by combining the world’s largest human protein array with a bead-based approach, without any *a priori* assumptions regarding the targeted organs or cell types. We coupled this approach with a cross-sectional and longitudinal assessment using one of the largest prospective biological collections in PSC to unravel associations with disease features and progression. Compiled results are provided as a resource for future studies.

### Atlas of autoantibodies associated with bioclinical features and disease progression in PSC

Following the strategy described above, we identified an array of autoantibodies significantly associated with clinical features, disease severity, and progression in PSC. The main results are summarized in Fig. 2 with some representative findings provided in Fig. 3. The unbiased selection of the top significant associations for each feature revealed a global independent pattern where most of the autoantibodies were associated with one single feature. Both IgG and IgA isotypes were found. Biochemical markers of liver damage measured by alkaline phosphatase, aspartate aminotransferase, and alanine aminotransferase were associated with cluster 3 (group of autoantibodies) and could be distinguished from measures of liver function such as albumin and bilirubin (cluster 2) (Fig. 2). Patients with autoantibodies had a two-to-three-fold increase in biochemical markers of liver damage (Fig. 3A). Regarding the clinical subtypes of PSC, small duct PSC was associated with PBX4 in cluster 9 and overlap AIH PSC with cluster 16, whereas a high level of serum IgG4 was associated with cluster 12 (Fig. 2). The association between autoantibody profiles and these PSC subtypes yielded odds ratios of 2.55 to 7.39 (Fig. 3A,B, Table S4). Autoantibodies associated with cirrhosis (cluster 17) were distinct from those associated with liver transplantation (cluster 18), and their profile discriminated patients with or without cirrhosis with a positive predictive value of 90.2% and an odds ratio of 6.0 (Fig. 3B). Representing an array of severe complications, liver decompensation, bacterial cholangitis, and jaundice intervention (endoscopic retrograde cholangiopancreatography) were associated with clusters 5, 13 and 14, respectively. Furthermore, each of the disease progression events assessed by death, liver transplantation and HB malignancy were associated with distinct clusters (4, 18,10) (Fig. 2). The latter was independent of clusters 9 and 1, which were associated with tumor markers CEA and CA19-9. Autoantibody profiles associated with HB cancer with a positive predictive value of 96.3% and an odds ratio of 14.20 (Fig. 3B). Both age of onset and disease duration of both PSC and IBD were associated with specific clusters. In addition, sex, and the presence of other AID, associated with cluster 7 and 15. Surprisingly, BMI was associated with the specific cluster 12 together with IgG4 (Fig. 2), with a two-fold increase of overweight patients (BMI >25, 37% *vs*. 73%, Fig. 3A). Finally, disease severity assessed by the MRI-based DiStrict score^12^ was associated with cluster 8 (Fig. 2) and distinct autoantibody profiles were associated with intra- or extrahepatic biliary strictures with upstream dilatation (Fig. 3A). Most autoantibodies showed a prevalence of between 5 and 10% in PSC and less than 5% in IBD and liver disease controls (Fig. 2 right panel, Table S5). However, their prevalence varied substantially, and some autoantibodies were more common in controls. Overall, these results did not confirm the hypothesis of one antibody marker of the disease but rather mirrored the very large heterogeneity of PSC features and disease course seen in the clinic.

In short, our analysis revealed small subgroups of patients with PSC being seropositive for several autoantibodies of IgA and IgG isotypes, which were associated with various clinical phenotypes, biochemical or clinical severity profiles, comorbidities, and progression. Presence of some of these autoantibodies in disease controls possibly reveal shared pathophysiology.

### Deconvolution of autoantibody targets identified tissues- and cells of origin

As the immune response is highly specific, the identification of autoantigens might shed some light on basic mechanisms in PSC. Annotations of the human genome allow for precise classification of gene locations and functions, where expression patterns across organs and single-cell types can indicate the specific tissues or cell types targeted.^41^^,^^45^^,^^46^ We therefore combined these two approaches of deconvolution to leverage mechanistic insights from the global autoantibody landscape found.

**Fig. 4:**
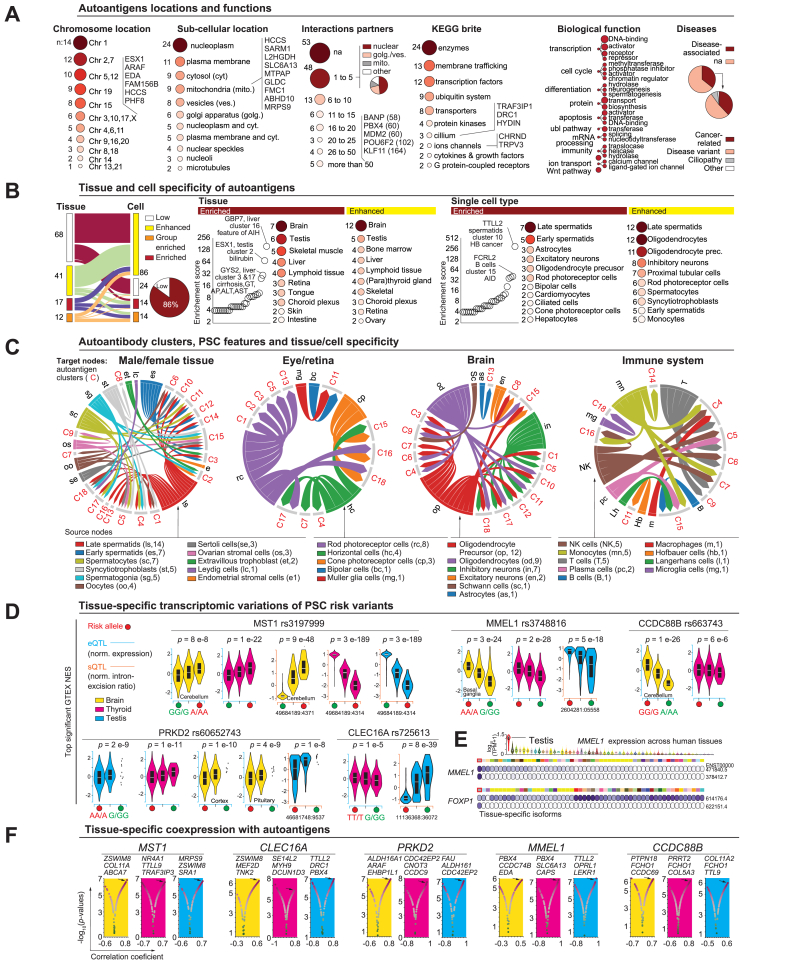
Global deconvolution of autoantibody targets: Locations, functions, cells-of-origin, co-expression with PSC risk variants. (A) Genomic and functional annotations of autoantibody targets. Bubble size and color represent the relative number of autoantigens on each chromosome, their respective sub-cellular locations, the number and location of their interaction partners, and their biological classification according to KEGG Brite. Corresponding numbers of autoantigens are indicated on the left side of the bubbles. Biological functions are depicted using biological processes and molecular function classifiers. The proportion of autoantigens with known associations with diseases are depicted on the right side with pie charts. (B) Tissues and cell types expressing autoantigens at steady state. Sankey diagram indicates the proportion of autoantigens with low, enhanced, or enriched expression in tissues and single cell types. The number of autoantigens found in each category is depicted on both sides of the diagram. Pie chart indicates the overall proportion of those with enhanced or enriched expression in tissue or cells. On its right-side enrichment scores for autoantigens are graphically depicted as well as their main enriched and enhanced locations in tissues and single cell types. The number of autoantigens expressed in these locations are visualized according to the bubble size and color and indicated on their left side. (C) Associations between the PSC features and the locations of autoantigen expression. Chord diagrams for four tissue locations depict associations between cell types expressing the autoantigens in these locations (source node) and their relative autoantibody cluster (target nodes) linked to specific PSC features. The number of autoantigens expressed in each cell type is indicated inside brackets with their respective abbreviations below the chord diagrams. (D) Transcriptomic variations associated with PSC risk variants. Top significant transcriptomic variations in the brain, thyroid and testis linked to PSC risk variants are depicted. Risks alleles are indicated by red filled circles below the violin plots. GTEX eQTL and sQTL are distinguishable by the color of the axis, respectively, blue and orange. Isoform references are indicated below the sQTL graphics. Details on statistics and group size are available on the GTEX portal. (E) Isoform expression of genes nearby PSC risk variants across human tissues. Overview of the expression of *MMEL1* across human tissues. Violin plots are color-coded using the GTEx color scheme, which is also applied to the schematics below, where the expression of MMEL1 and FOXP1 isoforms in each tissue is depicted, with the highest expression shown in a dark shade of purple. (F) Transcriptional correlations between autoantigens and genes nearby PSC risk variants. Graphics show the correlation coefficients and *p* values from comparative transcription analysis in brain, thyroid, and testis between autoantigens and *MST1, CLEC16A, PRKD2, MMEL1,* and *CCDC88B.* Each dot represents an autoantigen with color variations according to *p* values. eQTL, expression quantitative trait loci; na, not annotated; NES, normalized effect size; PSC, primary sclerosing cholangitis; sQTL, splicing quantitative trait loci; TPM, transcripts per million.

We first mapped the autoantigen targets to their respective gene locations (Fig. 4A), but did not detect any significantly enriched chromosomal region. Several genes were located on the X chromosome, possibly of interest regarding the male prevalence in PSC. Interrogating sub-cellular locations, nucleolar autoantigens were overrepresented. Moreover, a set of mitochondrial autoantigens were unraveled. The analysis of their interactome showed that most autoantigens had known interaction partners (Table S6), mainly located in the nucleus, the vesicular system, and the mitochondria. Classification of autoantigens using KEGG Brite^47^ identified a set of transcriptions factors, but also proteins related to the ubiquitin system. In addition, nine modules, 52 networks, and 71 diseases were identified (Table S7). Autoantigens involved in the cilium and ion channels – two key homeostatic structures of cholangiocytes – were identified, as were autoantigens related to cytokines, growth factors (CCL26, EDA), and G-protein–coupled receptors (OPRL1, CRHR2), which are known to be targeted by autoantibodies in other contexts.^48^ Looking at biological processes and molecular functions encompassed by the autoantigens, the cell cycle and Wnt pathway (MCC and SOSTDC1) were identified. More surprisingly, differentiation processes linked to neurogenesis and spermatogenesis were found. Finally, interrogating known disease associations, a third of the autoantigens (source Human Protein Atlas database) were found to be associated with human disorders (90% using DisGeNET database,^49^ Table S8) mainly disease variants, and cancer, including proto-oncogenes (ARAF1, MDM2) and tumor suppressor gene products (BANP, PYHIN1, TSC1, MCC).

We next mapped the autoantigens to their tissue- and cell-of-origin (Fig. 4B). The Human Protein Atlas^41^ revealed that most of them (86%) were enriched or enhanced in a specific tissue and/or cell type. We therefore undertook a global deconvolution to gain insight into the main tissues and cell types expressing the autoantigens linked to disease severity in PSC. First, looking at tissue-enriched autoantigens we surprisingly found the brain and testis to be the two most represented tissues. However, some other autoantigens show a pattern of expression restricted to the liver, such as GBP7 and GYS2. Second, extending the analysis to enhanced tissue autoantigens, the brain and testis also appeared as the tissue expressing most of the autoantigens. The liver and more surprisingly the retina were also found. Of note, four autoantigens depicted an enhanced expression in the parathyroid gland (EMILIN2, FCHSD1, PBX4, SDC2). Third, refining this deconvolution approach to identify single cell types (both enriched, then enhanced expression), spermatids and oligodendrocytes were identified as the main cell types expressing autoantigens. In the retina, bipolar and photoreceptor cells were identified. The two most cell-type-restricted expressions were TTL2 in spermatids, linked to autoantibody cluster 10 associated with HB cancers, and FCRL2 in B cells, linked to autoantibody cluster 15 associated with AID.

As tissues and cells of origin targeted by autoantibodies might be linked to specific disease features and progression,[34], [35], [36] we performed an integrated analysis of these two components (Fig. 4C). Strikingly male/female tissue autoantigens were linked to all the autoantibody clusters associated with PSC features, suggesting that these body compartments and related cell types could possibly be key players in the disease. In the eye and brain, rod photoreceptor cells and oligodendrocyte precursors were associated with the highest number of autoantibody clusters (8 and 12 clusters, respectively). Finally, an array of autoantigens was expressed mainly by immune cells, including monocytes, NK cells, and T cells, as well as tissue-specific macrophages (Hofbauer, Langerhans and microglia cells).

Since tissues such as the brain, retina, and testis are usually not considered involved in PSC pathogenesis, we focused on one of the most well-characterized aspects of PSC: the genetic risk variants. Besides HLA, a few single nucleotide polymorphisms have been significantly associated with PSC,^50^^,^^51^ which is why we interrogated these risk variants for their tissue-specific imprints across the human body. Strikingly, most of them were associated with profound transcriptional deregulations of the nearby genes in the brain, thyroid, and testis (Fig. 4D, Fig. S2). Surprisingly, this seemed to be a common feature of most of the PSC risk variants. However, risk alleles were not homogenously associated with increased or decreased gene expression. Instead, a complex pattern of region-specific deregulations coupled with isoform switching was observed. For instance, within the brain, PSC risk variants were associated with upregulation of the *MST1* transcript in the cerebellum, downregulation of *PRKD2* in the cortex, and changes in *MST1* isoform relative abundance in the cerebellum, thyroid, and testis. Interestingly, some genes showed tissue-specific isoform expression, particularly in the testis (Fig. 4E), including *MMEL1, FOXP1, BACH2, and SH2B3*, suggesting that expression of certain exons may not be detected outside these organs, which are classically considered immune-privileged. Finally, because PSC risk variants were linked to transcriptional variations and ectopically expressed proteins are thought to trigger autoantibodies,^52^ we performed a tissue-specific co-expression analysis and identified covarying autoantigens (Table S9). These data might provide a second layer of variation linking risk variants to autoimmunity. As an example, upregulation of *MST1* within the cerebellum is highly correlated to *ZSWIM8*, *COL11A*, and *ABCA7* in the same tissue.

With this study, we provide an atlas of autoantigen locations and functions, as well as their tissue- and cell-specific expression in relation to clusters of autoantibodies associated with PSC features and progression. We identified brain, testis, and thyroid tissue transcriptional deregulations as potential hallmarks of PSC, linking genetic risk variants to autoimmunity.

### Autoantibody profiles in relation to time components in PSC

The dynamics of the autoantibody repertoire in AID can reveal pathogenic mechanisms associated with disease onset and progression. We therefore further investigated this interplay in PSC.

**Fig. 5:**
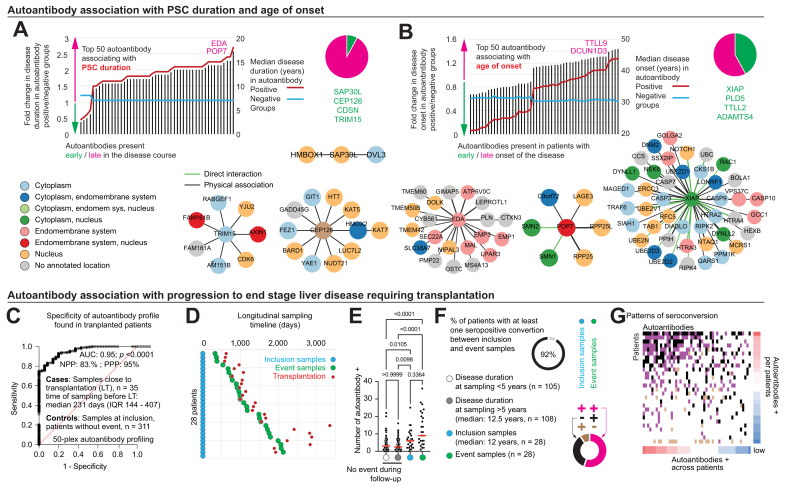
Autoantibody profiles overtime in PSC. (A) Autoantibody association with PSC duration. The medians of disease duration for groups of patients with PSC seropositive and negative for each autoantibody are depicted with red and blue lines (scale on the right side) and corresponding ratios with bar plots (scale on the left axis). The relative proportion of autoantibodies associating preferentially with short (green) or long (pink) disease duration is summarized in the pie chart on the right side. Protein-protein interaction complexes involving those autoantigens (central nodes of star networks) are depicted below, with colors corresponding to their sub-cellular locations. Autoantigens without reported interaction partners are not depicted. (B) Autoantibody association with age of clinical onset of PSC. The median age at disease onset for groups of patients with PSC seropositive and negative for each autoantibody are depicted with red and blue lines (scale on the right side) and corresponding ratios with bar plots (scale on the left axis). The relative proportion of autoantibodies associating preferentially with young (green), or older (pink) age of onset is summarized in the pie chart on the right side. Protein-protein interaction complexes involving XIAP autoantigen (central node of star network) is depicted below, with colors corresponding to sub-cellular locations. (C) Specificity of autoantibody profile found in liver transplanted patients with PSC. Receiver-operating characteristic curve of autoantibody profiles comparing transplanted (n = 35) and non-transplanted patients (n = 311). The top 50 significant autoantibodies associated with transplantation are considered, like other analyses of this panel. (D) Timeline of matched longitudinal samples analyzed. Each line corresponds to a single patient sampled twice, first at study inclusion (inclusion samples, blue filled circles) and second at time (event samples, green filled circles) closest to transplantation (red filled circles). (E) Autoantibody fluctuation over time. Each dot represents a single patient and the number of autoantibodies detected (medians in red) is graphically represented for various groups of patients with PSC. Mann-Whitney tests were used. Doughnut pie charts display information on individual serological changes between matched inclusion and event samples. The proportion of patients with any seropositive conversion is depicted on the top. The relative proportions of seropositive (black) conversions, seronegative (brown) conversions, and preexisting stable seropositivity (pinks) are depicted below it. (F) Proportions of seroconversions. Preexisting autoantibodies as well as positive and negative seroconversions between the inclusion and event samples are displayed. (G) Individual diversity and dynamics of autoantibody profiles over time. Each line represents a patient and each column an autoantibody. The variations between inclusion and event samples for each individual and autoantibody is color coded as indicated in the doughnut pie chart. NPP, negative predictive power; PPP, positive predictive power; PSC, primary sclerosing cholangitis.

First, we selected the top 50 autoantibodies associated with PSC duration and evaluated whether these were present in early or later stages of the disease. Strikingly, 46 (92%) of them were associated with later stages, with PSC duration from 10 to 20 years (Fig. 5A). In patients positive for anti-TRIM5 and anti-CDSN, the median PSC duration was about 3 years, whereas it was 16 and 18 years for those positive for anti-POP7 and anti-EDA. These few autoantibodies arising early in the disease course could provide insight into the initial steps of the autoimmune processes. Out of these four autoantigens, SAP30L and TRIM1 were suggested to be involved in response to viral infection,[53], [54], [55] commonly hypothesized to be an autoimmune trigger. CEP126 plays a role in cilium assembly^56^ and CDSN is known to be involved in skin diseases with a related gene located in the MHCI region. Looking at their interaction partners in relation to autoantibodies found at later stages of PSC, we did not find strong evidence that autoantibody response would spread to proteins within the same complexes. We next characterized autoantibody specificities in relation to age at disease onset and identified a profile associated with early onset of PSC (Fig. 5B). Patients seropositive for anti-XIAP, anti-PLD5, anti-TTLL2 and anti-ADAMTS4 had their PSC diagnosis at a median age of 21 years, whereas those positive for anti-TTLL9 and anti-DCUN1D3 were diagnosed at 44 years. Overall, autoantibodies associated with disease duration were almost all different from those associated with age of onset. These results suggest that distinct autoantibodies associate with early onset of PSC and increase in number over time.

As PSC in general is a slowly progressive disease often spanning over several decades, the duration of disease does not recapitulate entirely its severity spectrum. PSC progression ultimately leads to end-stage liver disease requiring liver transplantation. We therefore analyzed autoantibody profiles present in patients close to the time of transplantation. Autoantibodies in end-stage disease were relatively specific compared with those in patients who did not require transplantation during follow-up (Fig. 5C and S3). The autoantibodies associated with transplantation were mostly independent from those associated with PSC duration, but seven of them were associated with longer disease duration, for example EDA. These data suggest that autoantibodies develop during the later severe stages of PSC and are mainly independent of disease duration. To better understand these dynamics, we analyzed matched, paired longitudinal samples collected from the same patients before and at the time of transplantation (Fig. 5D). The analysis revealed that shortly before transplantation, patients with PSC had a much broader antibody repertoire, recognizing in median of nine autoantigens (Fig. 5E). This elevation of the antibody repertoire was already present several years before (inclusion samples, median of six autoantigens) and was significantly higher than in patients with similar disease duration who did not require transplantation during follow-up (median of 2.5 autoantigens). Thus, this widening of the antibody repertoire associated with end-stage disease in PSC was independent of disease duration, suggesting different driving mechanisms. To further refine these observations at a group level we analyzed seroconversions within individuals during the years preceding transplantation (Fig. 5F). We observed that most patients (92%) had seropositive conversions between the two sampling occasions. Only two patients did not seroconvert and were already positive for 14 and 16 autoantibodies at inclusion, respectively. Overall, many autoantibodies present at the time of transplantation were already detectable at inclusion many years before. Seropositive conversions were also observed to a large extent during the years before transplantation. Seronegative conversions were also observed but represented a minority of changes. However, we found a large heterogeneity in the number of autoantibodies present in patients and their patterns of seroconversion (Fig. 5G).

Overall, we identified specific autoantibody repertoires for disease duration and age at onset of PSC together with a distinct profile in end-stage PSC requiring liver transplantation, already present several years before the transplant.

### Neuroendocrine deregulation in PSC

**Fig. 6:**
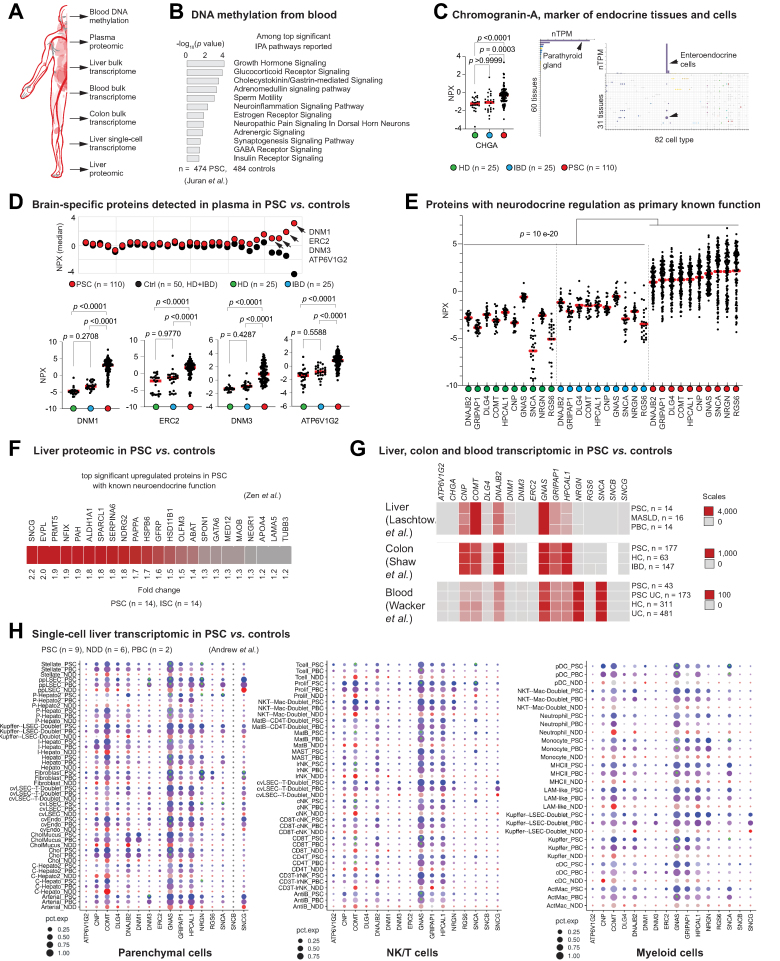
Neuroendocrine deregulations in PSC. (A) Multiomics datasets and body compartments analyzed. (B) Differentially methylated pathways identified by genome-wide methylation profiling linked to neuroendocrine functions. Relative concentration of CHGA (C), brain-specific proteins (D), and other proteins with known neuroendocrine functions (E), in PSC and controls. Mann-Whitney tests were used. (F) Liver proteins with known neuroendocrine functions upregulated in PSC *vs*. controls. (G) Gene expression of the identified proteins in the liver, colon and blood from PSC *vs.* controls. (H) Expression of selected genes across parenchymal and immune cell-types. Red = Healthy (NDD), Purple = PBC, Blue = PSC. Green stars indicate a gene was significantly different (FDR <5%) from the NDD control. HC, healthy controls; HD, healthy donor; IBD, inflammatory bowel disease; IPA, Ingenuity Pathway Analysis; ISC, immunoglobulin (Ig)G4-related sclerosing cholangitis; MASLD, metabolic dysfunction-associated steatotic liver disease; NDD, non-diseased donor; PBC, primary biliary cholangitis; PSC, primary sclerosing cholangitis; UC, ulcerative colitis.

As both the analysis of autoantigens and the QTLs of PSC risk variants pointed towards a neuroendocrine deregulation involved in PSC pathophysiology, we performed a multi-omics and multi-body compartment analysis (Fig. 6A) to further investigate this possibility. Reported differentially methylated pathways from peripheral blood DNA confirmed this perturbation.^57^ We next performed plasma proteomics to determine whether circulating proteins could be involved (Fig. 6B). First focusing on the endocrine biomarker chromogranin A, we found elevated levels in patients with PSC compared to healthy individuals and patients with IBD (Fig. 6C). Then, we asked if some of the recently identified brain-specific proteins (global label score 4)^58^ could also differ in PSC. Among those, we identified DNM1, DNM3, ERC2, and ATPV1G2 at higher concentrations in patients with PSC (Fig. 6D). Extending our search to other proteins with known neuroendocrine functions, we identified ten additional proteins elevated in PSC (Fig. 6E), including synuclein alpha, a marker of Parkinson’s disease.^59^ Interestingly, previous analysis of liver tissue by mass spectrometry reported synuclein gamma as one of the most upregulated proteins in PSC^60^ (Fig. 6F). We finally wondered where these proteins might be produced. Transcriptomic analysis of PSC liver, colon and blood has not indicated major transcriptional deregulations in these tissues[61], [62], [63] (Fig. 6G). However, analysis of PSC livers at a single-cell resolution has revealed that some of these proteins, but not all, might be ectopically overexpressed in a cell-specific manner^64^ (Fig. 6H). Overall, these data uncover a systemic neuroendocrine perturbation in PSC and suggest related ectopic expression in multiple organs.

## Discussion

As the autoimmune component in PSC has been long suspected, many studies have investigated the presence of autoantibodies with the hope of unraveling both basic mechanisms and clinically relevant tools. The liver-centric view has historically been driving study designs; thus, the search for pathogenic autoantibodies in PSC has either focused on hepatocyte and cholangiocyte reactive antibodies, or on evaluation of known autoantibodies described in related diseases. A variety of autoantibodies have been described,^19^ but none of them have shown high specificity and sensitivity. Most importantly, very few autoantibodies show significant, repeated associations with clinical events or disease severity, although anti-glycoprotein 2 has been linked to CCA and poor survival.[65], [66], [67], [68] Anti-smooth muscle, anti-nuclear, and anti-neutrophil cytoplasmic autoantibodies are the most studied but none have been demonstrated to be useful for diagnostic or prognostic purposes. Our approach aimed to move away from the liver-centric approach and consider PSC as a multiorgan disease with systemic perturbations that could potentially drive its progression, as suggested by the high prevalence of other immune driven disorders and high recurrence rate of PSC after transplantation. In addition, none of the PSC risk gene variants have nearby genes that are specifically expressed in the liver.^18^ Thus, the liver immune machinery might not be the major initial player in PSC development.^69^ We therefore screened the entire proteome for autoantibodies associated with PSC severity. However, it remains possible that autoantibodies targeting posttranslational modifications, as well as conformational or linear epitopes, were not captured in our study. Further refinement using alternative protein expression systems or epitope-mapping approaches would be required to extend this search.

Our data suggest that patients with PSC are seropositive for multiple autoantibodies of the IgA and IgG isotypes, which are associated with various clinical phenotypes, biochemical or clinical severity profiles, comorbidities, and disease progression. Some of these autoantibodies seem to also be present in non-PSC disease, as found by us and others, possibly revealing some shared pathophysiology.[70], [71], [72] This pattern of multiple autoantibodies, sometimes unspecific, being shared between related disorders is seen in other contexts such as connective tissue diseases.^71^ However, our results cannot establish a definitive conclusion and would benefit from being investigated with a more targeted approach in independent cohorts. More aspects can be learned from extension of this resource, which spans many overlapping disorders such as AID (including AIH), IgG4-related disease, HB cancers, cirrhosis, and IBD. For instance, an in-depth study of some of these autoantibodies in IBD could more precisely delineate the contribution of intestinal inflammation to PSC and assess autoantibody profiles prior to the clinical onset of PSC. In this context, the impact of colectomy could be studied in more detail as B cells have been identified in intestinal mucosa of patients with PSC, indicating antigen-driven clonal expansion,^62^ with some shared clonotypes located in the liver.^73^ In our case, specific profiles associated with colectomy could suggest a redirection of the autoantibody repertoire in the absence of colonic mucosa.

Whether autoantibodies in PSC are a cause or a consequence of the disease remains to be elucidated. Such distinction is not easy considering the broad autoantibody repertoire uncovered in this study. However, it would be of special importance to develop antigen-specific therapies. Single-cell approaches including B and T-cell receptor (BCR/TCR) sequencing combined with monoclonal autoantibody production and their use in animal or human organoid models could help to decipher the contribution of autoreactive B/T cells and autoantibodies. It may also be useful to investigate B- and T-cell development and tolerance defects using multi-tissue sampling in selected individuals, based on their serological profiles relative to healthy and diseased controls. Some other aspects could be addressed in the context of surgical procedures such as liver transplantation. Autoantibody profiling performed longitudinally pre- and post-transplant in relation to disease recurrence could possibly leverage some mechanistic insights. Of note, two PSC samples were taken after liver transplantation (182 and 202 days post-transplant), with matching samples collected at inclusion (1,883 and 1,919 days before transplantation). In these two patients, preexisting autoantibodies did not disappear, suggesting that autoreactive plasma cells associated with PSC severity may not be primarily located in the liver. Regarding therapies, only one study has previously reported the use of B-cell depletion therapy (rituximab) in PSC, with no recurrence observed in five patients 7 years post-transplantation.^27^ Studies of such therapy could be valuable for better understanding the role of autoantibody profiles and B cells in PSC pathogenesis, although tissue-resident cells may not be reached by these therapies and might have a limited impact on autoantibody levels, as observed in other diseases.^31^

This atlas of autoantigens in PSC identifies locations, functions, as well as their putative tissues and cells of origin. Based on these annotations, the broad landscape of autoantigens encompasses most known functional impairments caused by autoantibodies, including receptor agonism/antagonism, ligand antagonism, cytotoxicity, and immune activation.^74^ Several of these autoantigens are linked to hepatobiliary homeostasis, including the Wnt pathway, cilia, and ion channels. Some are restricted to the liver, others to the mitochondria, a well-known target in other AID. Others relate to pituitary and thyroid glands, and the thyroid also appeared transcriptionally deregulated (eQTLR and sQTL) in relation to PSC risk variants. Beyond the known increased risk of autoimmune thyroid disease in PSC, thyroid dysfunction itself was suggested to be a risk factor for PSC.^75^ Unfortunately, little is known about hormonal dynamics in PSC in relation to disease progression. However, hormones as well as sex are known to influence biliary homeostasis.^76^^,^^77^ Some autoantibodies, previously described in solid tumors,^78^ target oncogenes, tumor suppressor gene products, and testis antigens (so-called cancer-testis antigens), and might play an active role in tumor progression.^79^^,^^80^ In PSC, cholangiocytes harbor a proliferative phenotype in the context of chronic inflammation and development of dysplastic lesions within the bile duct and other organs. Thus, this could recapitulate some similarities with malignant settings generating cancer-testis antigens. Such processes might explain part of an autoantibody profile biased toward cryptic antigens, otherwise expressed in immune-privileged sites at steady state. To further interpret these results, proteomic analysis of various tissues and immune complexes would be of importance to characterize the presence and location of these autoantigens in PSC of various subtypes.

Finally, as in most AID, the trigger of autoimmunity in PSC remains unclear. Beside learning from the minority of AID mainly found in men, the importance of sex in response to infection recently highlighted by the COVID-19 pandemic might be relevant.^81^^,^^82^ Patients with PSC often experience repeated infections that could participate in the expansion of the autoantibody repertoire. However, it is also possible that infection acts as an initial trigger of autoimmunity through molecular mimicry, adjuvant effects, antigenic complementarity, or co-exposure.^83^ Some of the autoantigens we found associated with the first years after PSC diagnosis may play a role in antiviral responses. Moreover, although we initially included the Epstein-Barr virus nuclear antigen 1 as a control, we found elevated levels of anti-Epstein-Barr virus nuclear antigen 1 significantly associated with transplantation. This points once more towards the largely studied and speculative role of Epstein-Barr virus in triggering AID. Among the mechanistic explanations are its impact on myeloid and lymphocytic compartments affecting antigen clearance, presentation, epitope spreading^84^ and molecular mimicry, as recently described in multiple sclerosis.^85^ Unfortunately, Epstein-Barr virus status was not available for patients with PSC or controls in this study. In PSC, the gut microbiota may play a specific role, as IBD often precedes PSC onset. Large-scale analysis of autoantibodies, combined with microbiota antibody profiling^86^ could provide important insights on this matter.

We envision that this resource may have broad implications in hepatology and autoimmunity, as the results span a range of long-standing basic and clinical questions. Among these, but not limited to, are: (i) Autoantigens encoded by the X chromosome, which could provide insights into autoimmune diseases (AIDs) more frequently reported in males, such as PSC, ankylosing spondylitis, autoimmune vasculitis, myocarditis, Kawasaki disease, and Behçet's disease, as well as other AIDs where sex-specific pathophysiology is suspected. (ii) Mitochondrial and nucleolar autoantigens, which are often poorly characterized and present in a variety of AIDs, including PBC, AIH, lupus, Sjögren's syndrome, systemic sclerosis, scleroderma, juvenile arthritis, polymyositis, and dermatomyositis. Publication of the peptide sequences used for their detection may facilitate high-throughput applications. (iii) Autoantigens from cilia and ion channels, which could have pathophysiological relevance in related cholestatic disorders, ciliopathies, or channelopathies, where previously unrecognized autoimmunity may be involved. (iv) Autoantigens known to be associated with disease and cancers at the genetic level, which could have mechanistic implications in PSC, particularly regarding cell proliferation. The ductular reaction – a hallmark of PSC with a high risk of malignant transformation – is also observed in many chronic liver diseases, including PBC, biliary atresia, viral, alcoholic, and non-alcoholic hepatitis, as well as hepatocellular carcinoma. (v) Autoantigens expressed in the testis, where autoantibodies associate with PSC phenotypes and disease progression, including cancer. As cancer-testis antigens share characteristics between tumorigenesis, embryogenesis and spermatogenesis,^80^ they may indicate “diverticular autoimmunity” against the embryonic cecal and hepatic diverticula, a mechanism recently proposed to explain the age-dependent variation in the presentation and progression of PSC.^87^ Such mechanisms could be of importance in other autoimmune and gastroenterological disorders. (vi) Autoantibodies present in the controls groups used in our studies, including ulcerative colitis, Crohn’s disease, benign biliary disorders, CCA, cirrhosis, PBC and AIH. These provide insights into the immune relationship between PSC and a broad range of gastroenterological disorders.

A neuroendocrine compartment in liver diseases has long been postulated, particularly in relation to the ductular reaction and the capacity of cholangiocytes to secrete and respond to a variety of hormones, neuropeptides, and neurotransmitters.^77^ Also, the bile duct is surrounded by a neural network and although we do not yet understand the extent of the crosstalk between the nervous system and the biliary tree, the brain-liver axis is increasingly recognized as important in liver diseases.[88], [89], [90], [91] However, beyond the brain-liver-gut axis, a larger systemic involvement deserves consideration. Here, the analysis of autoantigens, QTLs of PSC risk variants, and multiomics datasets, all pointed towards a systemic neuroendocrine deregulation potentially involved in PSC pathophysiology. Our data uncover peripheral neuroendocrine perturbations in PSC and suggest related ectopic expression in multiple organs. We identified an increased level of synucleins (both alpha and gamma isoforms, respectively in PSC plasma and liver), suggesting that PSC could be a synucleinopathy. Whereas peripheral elevation of the alpha isoform is classically seen in Parkinson’s disease, tissue elevation of the gamma isoform is observed in tumors associated with poor prognosis, including biliary tract cancers.^92^ Interestingly, a model of Parkinson’s disease reported that brain-derived synuclein alpha released in the periphery could accumulate in the liver, promoting local inflammation.^93^ Overall, investigations of the identified proteins in patients with PSC might give new insights into pathophysiological mechanisms and potentially useful tools for clinical monitoring.

Overall, this resource provides new insights into PSC pathogenesis and identifies multiple avenues for further exploration, both in basic mechanisms and clinical applications.

## Abbreviations

AID, autoimmune disease; AIH, autoimmune hepatitis; CCA, cholangiocarcinoma; eQTL, expression quantitative trait loci; HB, hepatobiliary; IBD, inflammatory bowel disease; PBC, primary biliary cholangitis; sQTL, splicing trait loci.

## Authors’ contributions

All authors contributed to the study (technically or intellectually), the review of the manuscript and the interpretation of data. MC and AB designed and financed the study. MC led the data analysis and draft of the manuscript with major contribution of ALB, AB, and DS.

## Data availability

Public access to the data is restricted by Swedish Law and prohibit the release of individual-level datasets that could potentially allow a personal identification. Consequently, only summary-level of such data are allowed to be publicly released. In this study, this particularly concerns the clinical metadata and patients’ s characteristics analyzed in relation to the autoantibodies. However, data access can be granted in the framework of a data transfers agreement. Anyone wishing to gain access to the data can contact Martin Cornillet and Annika Bergquist (martin.cornillet.jeannin@ki.se, annika.bergquist@ki.se).

## Financial support

This project received funding from the 10.13039/501100004359Swedish Research Council (2020-06250 to MC and 2022-01255 to 10.13039/100024877AB), 10.13039/501100018713CIMED (FoUI-962671 to MC and FoUI-973336 to 10.13039/100024877AB), RegionStockholm (RS2020-0731 to 10.13039/100024877AB) and The 10.13039/501100002794Swedish Cancer Society (23 2665 Pj 01 H to 10.13039/100024877AB).

## Conflict of interest

The authors declare no conflicts of interest that pertain to this work. Please refer to the accompanying ICMJE disclosure forms for further details.

## References

[1] Karlsen T.H., Folseraas T., Thorburn D. (Dec 2017). Primary sclerosing cholangitis - a comprehensive review. J Hepatol.

[2] Bowlus C.L., Arrivé L., Bergquist A. (Feb 1 2023). AASLD practice guidance on primary sclerosing cholangitis and cholangiocarcinoma. Hepatology.

[3] Ponsioen C.Y., Assis D.N., Boberg K.M. (Dec 2021). Defining primary sclerosing cholangitis: results from an international primary sclerosing cholangitis study group consensus process. Gastroenterology.

[4] Dyson J.K., Beuers U., Jones D.E.J. (Jun 23 2018). Primary sclerosing cholangitis. Lancet.

[5] Liver EAftSot (2022). EASL clinical practice guidelines on sclerosing cholangitis. J Hepatol.

[6] Cotter J.M., Mack C.L. (Nov 2017). Primary sclerosing cholangitis: unique aspects of disease in children. Clin Liver Dis (Hoboken).

[7] Deneau M.R., El-Matary W., Valentino P.L. (Aug 2017). The natural history of primary sclerosing cholangitis in 781 children: a multicenter, international collaboration. Hepatology.

[8] Rupp C., Rössler A., Zhou T. (Mar 2018). Impact of age at diagnosis on disease progression in patients with primary sclerosing cholangitis. United Eur Gastroenterol J.

[9] van Munster K.N., Bergquist A., Ponsioen C.Y. (Jan 2024). Inflammatory bowel disease and primary sclerosing cholangitis: one disease or two?. J Hepatol.

[10] Visseren T., Erler N.S., Polak W.G. (Aug 2021). Recurrence of primary sclerosing cholangitis after liver transplantation - analysing the European Liver Transplant Registry and beyond. Transpl Int.

[11] Boonstra K., Weersma R.K., van Erpecum K.J. (Dec 2013). Population-based epidemiology, malignancy risk, and outcome of primary sclerosing cholangitis. Hepatology.

[12] Grigoriadis A., Imeen Ringe K., Bengtsson J. (Dec 2022). Development of a prognostic MRCP-score (DiStrict) for individuals with large-duct primary sclerosing cholangitis. JHEP Rep.

[13] Manganis C.D., Chapman R.W., Culver E.L. (Jun 21 2020). Review of primary sclerosing cholangitis with increased IgG4 levels. World J Gastroenterol.

[14] Ricciuto A., Kamath B.M., Griffiths A.M. (Mar 28 2018). The IBD and PSC phenotypes of PSC-IB(D). Curr Gastroenterol Rep.

[15] Weismuller T.J., Trivedi P.J., Bergquist A. (Jun 2017). Patient age, sex, and inflammatory bowel disease phenotype associate with course of primary sclerosing cholangitis. Gastroenterology.

[16] Sarkar S., Bowlus C.L. (Feb 2016). Primary sclerosing cholangitis: multiple phenotypes, multiple approaches. Clin Liver Dis.

[17] Schmeltzer P.A., Russo M.W. (Sep 28 2021). Systematic review of prognostic models compared to the Mayo risk score for primary sclerosing cholangitis. J Clin Med.

[18] Jiang X., Karlsen T.(H. (May 2017). Genetics of primary sclerosing cholangitis and pathophysiological implications. Nat Rev Gastroenterol Hepatol.

[19] Hov J.R., Boberg K.M., Karlsen T.(H. (Jun 28 2008). Autoantibodies in primary sclerosing cholangitis. World J Gastroenterol.

[20] Terjung B., Spengler U. (Apr 2005). Role of auto-antibodies for the diagnosis of chronic cholestatic liver diseases. Clin Rev Allergy Immunol.

[21] Lo S.K., Fleming K.A., Chapman R.W. (Dec 1994). A 2-year follow-up study of anti-neutrophil antibody in primary sclerosing cholangitis: relationship to clinical activity, liver biochemistry and ursodeoxycholic acid treatment. J Hepatol.

[22] Saarinen S., Olerup O., Broome U. (Nov 2000). Increased frequency of autoimmune diseases in patients with primary sclerosing cholangitis. Am J Gastroenterol.

[23] Lundberg Båve A., von Seth E., Ingre M. (Mar 5 2024). Autoimmune diseases in primary sclerosing cholangitis and their first-degree relatives. Hepatology.

[24] Lamberts L.E., Janse M., Haagsma E.B. (Oct 2011). Immune-mediated diseases in primary sclerosing cholangitis. Dig Liver Dis.

[25] Bowlus C.L. (Feb 2014). Primary sclerosing cholangitis: one disease or several?. Clin Liver Dis (Hoboken).

[26] Liu X., Wang H., Liu X. (Sep 2022). Efficacy and safety of immune-modulating therapy for primary sclerosing cholangitis: a systematic review and meta-analysis. Pharmacol Ther.

[27] Yamada Y., Hoshino K., Fuchimoto Y. (Feb 2018). Rituximab induction to prevent the recurrence of PSC after liver transplantation-the lessons learned from ABO-incompatible living donor liver transplantation. Transpl Direct.

[28] Zenouzi R., Lohse A.W. (Nov 2014). Long-term outcome in PSC/AIH "overlap syndrome": does immunosuppression also treat the PSC component?. J Hepatol.

[29] Alberti-Flor J.J., Avant G.R. (Feb 1985). Dunn G(D) Primary sclerosing cholangitis. South Med J.

[30] Lopens S., Krawczyk M., Papp M. (Mar 16 2020). The search for the Holy Grail: autoantigenic targets in primary sclerosing cholangitis associated with disease phenotype and neoplasia. Auto Immun Highlights.

[31] Lee D.S.W., Rojas O.L., Gommerman J.L. (Mar 2021). B cell depletion therapies in autoimmune disease: advances and mechanistic insights. Nat Rev Drug Discov.

[32] Oh S., Mao X., Manfredo-Vieira S. (Sep 2023). Precision targeting of autoantigen-specific B cells in muscle-specific tyrosine kinase myasthenia gravis with chimeric autoantibody receptor T cells. Nat Biotechnol.

[33] Yi J., Miller A.T., Archambault A.S. (Oct 14 2022). Antigen-specific depletion of CD4(+) T cells by CAR T cells reveals distinct roles of higher- and lower-affinity TCRs during autoimmunity. Sci Immunol.

[34] Arbuckle M.R., McClain M.T., Rubertone M.V. (Oct 16 2003). Development of autoantibodies before the clinical onset of systemic lupus erythematosus. N Engl J Med.

[35] Greenberg S.(A. (May 2019). Inclusion body myositis: clinical features and pathogenesis. Nat Rev Rheumatol.

[36] Cornillet M., Sebbag M., Verrouil E. (Jun 2014). The fibrin-derived citrullinated peptide β60-74Cit_60_,_72_,_74_ bears the major ACPA epitope recognised by the rheumatoid arthritis-specific anticitrullinated fibrinogen autoantibodies and anti-CCP2 antibodies. Ann Rheum Dis.

[37] Sjöberg R., Mattsson C., Andersson E. (Sep 25 2016). Exploration of high-density protein microarrays for antibody validation and autoimmunity profiling. N Biotechnol.

[38] Villard C., Friis-Liby I., Rorsman F. (Nov 19 2022). Prospective surveillance for cholangiocarcinoma in unselected individuals with primary sclerosing cholangitis. J Hepatol.

[39] Al-Rabadi L.F., Caza T., Trivin-Avillach C. (Jul 2021). Serine protease HTRA1 as a novel target antigen in primary membranous nephropathy. J Am Soc Nephrol.

[40] Arve-Butler S., Mossberg A., Kahn F. (2022). Identification of novel autoantigens as potential biomarkers in juvenile idiopathic arthritis associated uveitis. Front Pediatr.

[41] Uhlén M., Fagerberg L., Hallström B.M. (Jan 23 2015). Proteomics. Tissue-based map of the human proteome. Science.

[42] Cornillet M., Villard C., Rorsman F. (Apr 2024). The Swedish initiative for the study of Primary sclerosing cholangitis (SUPRIM). EClinicalMedicine.

[43] Altman D.G. (October 1991). Practical statistics for medical research. Chapman and Hall.

[44] Welsh A.H., Sheskin David J. (2003). Handbook of parametric and nonparametric statistical procedures.

[45] Uhlen M., Oksvold P., Fagerberg L. (Dec 2010). Towards a knowledge-based human protein atlas. Nat Biotechnol.

[46] (Jun 2013). The genotype-tissue expression (GTEx) project. Nat Genet.

[47] Kanehisa M., Goto S. (Jan 1 2000). KEGG: kyoto encyclopedia of genes and genomes. Nucleic Acids Res.

[48] Cabral-Marques O., Riemekasten G. (Nov 2017). Functional autoantibodies targeting G protein-coupled receptors in rheumatic diseases. Nat Rev Rheumatol.

[49] Piñero J., Ramírez-Anguita J.M., Saüch-Pitarch J. (Jan 8 2020). The DisGeNET knowledge platform for disease genomics: 2019 update. Nucleic Acids Res.

[50] Ji S.G., Juran B.D., Mucha S. (Feb 2017). Genome-wide association study of primary sclerosing cholangitis identifies new risk loci and quantifies the genetic relationship with inflammatory bowel disease. Nat Genet.

[51] Liu J.Z., Hov J.R., Folseraas T. (Jun 2013). Dense genotyping of immune-related disease regions identifies nine new risk loci for primary sclerosing cholangitis. Nat Genet.

[52] Wang D., Zhang Y., Meng Q. (2020). AAgAtlas 1.0: a database of human autoantigens extracted from biomedical literature. Methods Mol Biol.

[53] Shen Z., Wei L., Yu Z.B. (2021). The roles of TRIMs in antiviral innate immune signaling. Front Cell Infect Microbiol.

[54] Viiri K.M., Jänis J., Siggers T. (Jan 2009). DNA-binding and -bending activities of SAP30L and SAP30 are mediated by a zinc-dependent module and monophosphoinositides. Mol Cell Biol.

[55] Le May N., Mansuroglu Z., Léger P. (Jan 2008). A SAP30 complex inhibits IFN-beta expression in Rift Valley fever virus infected cells. Plos Pathog.

[56] Bonavita R., Walas D., Brown A.K. (Aug 2014). Cep126 is required for pericentriolar satellite localisation to the centrosome and for primary cilium formation. Biol Cell.

[57] Juran B.D., McCauley B.M., Atkinson E.J. (Aug 1 2024). Epigenetic disease markers in primary sclerosing cholangitis and primary biliary cholangitis-methylomics of cholestatic liver disease. Hepatol Commun.

[58] Malmström E., Malmström L., Hauri S. (May 15 2025). Human proteome distribution atlas for tissue-specific plasma proteome dynamics. Cell.

[59] Agin-Liebes J., Lodge A., Reddy H. (Aug 2025). α-synuclein biomarker assays: bridging research and patient care. Lancet Neurol.

[60] Zen Y., Britton D., Mitra V. (May 2016). A global proteomic study identifies distinct pathological features of IgG4-related and primary sclerosing cholangitis. Histopathology.

[61] Laschtowitz A., Lindberg E.L., Liebhoff A.M. (Mar 2025). Liver transcriptome analysis reveals PSC-attributed gene set associated with fibrosis progression. JHEP Rep.

[62] Shaw D.G., Aguirre-Gamboa R., Vieira M.C. (Jun 2023). Antigen-driven colonic inflammation is associated with development of dysplasia in primary sclerosing cholangitis. Nat Med.

[63] Wacker E.M., Uellendahl-Werth F., Bej S. (Feb 2024). Whole blood RNA sequencing identifies transcriptional differences between primary sclerosing cholangitis and ulcerative colitis. JHEP Rep.

[64] Andrews T.S., Nakib D., Perciani C.T. (May 2024). Single-cell, single-nucleus, and spatial transcriptomics characterization of the immunological landscape in the healthy and PSC human liver. J Hepatol.

[65] Jendrek S.T., Gotthardt D., Nitzsche T. (Jan 2017). Anti-GP2 IgA autoantibodies are associated with poor survival and cholangiocarcinoma in primary sclerosing cholangitis. Gut.

[66] Sowa M., Kolenda R., Baumgart D.C. (2018). Mucosal autoimmunity to cell-bound GP2 isoforms is a sensitive marker in PSC and associated with the clinical phenotype. Front Immunol.

[67] Wunsch E., Norman G.L., Milkiewicz M. (Jan 2021). Anti-glycoprotein 2 (anti-GP2) IgA and anti-neutrophil cytoplasmic antibodies to serine proteinase 3 (PR3-ANCA): antibodies to predict severe disease, poor survival and cholangiocarcinoma in primary sclerosing cholangitis. Aliment Pharmacol Ther.

[68] Tornai T., Tornai D., Sipeki N. (Jan 10 2018). Loss of tolerance to gut immunity protein, glycoprotein 2 (GP2) is associated with progressive disease course in primary sclerosing cholangitis. Sci Rep.

[69] Horst A.K., Kumashie K.G., Neumann K. (Jan 2021). Antigen presentation, autoantibody production, and therapeutic targets in autoimmune liver disease. Cell Mol Immunol.

[70] Himoto T., Nishioka M. (Aug 2013). Autoantibodies in liver disease: important clues for the diagnosis, disease activity and prognosis. Auto Immun Highlights.

[71] Didier K., Bolko L., Giusti D. (2018). Autoantibodies associated with connective tissue diseases: what meaning for clinicians?. Front Immunol.

[72] Wang D., Yang D., Yang L. (Jun 2 2023). Human autoantigen atlas: searching for the hallmarks of autoantigens. J Proteome Res.

[73] Chung B.K., Henriksen E.K.K., Jørgensen K.K. (Aug 2018). Gut and liver B cells of common clonal origin in primary sclerosing cholangitis-inflammatory bowel disease. Hepatol Commun.

[74] Pisetsky D.S. (Aug 2023). Pathogenesis of autoimmune disease. Nat Rev Nephrol.

[75] Zhang W., Lang R. (2023). Genetic link between primary sclerosing cholangitis and thyroid dysfunction: a bidirectional two-sample Mendelian randomization study. Front Immunol.

[76] Phelps T., Snyder E., Rodriguez E. (Nov 27 2019). The influence of biological sex and sex hormones on bile acid synthesis and cholesterol homeostasis. Biol Sex Differ.

[77] Alvaro D., Mancino M.G., Glaser S. (Jan 2007). Proliferating cholangiocytes: a neuroendocrine compartment in the diseased liver. Gastroenterology.

[78] de Jonge H., Iamele L., Maggi M. (Feb 15 2021). Anti-cancer auto-antibodies: roles, applications and open issues. Cancers (Basel).

[79] Ai H., Yang H., Li L. (2023). Cancer/testis antigens: promising immunotherapy targets for digestive tract cancers. Front Immunol.

[80] Nin D.S., Deng L.W. (Mar 17 2023). Biology of cancer-testis antigens and their therapeutic implications in cancer. Cells.

[81] Klein S.L., Flanagan K.L. (Oct 2016). Sex differences in immune responses. Nat Rev Immunol.

[82] Takahashi T., Ellingson M.K., Wong P. (Dec 2020). Sex differences in immune responses that underlie COVID-19 disease outcomes. Nature.

[83] Root-Bernstein R., Fairweather D. (Jun 21 2015). Unresolved issues in theories of autoimmune disease using myocarditis as a framework. J Theor Biol.

[84] Jog N.R., James J.(A. (2020). Epstein barr virus and autoimmune responses in systemic lupus erythematosus. Front Immunol.

[85] Lanz T.V., Brewer R.C., Ho P.P. (Mar 2022). Clonally expanded B cells in multiple sclerosis bind EBV EBNA1 and GlialCAM. Nature.

[86] Vogl T., Klompus S., Leviatan S. (Aug 2021). Population-wide diversity and stability of serum antibody epitope repertoires against human microbiota. Nat Med.

[87] Kellermayer R., Carbone M., Horvath T.D. (May 14 2024). Identifying a therapeutic window of opportunity for people living with primary sclerosing cholangitis: embryology and the overlap of inflammatory bowel disease with immune-mediated liver injury. Hepatology.

[88] Adori C., Daraio T., Kuiper R. (Jul 2021). Disorganization and degeneration of liver sympathetic innervations in nonalcoholic fatty liver disease revealed by 3D imaging. Sci Adv.

[89] Tanimizu N., Ichinohe N., Mitaka T. (Apr 25 2018). Intrahepatic bile ducts guide establishment of the intrahepatic nerve network in developing and regenerating mouse liver. Development.

[90] Mravec B., Szantova M. (Nov 2024). The role of the nervous system in liver diseases. Hepatol Res.

[91] Wu H., Zhang Y., Yu J. (2023). Editorial: gut-liver-brain axis: a complex network influences human health and diseases. Front Neurosci.

[92] Takemura Y., Ojima H., Oshima G. (Aug 2021). Gamma-synuclein is a novel prognostic marker that promotes tumor cell migration in biliary tract carcinoma. Cancer Med.

[93] Reyes J.F., Ekmark-Léwen S., Perdiki M. (Mar 20 2021). Accumulation of alpha-synuclein within the liver, potential role in the clearance of brain pathology associated with Parkinson's disease. Acta Neuropathol Commun.

