# Supplementary material for: Proteome-scale autoantibody profiling in PSC: Associations with clinical phenotypes and evidence for neuroendocrine deregulations
Source: JHEP Rep. 2025 Dec 23;8(3):101719. doi: 10.1016/j.jhepr.2025.101719 (PMC12925457; doi:10.1016/j.jhepr.2025.101719)
Supplement: Multimedia component 1 [file mmc1.pdf]

Proteome-scale autoantibody profiling in PSC: Associations with clinical phenotypes and evidence for neuroendocrine deregulations

Martin Cornillet, Aiva Lundberg Båve, Dan Sun, Ghada Nouairia, Christina Villard, Aristeidis Grigoriadis, Erik von Seth, Hannes Jansson, María Bueno Álvez, Sofia Bergström, Peter Nilsson, Mathias Uhlén, Fredrik Edfors, Per Stål, SweHep consortium, Ernesto Sparrelid, Niklas Björkström, Jonas Halfvarson, Annika Bergquist

Table of contents

Fig. S1 2

Fig. S2 3

Fig. S3 26

Supplementary materials and methods 27

Fig. S1_patients_groups

Fig. S2_GTEX_SNP

Fig. S3_prospective_ROC

Supplementary materials and methods

Planar arrays

The samples (sera pools) were diluted 1:12.5 in assay buffer, incubated in assay buffer for 15 minutes at room temperature, then transferred to the slides containing the antigen arrays to incubate for one hour at room temperature on the bench without shaking. For autoantibody quantification, anti-human IgA Alexa 647 (α chain specific, Jackson, 109-605-011, 1.4 mg/ml, diluted 1:15,000) and goat anti-human IgG Alexa 647 (H+L, Life Technology, #A21445, 2 mg/ml, diluted 1:15,000) were sequentially incubated in the dark for one hour at room temperature on a shake table. The slides were scanned using a CapitalBio LuxScan HT24 instrument following each incubation. The values from the IgA scan were subtracted from the resulting values from the IgG scan, to estimate reactivities unique for IgG. A comparison to an internal database was also made per immunoglobulin type (IgA: three pools. IgG: 22 individual samples and 20 pools) to give an indication if reactive antigens were selectively reactive in this study or more generally reactive.

Planar arrays - Production of protein fragments

The protein fragments were produced within the HPA project. These were 20–200 amino acids in length and recombinantly produced in Escherichia coli Rosetta DE3 strain. These protein fragments were chosen from their respective protein due to having low sequence similarity compared to other human proteins, while also avoiding transmembrane regions and signal peptides. They contained an N-terminal hexa-histidine albumin binding protein tag (His6ABP) for purification and solubility purposes. Purification of the protein fragments was performed by affinity chromatography and verified using mass spectroscopy.

Planar arrays - Array production

The protein fragments were diluted into 384-well microplates using a liquid handling robot (Freedom Evo, Tecan Group Ltd) in a buffer containing 0.05 M carbonate-bicarbonate, pH = 9.6 (Medicago AB), and 50% glycerol (Merck KGaA). After dilution and transfer of the protein fragments into 384-well microplates, the plates were stored at -20C until use. Using a noncontact microarray printer (Arrayjet Marathon, ArrayJet Ltd), each of the protein fragment-containing microplates was arrayed on epoxide-coated glass slides (Epoxysilane E, Schott) with a total of 58,752 divided on to two slides. These 58,752 features correspond to 42,100 unique protein fragments which represent 18,955 human Ensembl Gene IDs (Ensembl Release 112, May 2024). After arraying, the slides were dried in a heat cabinet at 37C over night prior to blocking with 3% bovine serum albumin (Saveen Werner AB) for 1 hour. After blocking, the slides are washed twice in PBS (Medicago AB) with 0.1% Tween20 (BDH Prolabo) (PBS-T) for 5 min, each followed by a 5 min wash in PBS, dried in a centrifuge and stored at 4C until use.

Planar arrays - Data Processing

Results from the image analysis were background subtracted using the local background. The data was filtered for features that were negative after background subtraction, features that had been flagged as bad or not found during image analysis, features that were less than 30 pixels in size, and features not being above 10 times the standard deviation of the local feature control channel background. After filtration, the data was transformed per sample to the number of standard deviations each spot differed from the mean. The data from the two arrays were then combined, using the feature with highest feature control signal for replicates. The resulting values from the

IgA scan (filtered features set to 0) were subtracted from the resulting values from the IgG scan, to estimate reactivities unique for IgG. The data is continuous with no clear unbiased differentiation between reactivity and non-reactivity. A comparison to an in-house database was also made per immunoglobulin type (IgA: 3 pools. IgG: 22 individual samples and 20 pools) to give an indication if reactive antigens are selectively reactive in this study or more generally reactive. Correlation of the feature control signal between the two scans, separated by pool and colored by filtering status, demonstrate good reproducibility between scans and image analysis. Due to some features being reactive for IgG but not IgA, and therefore avoiding filtering after the IgG scan although they may have been filtered in the IgA scan, only the features filtered for both scans will be excluded completely from the data analysis.

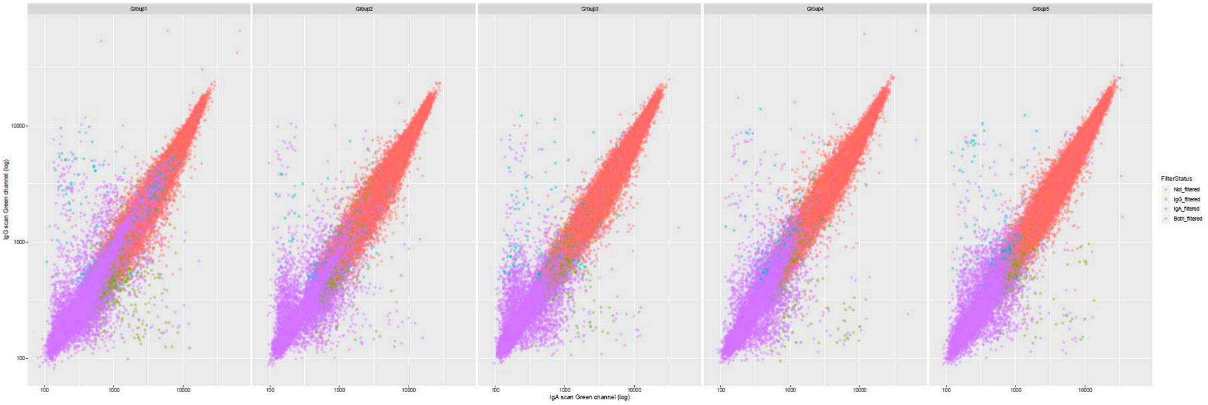

Bead arrays

The diluted samples were mixed with the SBA and incubated for two hours. Any antibody binding to the beads were fixated by incubating the beads in 0.2% paraformaldehyde for 10 min. For the assays measuring IgG, Goat anti-Human IgG Fc Secondary Antibody PE (12-4998-82, eBioscienceTM, Invitrogen), and for the assay measuring IgA, Goat anti-Human IgA

Antibody DyLight. 550 Conjugated (A80-102D3, Bethyl Laboratories), was then applied for 30 min to enable a readout using a FlexMap 3D instrument (Luminex Corp., Austin, TX).

Bead arrays - Antigens

The data is based on 3 suspension bead arrays (SBAs) including 985 unique antigens and 5 technical controls. All antigens (except for the controls) are protein fragments called Protein Signature Tags (PrESTs) expressed in Escherichia coli with a six histidine and albumin binding protein tag (His6ABP), and have been produced within the Human Protein Atlas project (https://www.proteinatlas.org/). The PrESTs have been designed to not contain transmembrane regions and to have less than 60% sequence similarity (on gene-level) towards any other human gene. If the sequence similarity criteria have failed to be fulfilled fragments mapping to a group of similar proteins have been produced instead. In the latter case one of the genes the fragment maps to is included with an asterisk (*) afterwards in the shorthand identifiers, and the full list of genes the fragment maps to is found in the antigen metadata. Regarding the selection of antigens, the overall strategy was to select the higher IgG and IgA reactivities (AAB titers) in the first phase (planar array), within the technical limitation of a maximum number of 380 antigens per batch of beads for the second phase. The IgG gave relatively high signals, and we selected the top 180 higher reactivities in each group to be validated in the second phase. Due to technical limitation in the maximum number of antigens per batch, we had to remove a few (the 17 weakest) to be able to run them in two batches of beads. The IgA gave much lower signals in general, as expected. We included in this case only the top 100 higher reactivities per group to be run in one batch of beads. In our experience, as the IgA signals are much lower than the IgG signals in such methods, we performed a sequential detection of

the IgA then IgG, consequently giving additive/cumulative signal in the planar arrays when scanning the IgG signal. For this reason, we couldn't exclude a risk that a high IgA signal would mask part of an IgG signal. To avoid such bias, we therefore included the top highest IgA signals in each group to be also tested toward IgG in the second phase.

Bead arrays - assays

Three assays were prepared separately, one for IgA and two for IgG (IgG1 and IgG2). The antigens and technical controls were covalently coupled to color-coded magnetic beads (MagPlex, Luminex Corp., Austin, TX) using NHS and EDC based chemistry. The controls consisted of His6ABP (negative, tag binding control for the protein fragments; Human Protein Atlas), buffer (negative, bead binding control), Goat anti Human IgG (positive, sample loading control; 309-005-082, Jackson immunoresearch), Goat anti Human IgA (affinity purified) (positive, sample loading control; GA-80A, Immune Systems), and EBNA1 (semi positive control; ab138345, abcam). The samples were diluted 1:250 in assay buffer (3% BSA and 5% milk powder in PBS supplemented with 0.05% Tween-20 and 0.16 mg/ml His6ABP tag). Commercial plasma (mixed 50% male & female pool; HUMANPLK2PNN, BioIVT) was diluted like the test samples and added to the dilution plates as technical controls. The diluted samples were split in three aliquots and frozen at -20C. In total, six assays were performed, two per SBA and day, as follows. One frozen aliquot of the diluted samples was thawed at +4C, and when thawed they incubated for 15 min at room temperature for the His6ABP in the buffer to pre-block any potential antibodies toward the tag (derived from Streptococcal protein G). Subsequently, the diluted samples were mixed with the SBA and incubated for 2 hours. Any antibody binding to the beads

were fixated by incubating the beads in 0.2% paraformaldehyde for 10 min. For the assays measuring IgG, Goat anti-Human IgG Fc Secondary Antibody PE (12-4998-82, eBioscienceTM, Invitrogen), and for the assay measuring IgA, Goat anti-Human IgA Antibody DyLight. 550 Conjugated (A80-102D3, Bethyl Laboratories), was then applied for 30 min to enable a readout using a FlexMap 3D instrument (Luminex Corp., Austin, TX). The readout consists of the median fluorescence intensity (MFI) and number of beads for each antigen (bead ID) in each sample (the MFI is the median of the signals from individual beads with the same bead ID). Samples were preserved in -80C. Before the assay, the samples were randomly spread in 96-well plates sealed with aluminum adhesive and preserved at -20C. Upon thawing and removal of the sealing, spill of sample might have occurred, but the extent to which it might affect the data is difficult to determine. During the readout of the assay plates IgA P2 and IgG2 P1, the FlexMap 3D instruments aborted the runs after well L21 and C4, respectively, but were directly restarted to continue the read out. Hence, the bead count is lower in the affected wells.

Bead arrays - Data handling

Information about the different quality control and data processing steps are described below as well as more mathematical aspects of the data processing. The antigens have antigen specific background levels, causing potential bias during the sample specific transformation (see below) and making comparisons between antigens difficult. To account for this, the data can be centered so that the 10th percentile of each antigen (excluding filtered samples, control wells, and positive control beads) is equal to the 10th percentile of the entire data set (excluding filtered samples/antigens, control wells, and positive control beads). The raw MFI is denoted MFIorg and the

percentile adjusted MFI is denoted MFIadj in the data files. The samples also have sample specific background levels, making direct comparisons between the MFI values skewed. Using the assumption that in any given sample, a portion of the antigens will not be reactive, an estimate background level can be found per sample to center the data. Here, the antigen percentile adjusted MFI values can be transformed per sample into number of “median absolute deviations” (MADs) around the sample median (assuming that median reflects the sample specific background). This is comparable to samplewise robust z-score but with the constant set to 1: MADsag,sample = (MFIadjag,sample−mediansample)/MADsample. In the plot below MFIorg, MFIadj, and MASs might be depicted. For the study we used the MFIorg (raw) to be as close as possible to the real-life setting, where each result for each antigen and each patient is not influenced by the reactivity of other antigens or other samples.

Bead arrays - Quality control - Coupling test

All beads are coupled in parallel, per SBA, in four 96-well plates. All PrESTs contain a common tag that is used to check the general efficiency of the coupling. The signal from the anti-tag test can vary a lot between PrESTs, so there is no general cutoff level for passing the test. However, signals between good couplings correlate well and this is used as a general check for the quality of the coupling. Since a lack of signal from the tag-test can be due to sterical hindrance (anti-tag antibody not reaching its epitopes) rather than lack of antigen, no antigens are filtered based on the test but antigens with signals in level or below the empty bead are flagged. This means that if the corresponding beads demonstrate interesting results when analyzing samples, extra care should be taken to evaluate and validate the results.

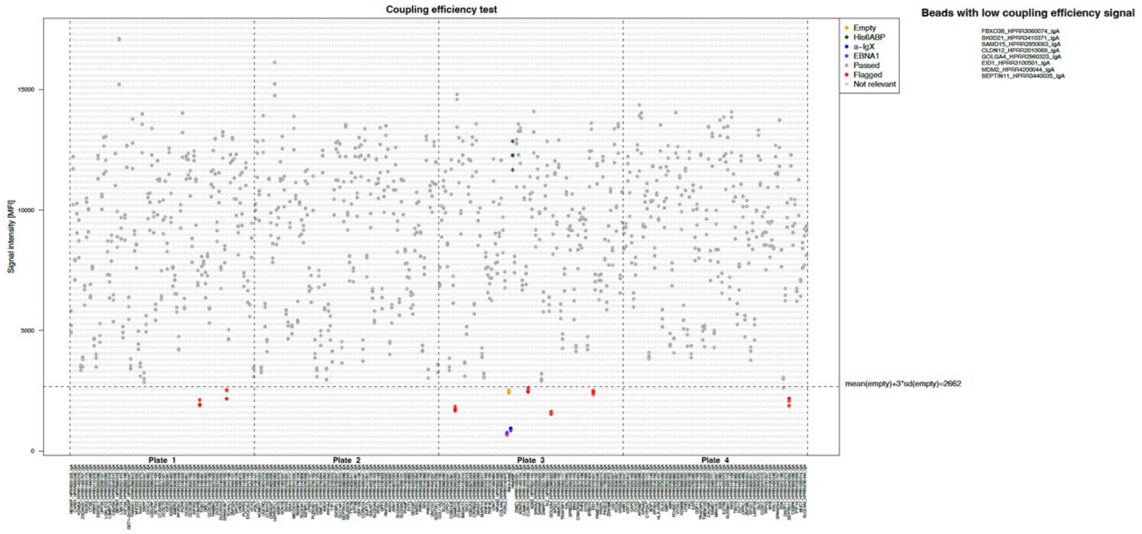
Coupling efficiency test
Signal intensity [MFI]
0
5000
10000
15000
mean(empty)+3*sd(empty)=2662
Plate 1
Plate 2
Plate 3
Plate 4
Empty
His6ABP
a–IgX
EBNA1
Passed
Flagged
Not relevant
Beads with low coupling efficiency signal
FBXO38_HPRR3060074_IgA
SH3D21_HPRR3410371_IgA
SAMD15_HPRR2850063_IgA
CLDN12_HPRR2010069_IgA
GOLGA4_HPRR2960323_IgA
EID1_HPRR3100501_IgA
MDM2_HPRR4200044_IgA
SEPTIN11_HPRR3440035_IgA

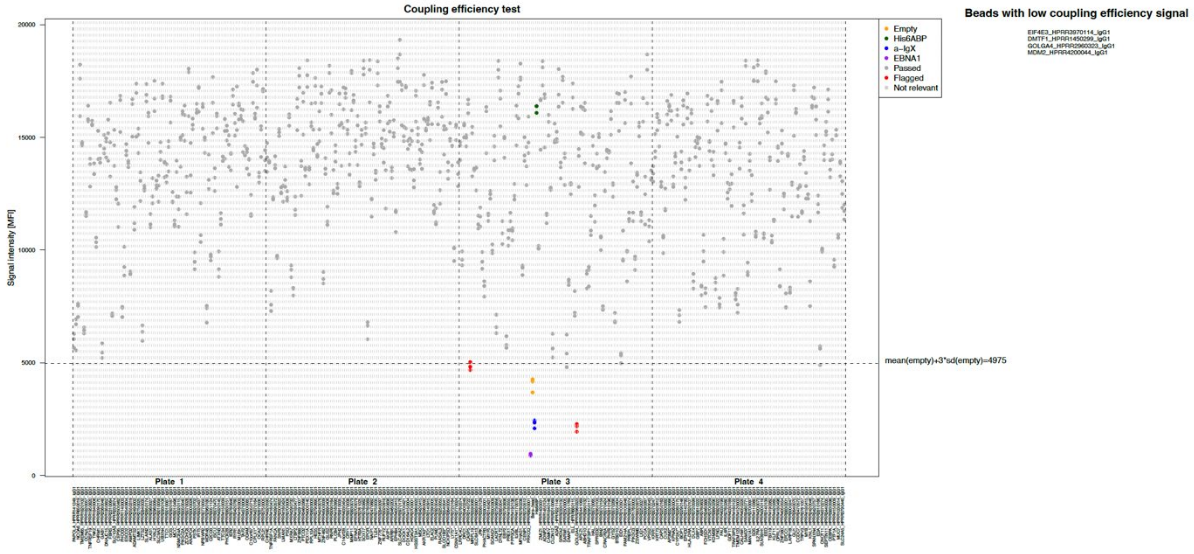
Coupling efficiency test
Signal intensity [MFI]
0
5000
10000
15000
20000
mean(empty)+3*sd(empty)=4975
Plate 1
Plate 2
Plate 3
Plate 4
Empty
His6ABP
a–IgX
EBNA1
Passed
Flagged
Not relevant
Beads with low coupling efficiency signal
EIF4E3_HPRR3970114_IgG1
DMTF1_HPRR1450299_IgG1
GOLGA4_HPRR2960323_IgG1
MDM2_HPRR4200044_IgG1

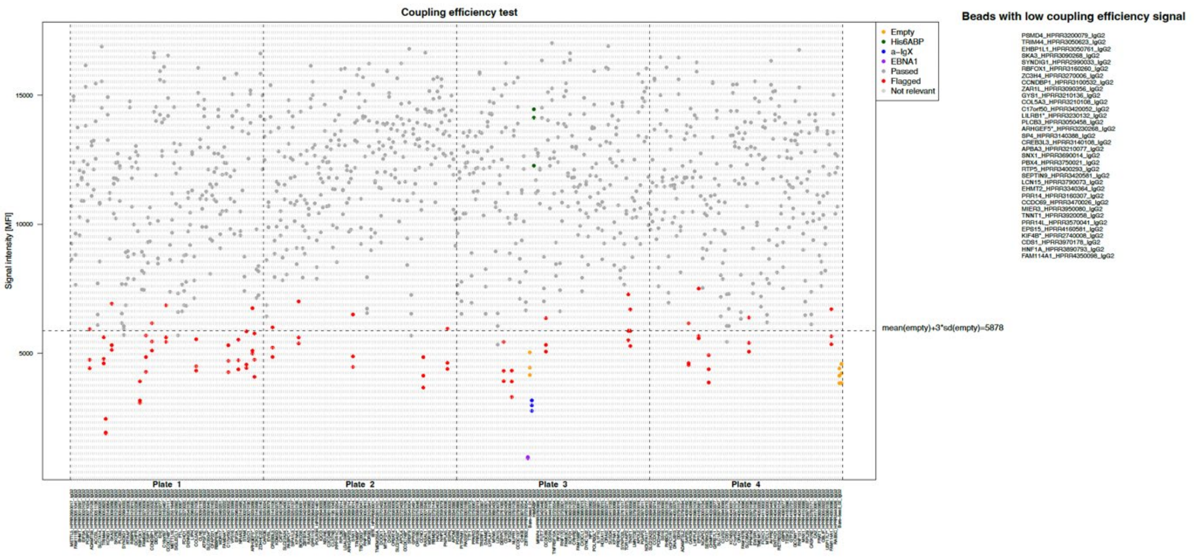

Bead arrays - Quality control - Bead count

Each bead ID is present with hundreds of beads in the suspension bead array (SBA) that is mixed with the samples. The instrument measures a fluorescence intensity from every single bead that passes through the lasers, and the final reported median fluorescence intensity (MFI) is then the median of these individually measured intensities for each specific bead ID. To make sure that the reported MFI is an accurate reflection of the true intensity value, enough individual beads need to be measured. At least 35 beads per bead ID and sample is recommended, however, data points from bead ID and sample combinations with a lower count can also be used but with some extra caution. Depending on the study one may ether introduce NAs in the data or remove samples and/or bead IDs completely to have sufficient count in the data used downstream. The later is done here. The horizontal dashed line in the sample wise plots correspond to 35. The black horizontal dashed line in the sample wise plots correspond to 35 and the red to 16. The vertical lines mark after which wells a short wash was performed in the readout instruments. The box outline color in the sample

wise plot groups wells that received beads in the same bead dispensing round (adjacent boxes of same color).

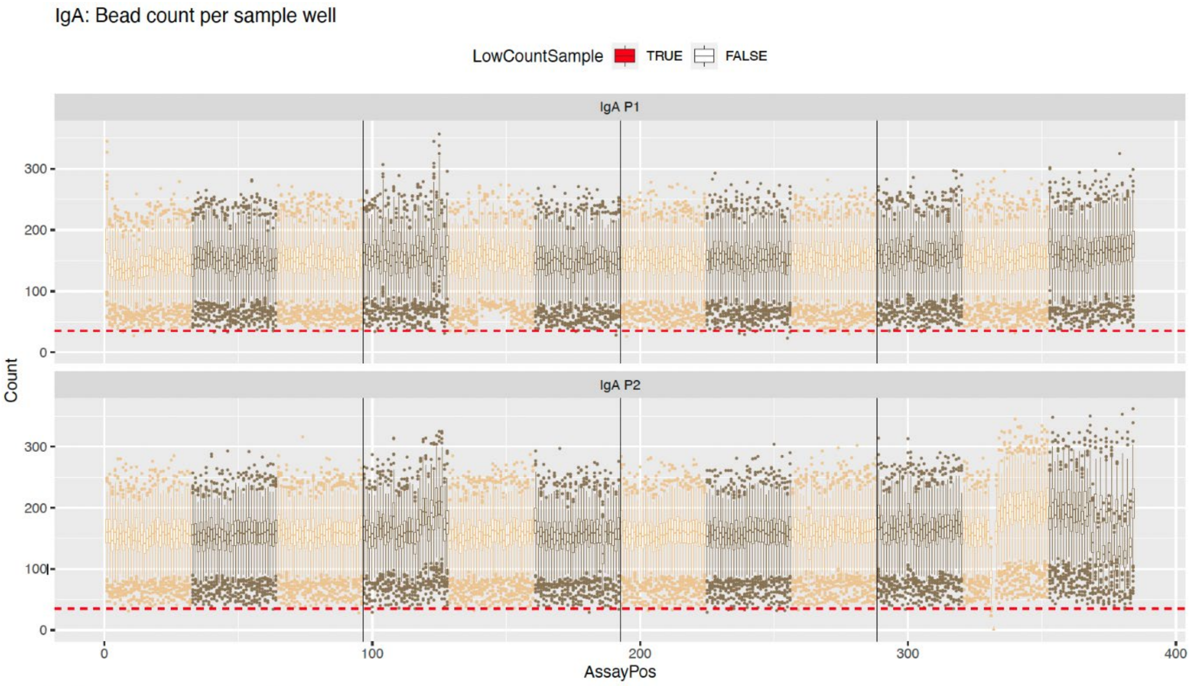

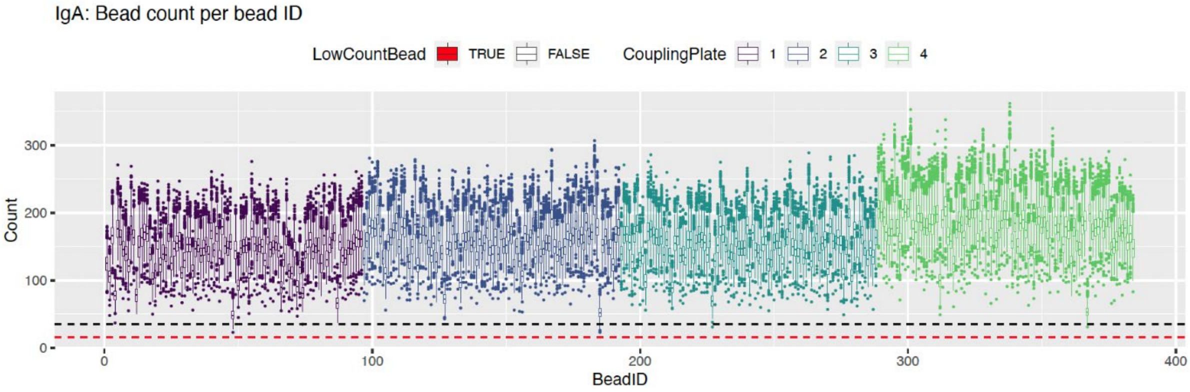

IgG1: Bead count per sample well

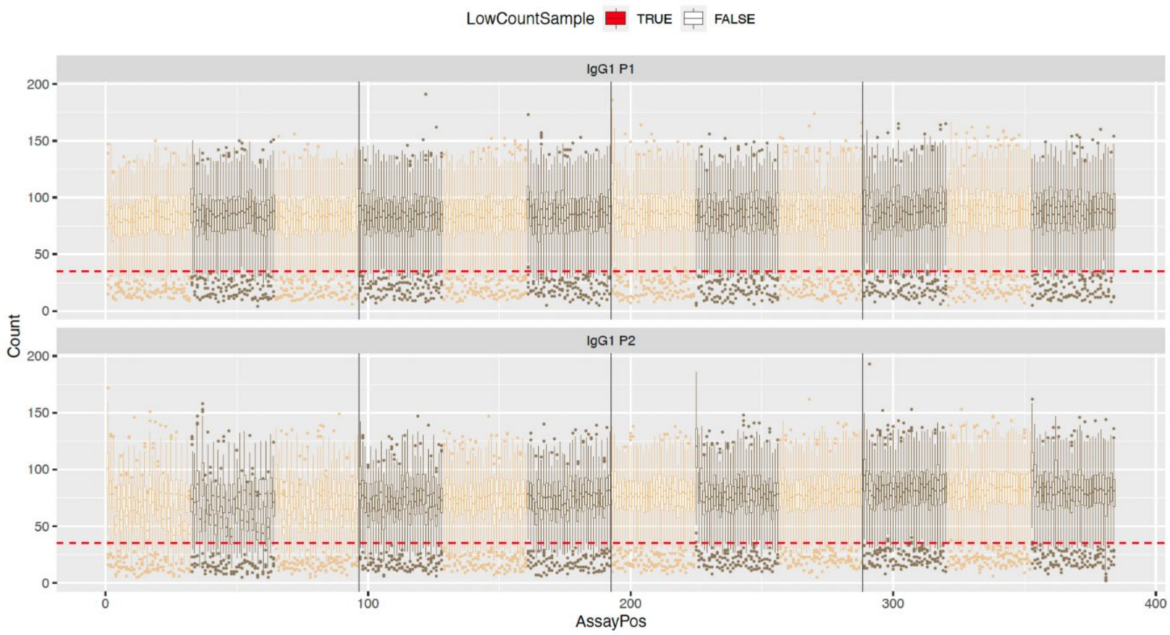

IgG1: Bead count per bead ID

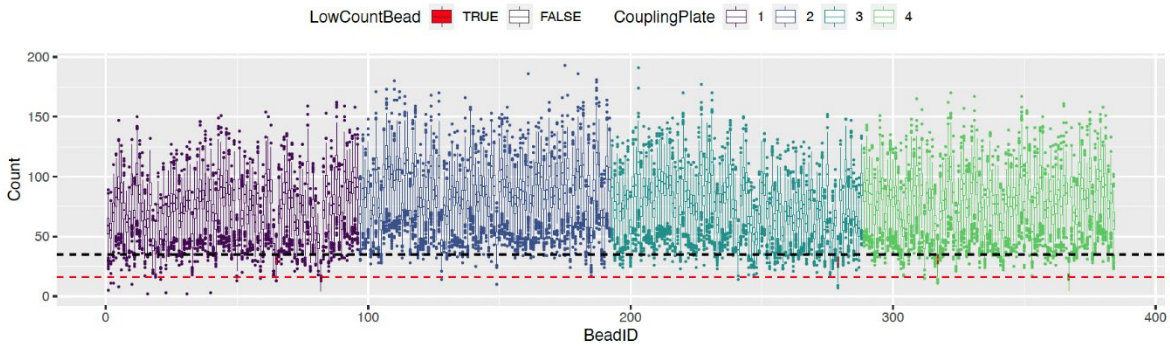

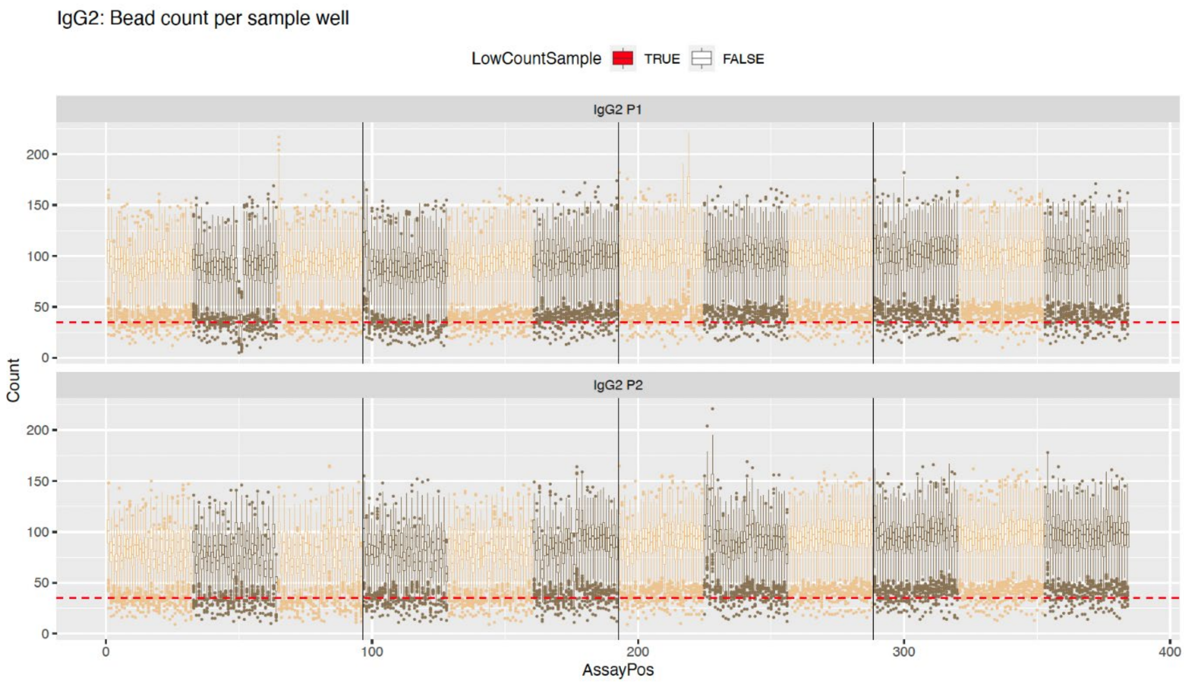

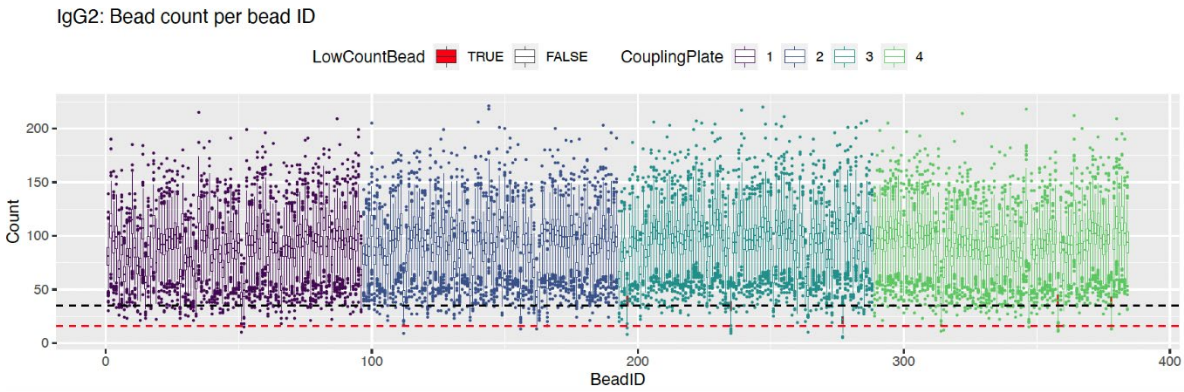

Due to an instrument issue, too few beads of the IgA specific SBA were measured in one of the samples. In this QC filtering, any well with a mean count below 35 is filtered, and subsequently, any bead ID with more than 1 well with a bead count below 16 is filtered. The count QC here allows for one sample with low count for each antigen since it's mainly to separate samples (flagged) that have low count for a few antigens. If quality is prioritized over antigen and/or sample inclusion, a stricter count QC can be applied by excluding these two samples. Both IgG SBAs have many bead IDs with at least one sample below 35 and are therefore flagged (not shown below). However, the data for these are still deemed to be of good quality.

Bead arrays - Quality control – Loading control

One of the bead IDs in each SBA captures general immunoglobulin (subtype specific) and is used as a loading control in the assay. Any sample that results in a signal like a well containing only buffer is filtered.

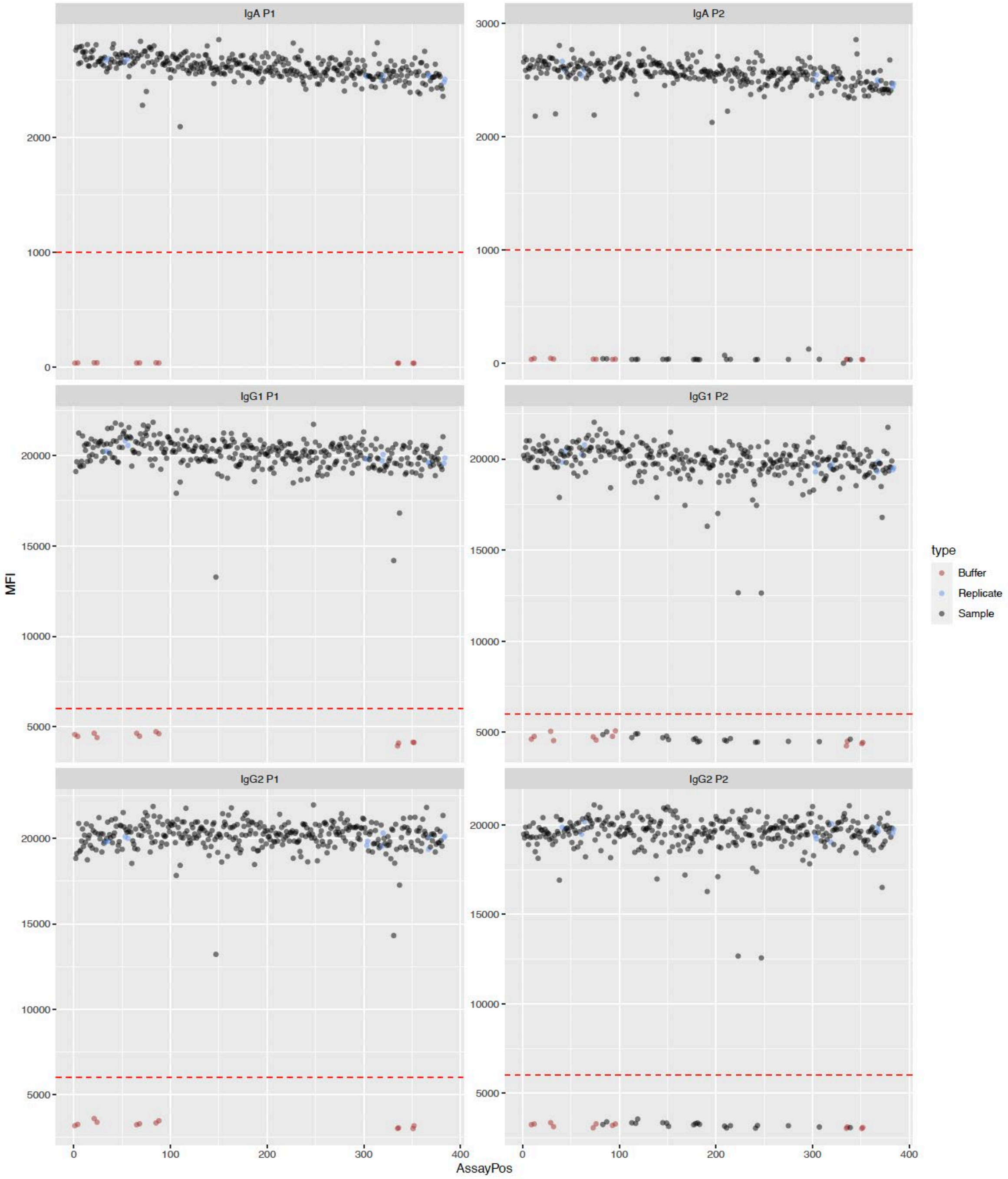

Bead arrays - Quality control – overview

20 samples were filtered based on the loading control using all SBAs, while 2 extra samples were filtered using the IgA SBA. In the raw MFI overview, all data is included, regardless off previous QC steps. These plots are included to give an overall view of the data and potential patterns. The positive technical control beads are excluded from the below sample-wise visualizations to not skew the overall signal distributions.

Samples sorted by analysis order:

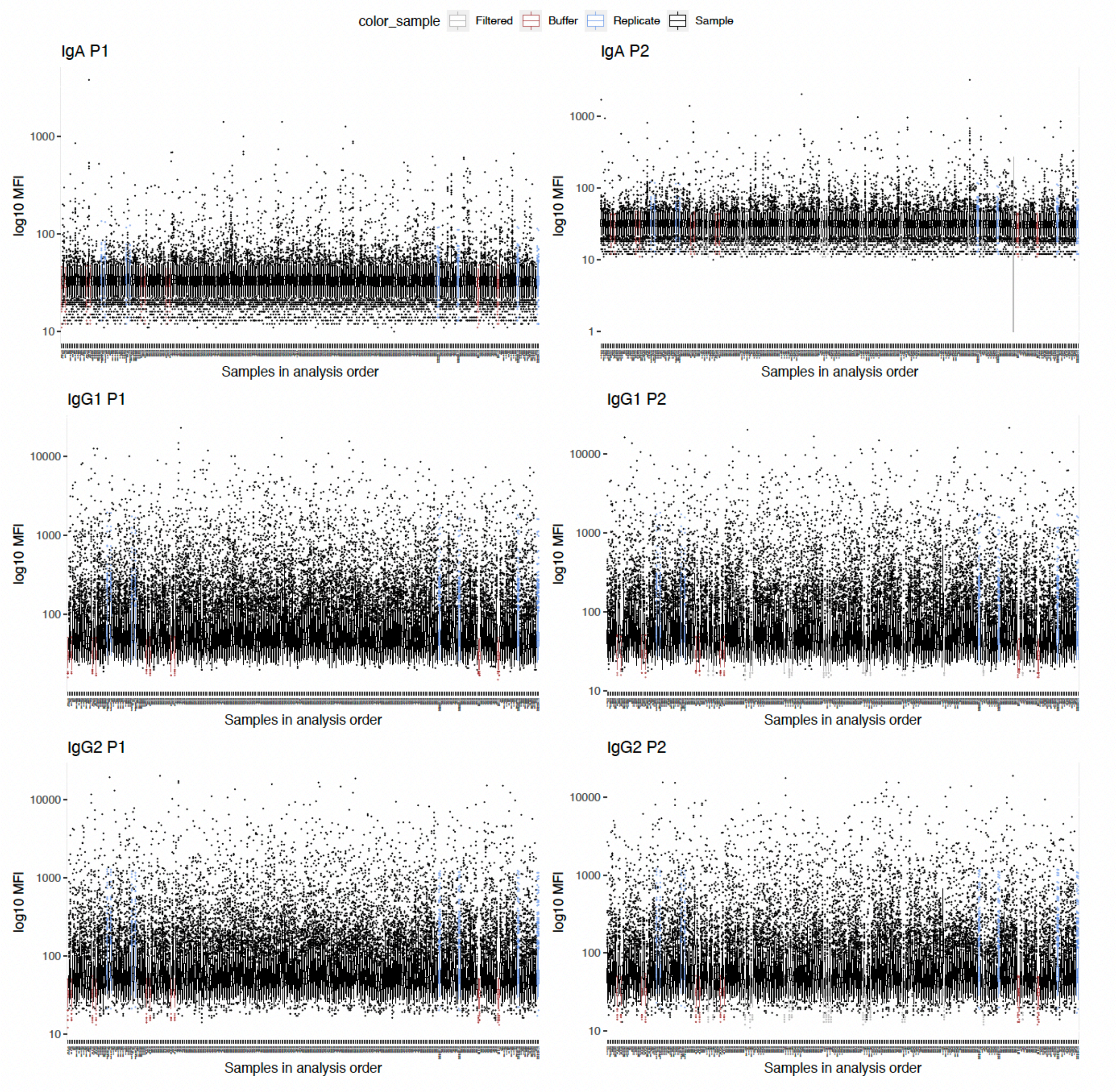

Samples sorted by median:

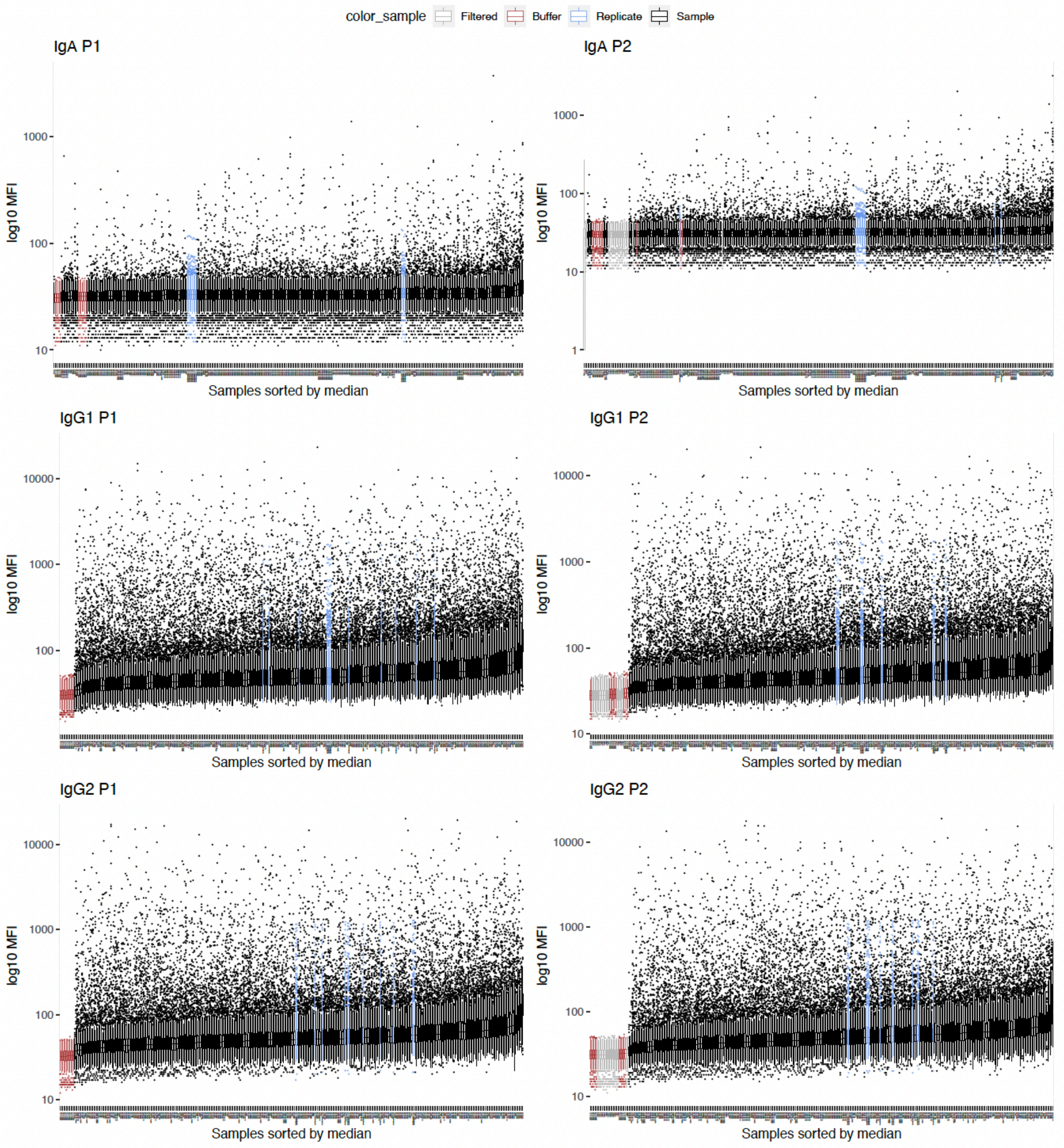

Samples sorted by max:

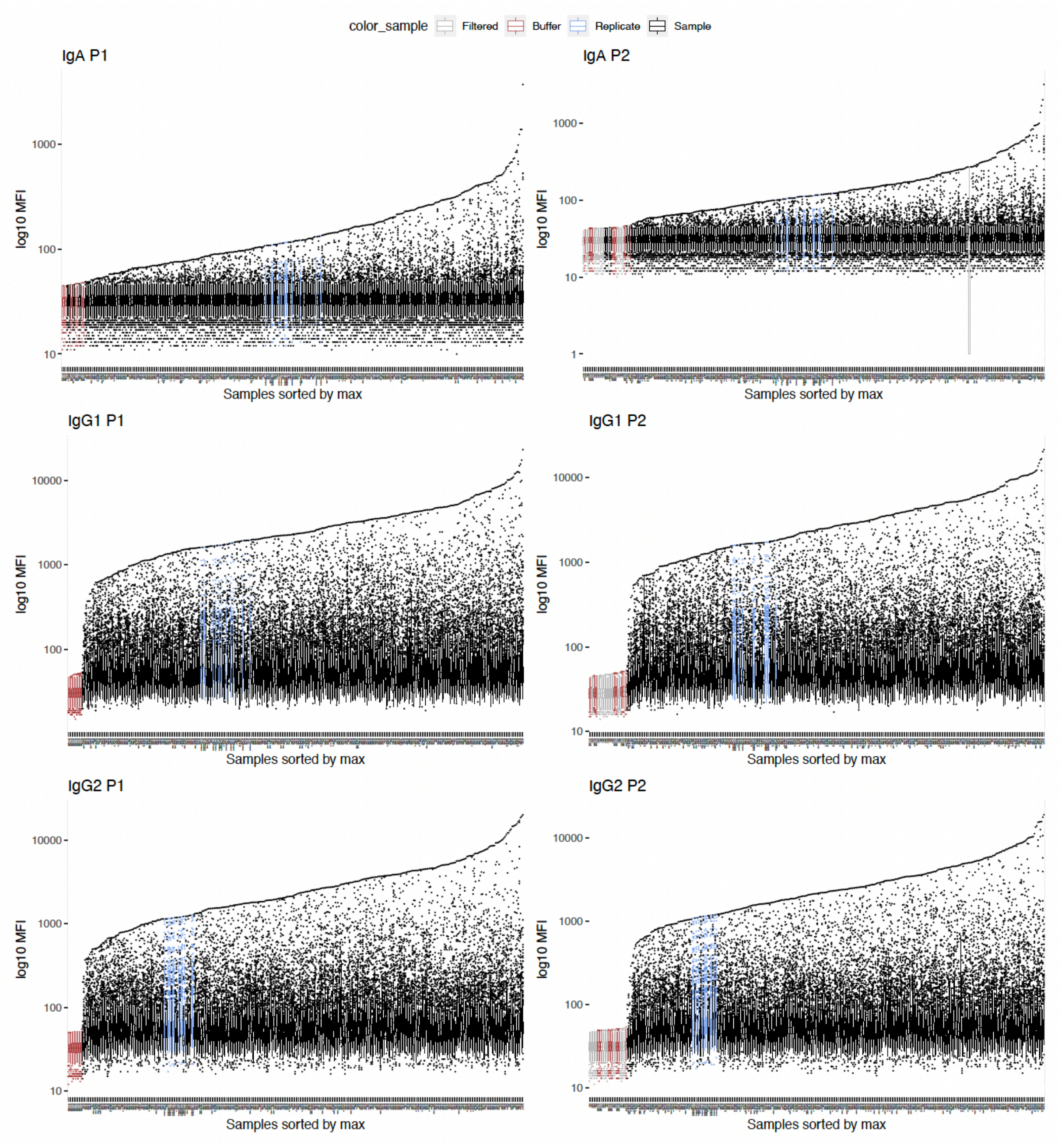

Bead arrays - Quality control – Antigens

Antigens sorted by bead ID order:

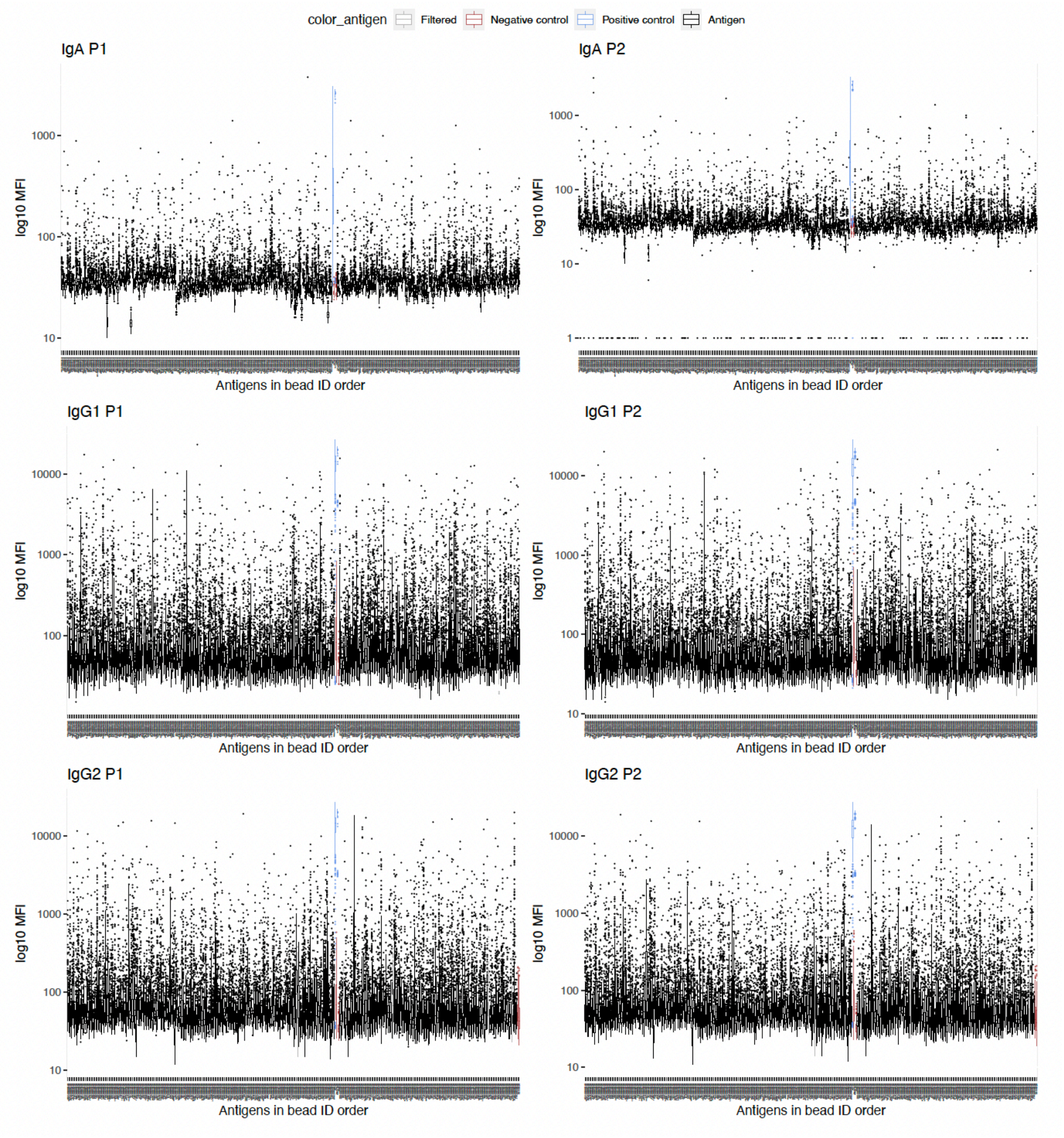

Antigens sorted by median:

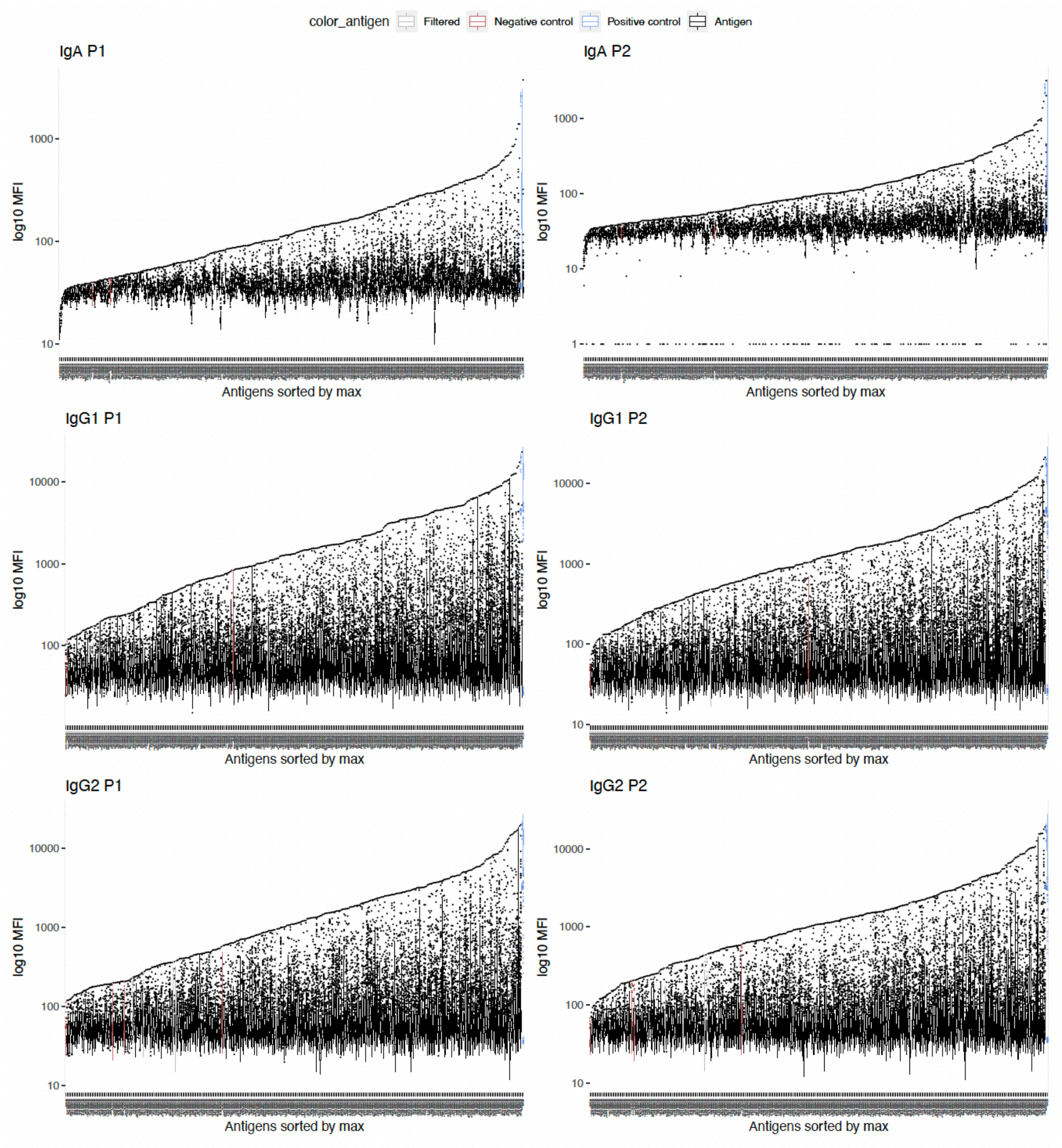

Antigens sorted by max:

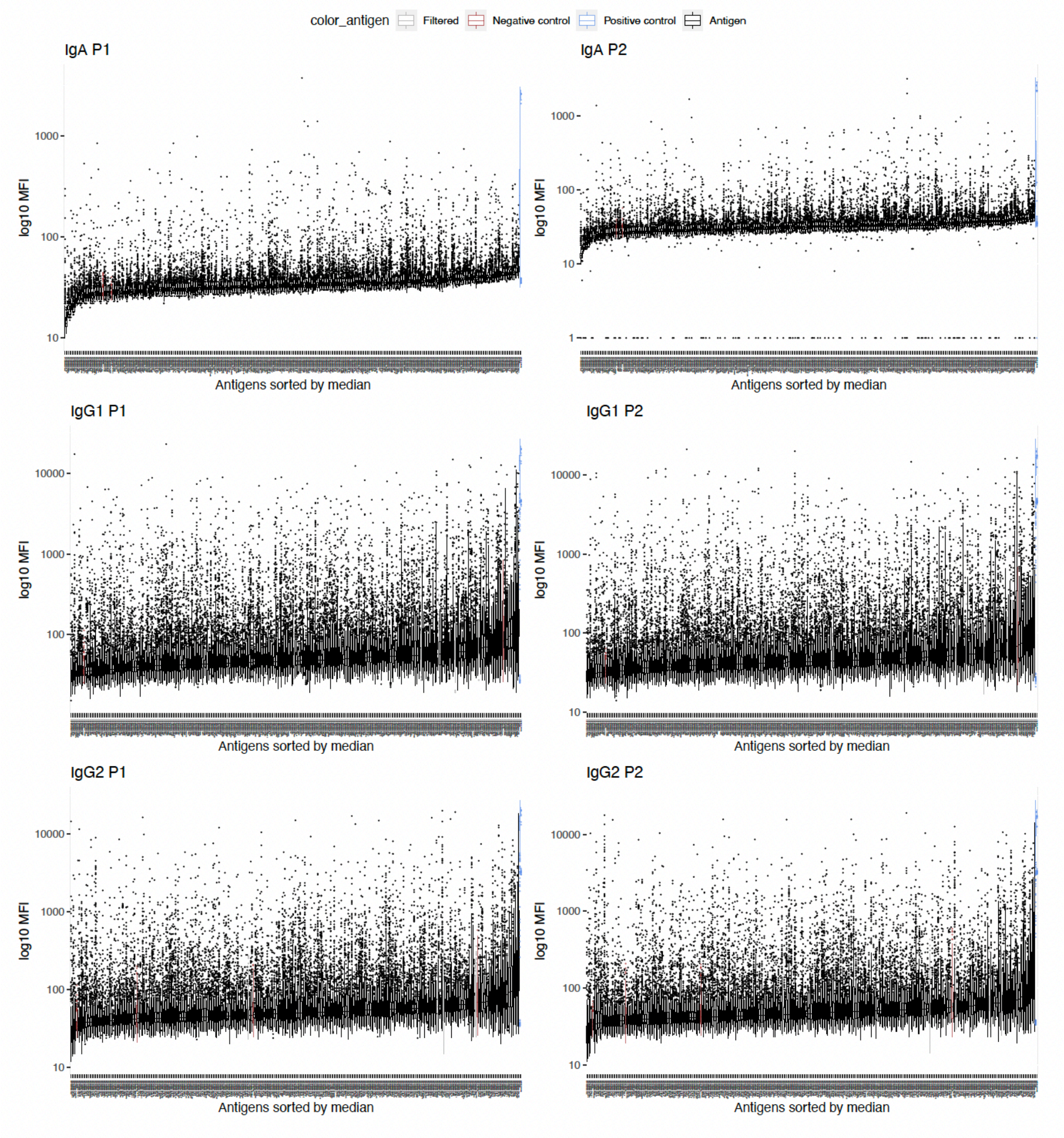

Bead arrays - Quality control – Batch effects raw data

The below UMAPs are based on raw MFI after removing samples and antigens that were filtered in previous steps. The two assay plates overlap nicely for each SBA and the replicates form a cluster (together with some additional samples) regardless of

assay plate, although slightly better for the IgG SBAs as compared to the IgA SBA. The buffer wells do not form a distinct cluster but are relatively close to each other.

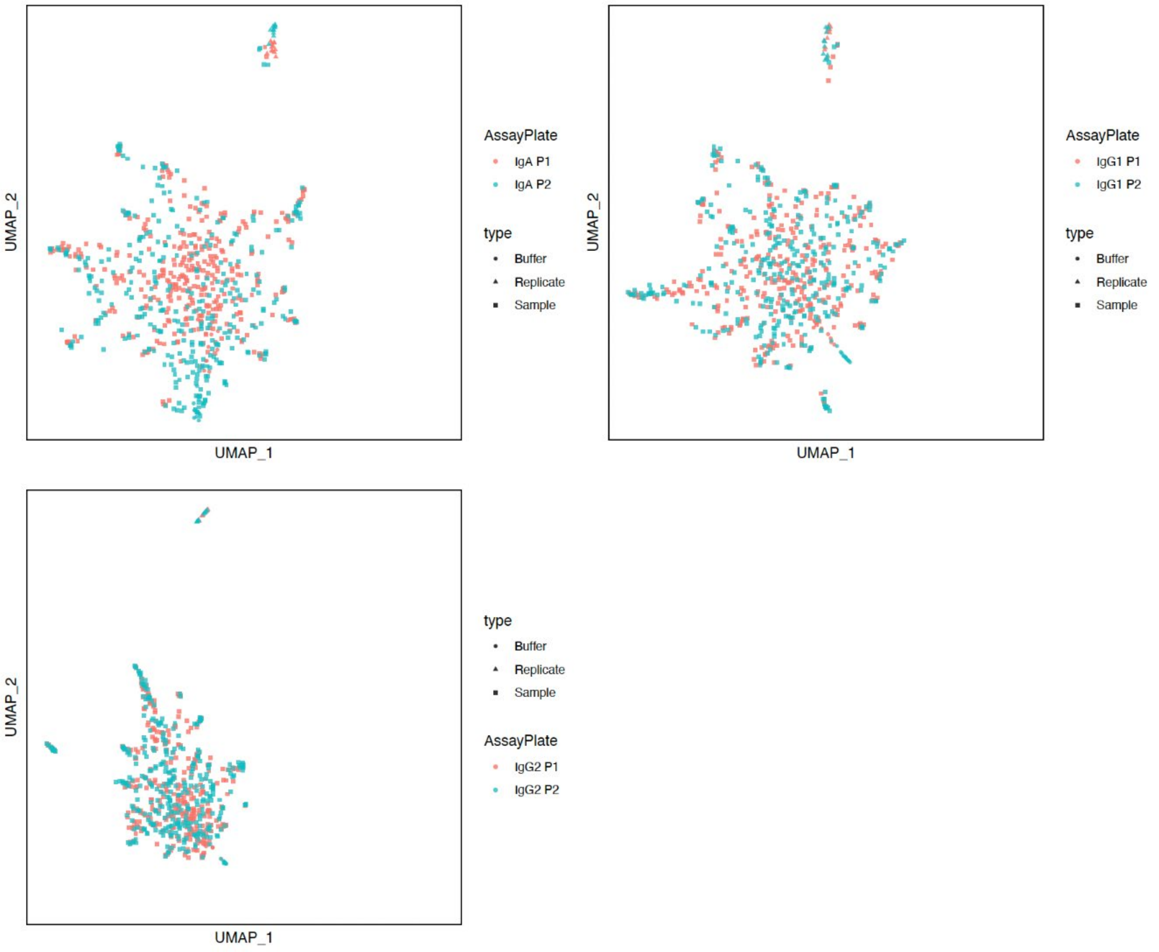

Bead arrays - Quality control – Antigen specific background

Assuming that 10 percent of the samples are non-reactive to any given antigen (but not necessarily that the rest are reactive), the 10th percentile of each antigen (excluding positive control beads) is adjusted to the 10th percentile of all data (excluding filtered wells, control wells, and positive control beads).

Bead arrays - Antigen percentile per swarm (raw MFI)

Dashed lines correspond to the 10th percentile for the antigen, only an example set of the antigens are shown, focusing on antigens with the lowest and highest values at the selected percentile.

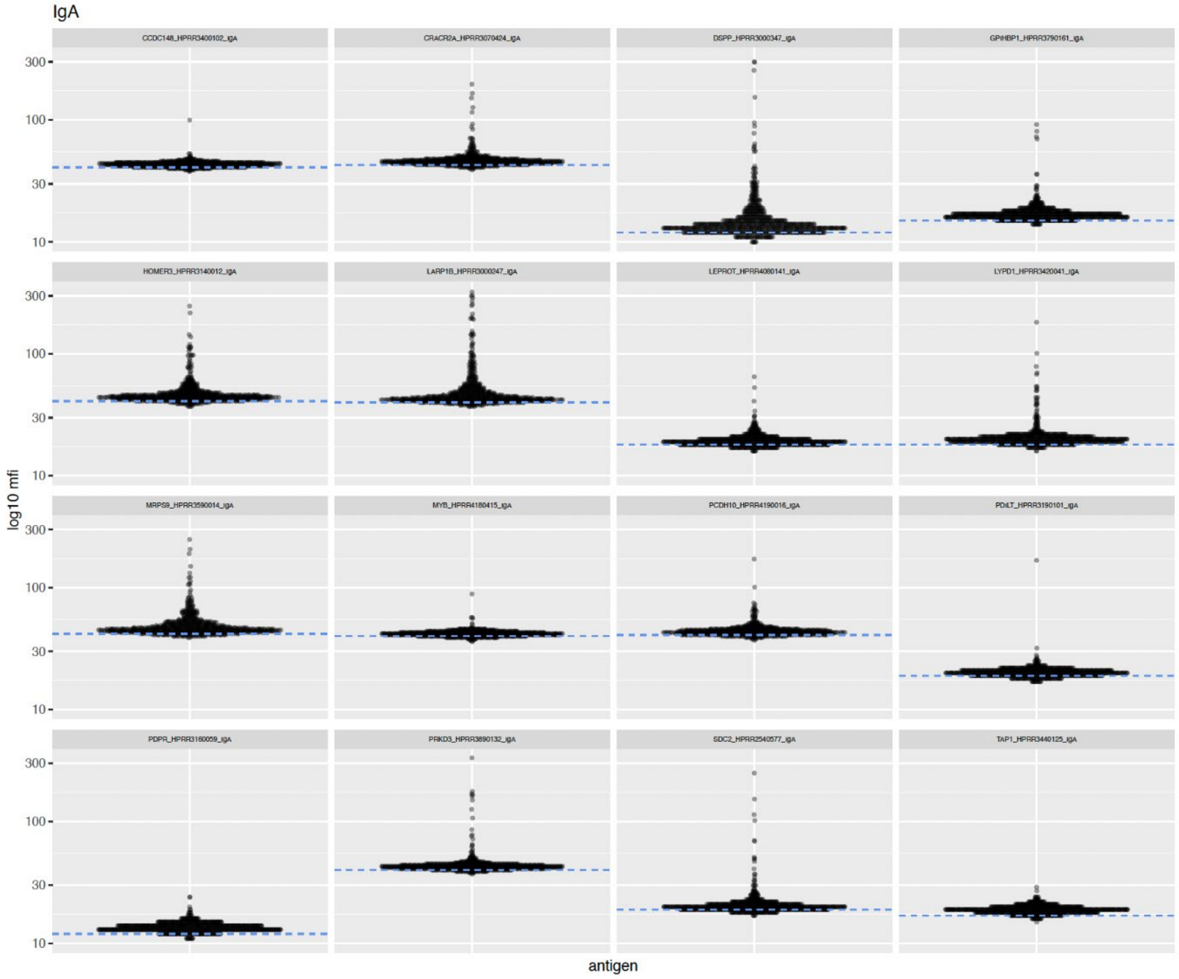

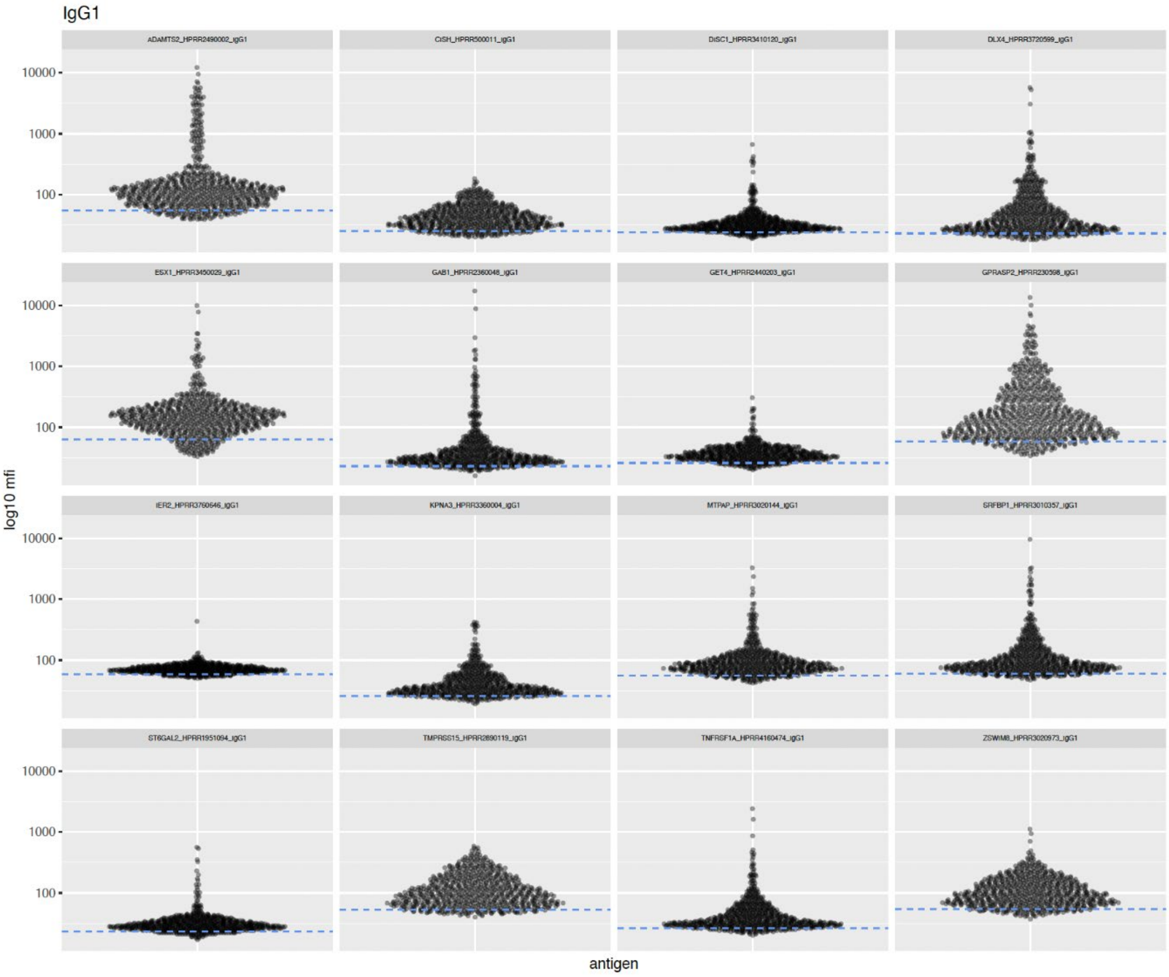
IgG1
ADAMTS2_HPRR2490002_IgG1
CISH_HPRR500011_IgG1
DISC1_HPRR3410120_IgG1
DLX4_HPRR3720599_IgG1
ESX1_HPRR3450029_IgG1
GAB1_HPRR2360048_IgG1
GET4_HPRR2440203_IgG1
GPRASP2_HPRR230598_IgG1
IER2_HPRR3760646_IgG1
KPNA3_HPRR3360004_IgG1
MTPAP_HPRR3020144_IgG1
SRFBP1_HPRR3010357_IgG1
ST6GAL2_HPRR1951094_IgG1
TMPRSS15_HPRR2890119_IgG1
TNFRSF1A_HPRR4160474_IgG1
ZSWIM8_HPRR3020973_IgG1
10000
1000
100
log10 mfi
antigen

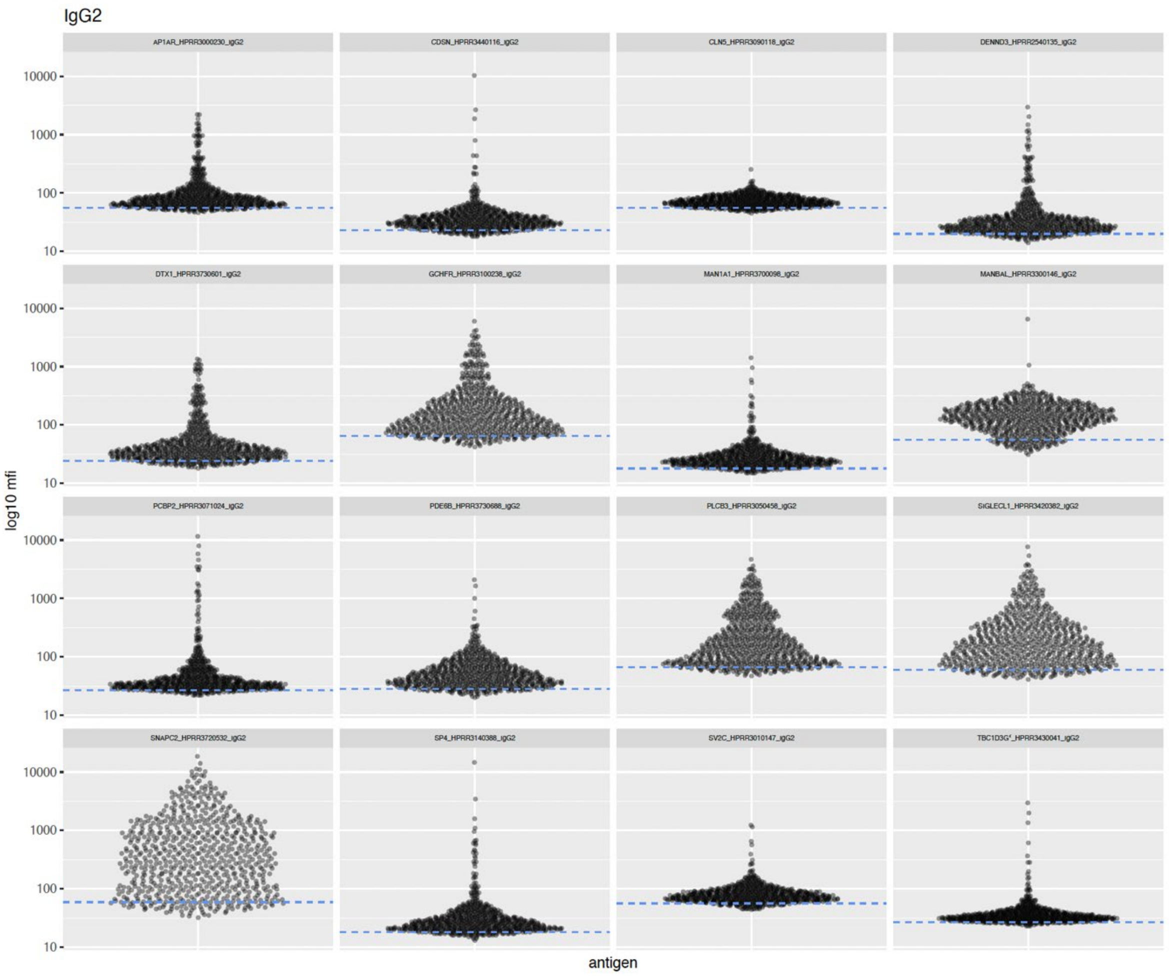

Bead arrays - Quality control – Sample specific background

Samples tend to have slightly different background levels, which can influence the downstream analysis if comparing sample signals within antigens. Below is an example of the varying background levels for ten samples (the five with lowest and highest mean MFI, respectively) across all antigens with a max signal below 100 MFI for the IgA beadstock and 500 MFI for the IgG beadstocks within the ten samples (to focus on the background in the example). Each line is a sample. To even out these differences a sample wise normalization is performed. Note though that the normalization is based on the assumption that a majority of the antigens are non-reactive, i.e. that the median lies in the background and the median absolute deviation

(MAD) is mainly affected by background fluctuations rather than true signals. For downstream analysis, we used in this study the MFIorg (raw MFI).

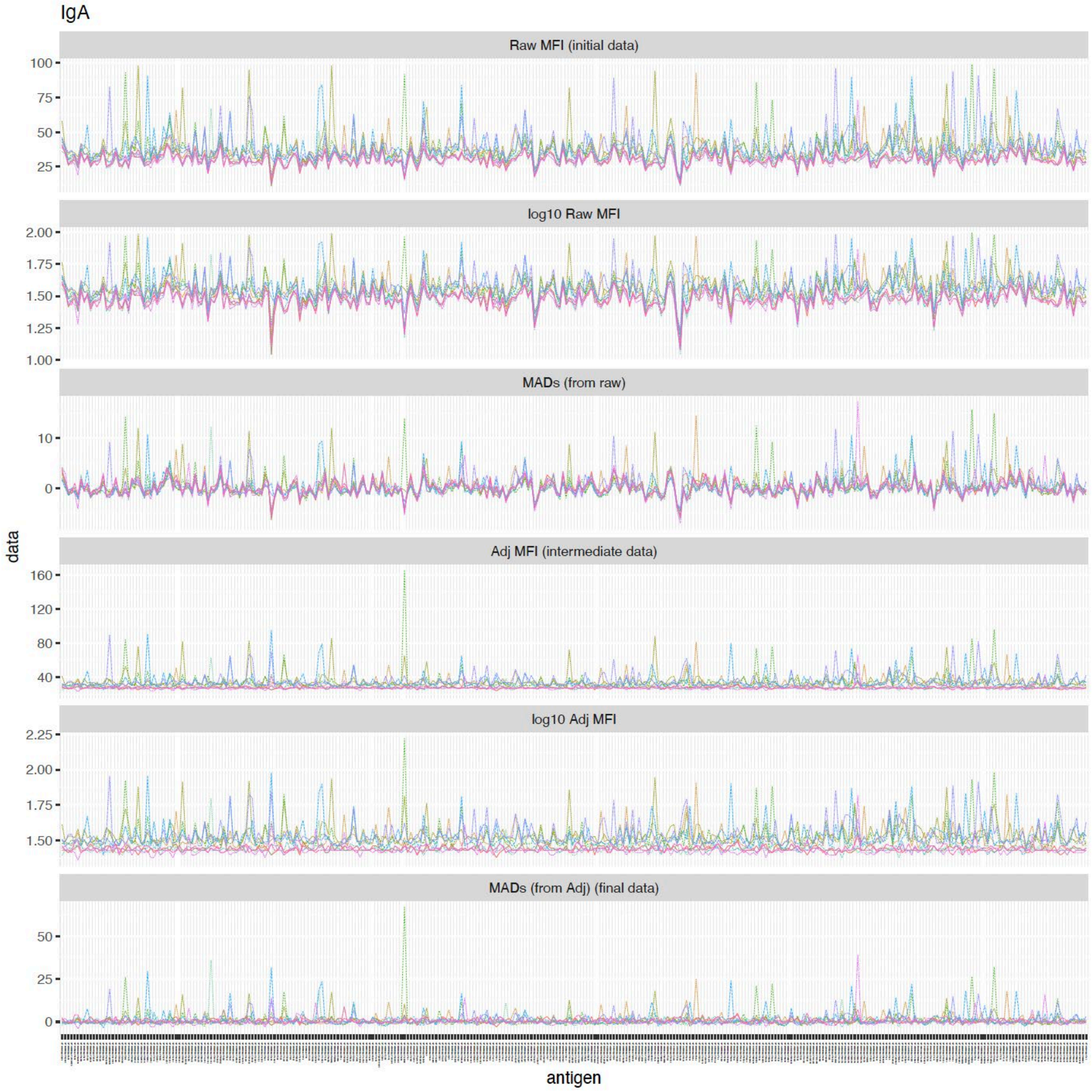

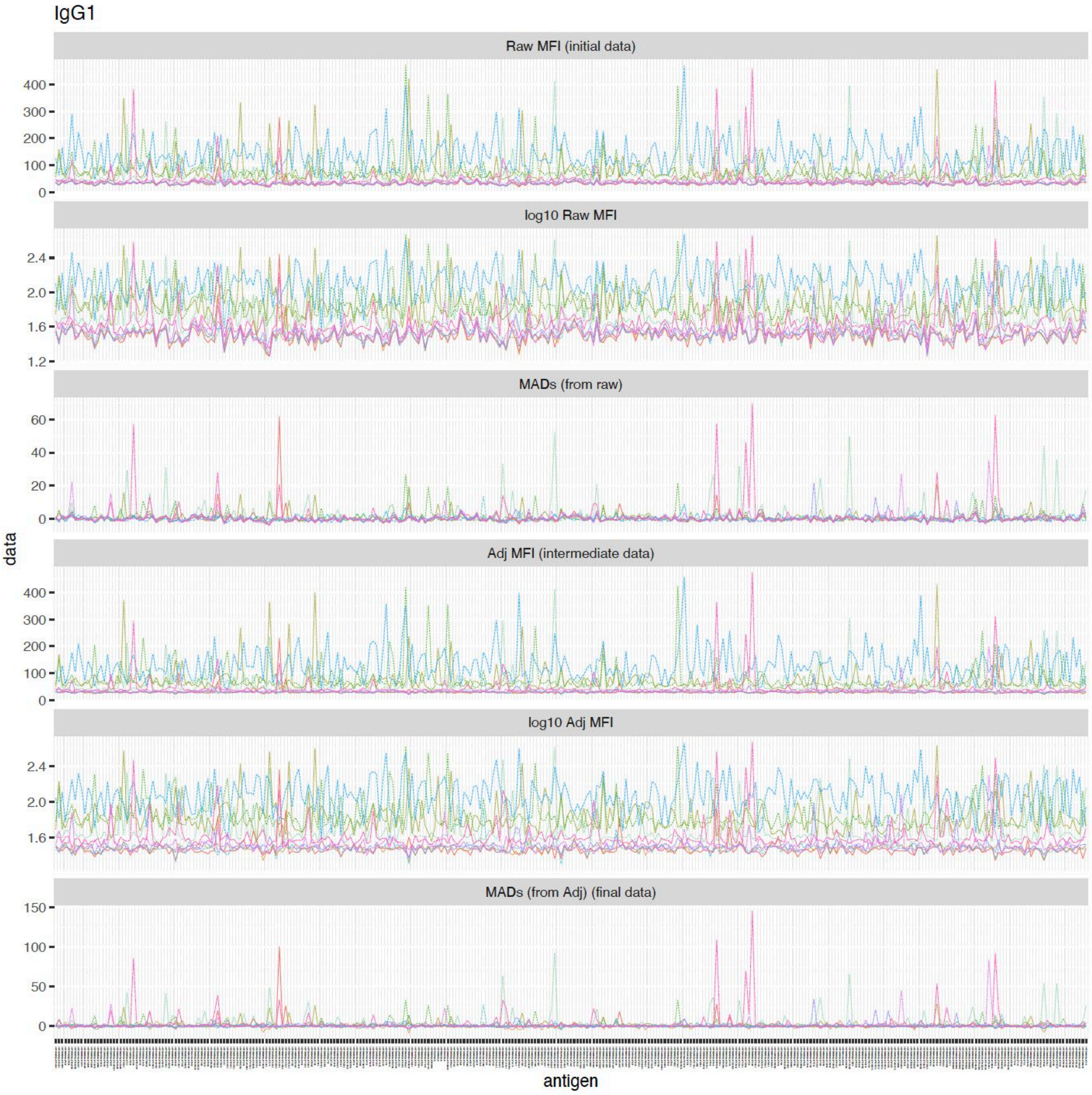
IgG1
Raw MFI (initial data)
log10 Raw MFI
MADs (from raw)
Adj MFI (intermediate data)
log10 Adj MFI
MADs (from Adj) (final data)
data
antigen
400
300
200
100
0
2.4
2.0
1.6
1.2
60
40
20
150
50

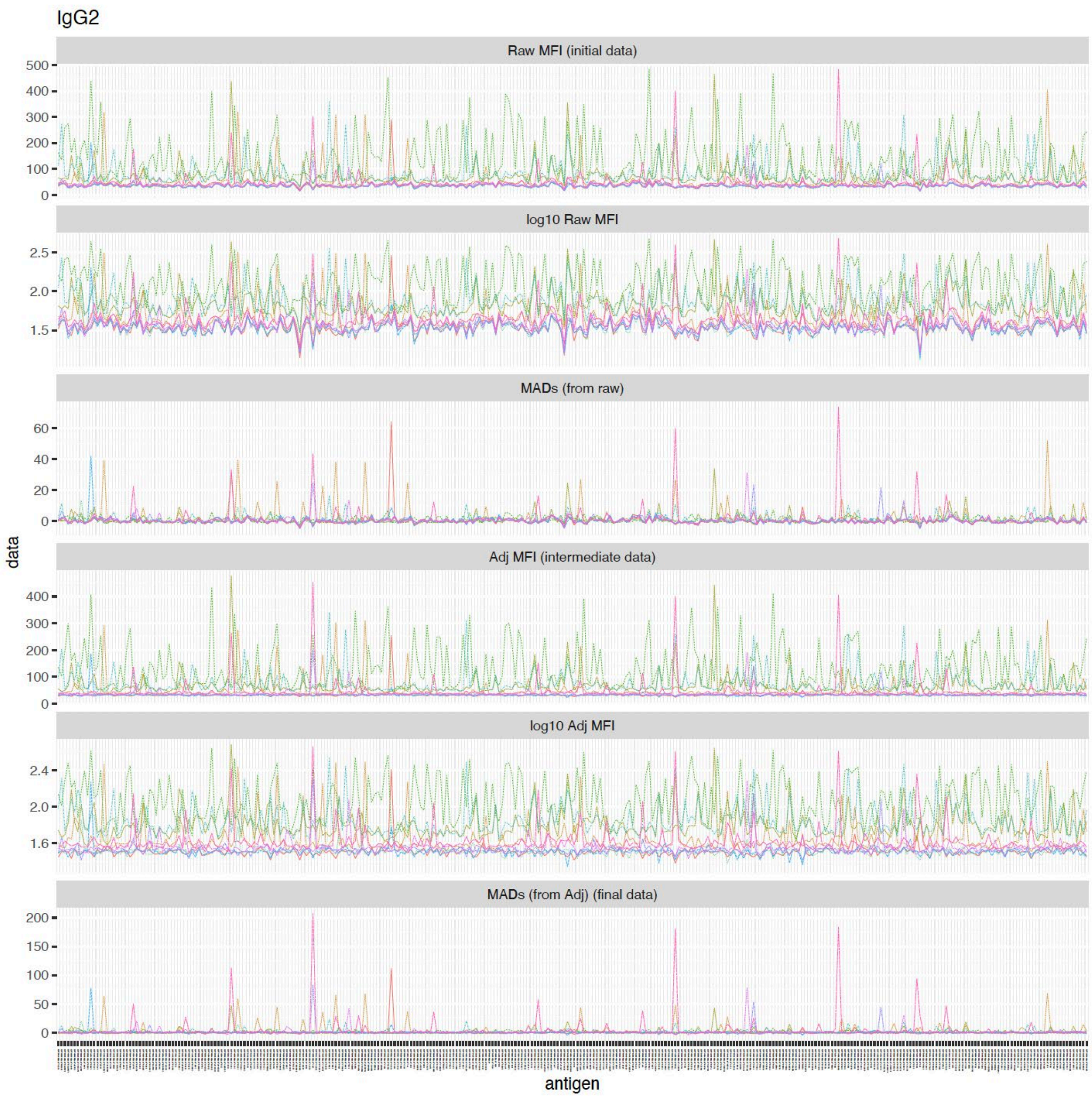

Bead arrays - Quality control – Replicates

Triplicates are included in each sample plate to get a measure of the intra- and inter-plate variation. Since the effect on variance may differ depending on if there are antibodies present (signals above background) or not (background signals), and the signal profile is sample specific, no antigens are filtered or flagged based on this. The signal profiles for the replicates overlap nicely for antigens with a signal over the background, suggesting that the overall reproducibility is good. Each replicate is

represented by a line and color. Each triplicate has the same line type. Since the MADs data is centered around 0 and the background has a narrow dynamic range around 0, CVs calculated on these values would be inflated. Therefore, the MADs values have here been adjusted up by adding the mean of the antigen adjusted MFI values. This makes the three data types have more similar dynamic ranges and thereby makes the CVs more comparable. Most antigens have a CV below 10 %, especially when looking at the inter-assay CVs. Antigens with a CV above 15 % are written out in the figures (although it is not very informative for IgG).

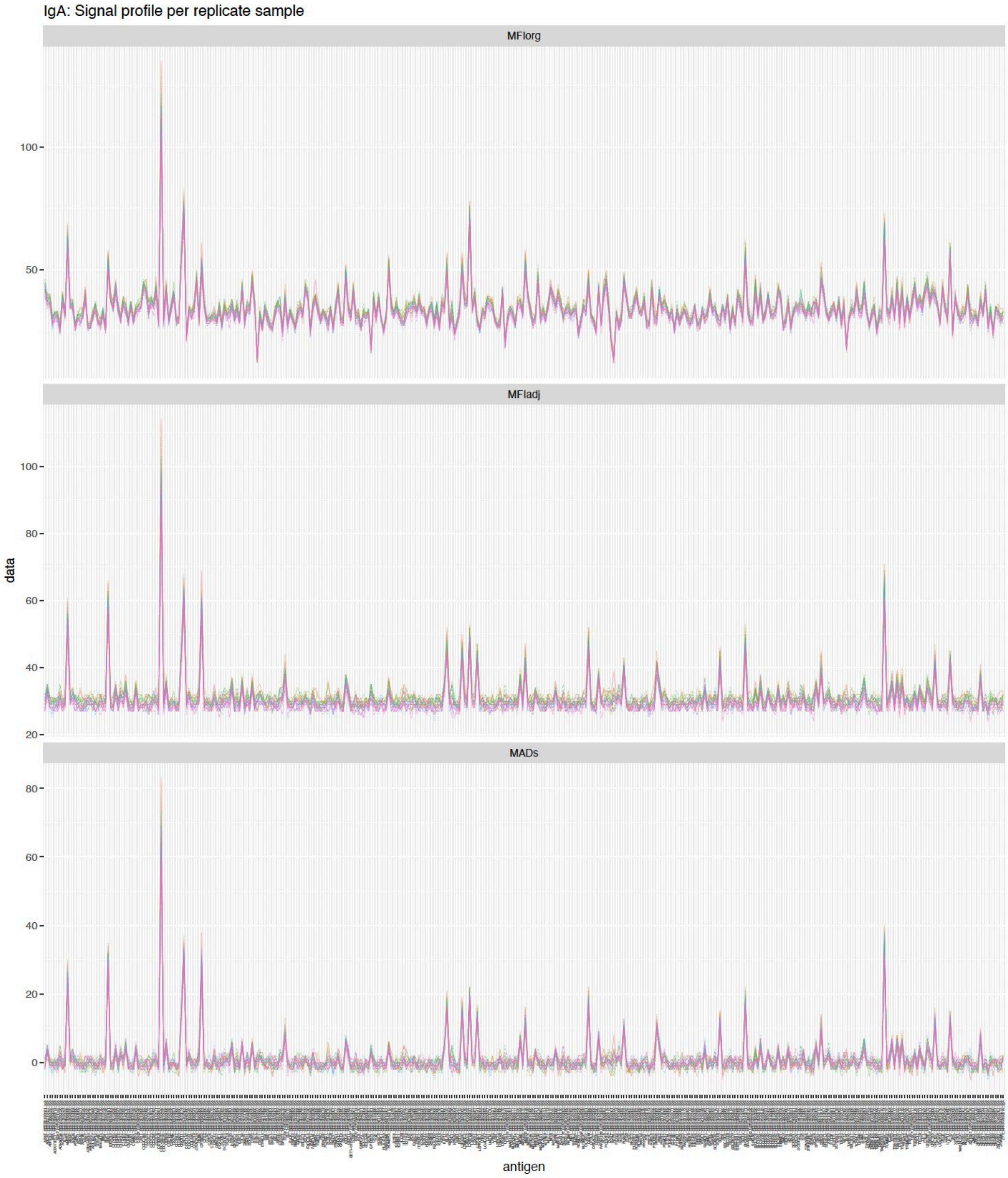
IgA: Signal profile per replicate sample
MFIorg
100
50
MFIadj
100
80
60
40
20
data
MADs
80
60
40
20
0
antigen

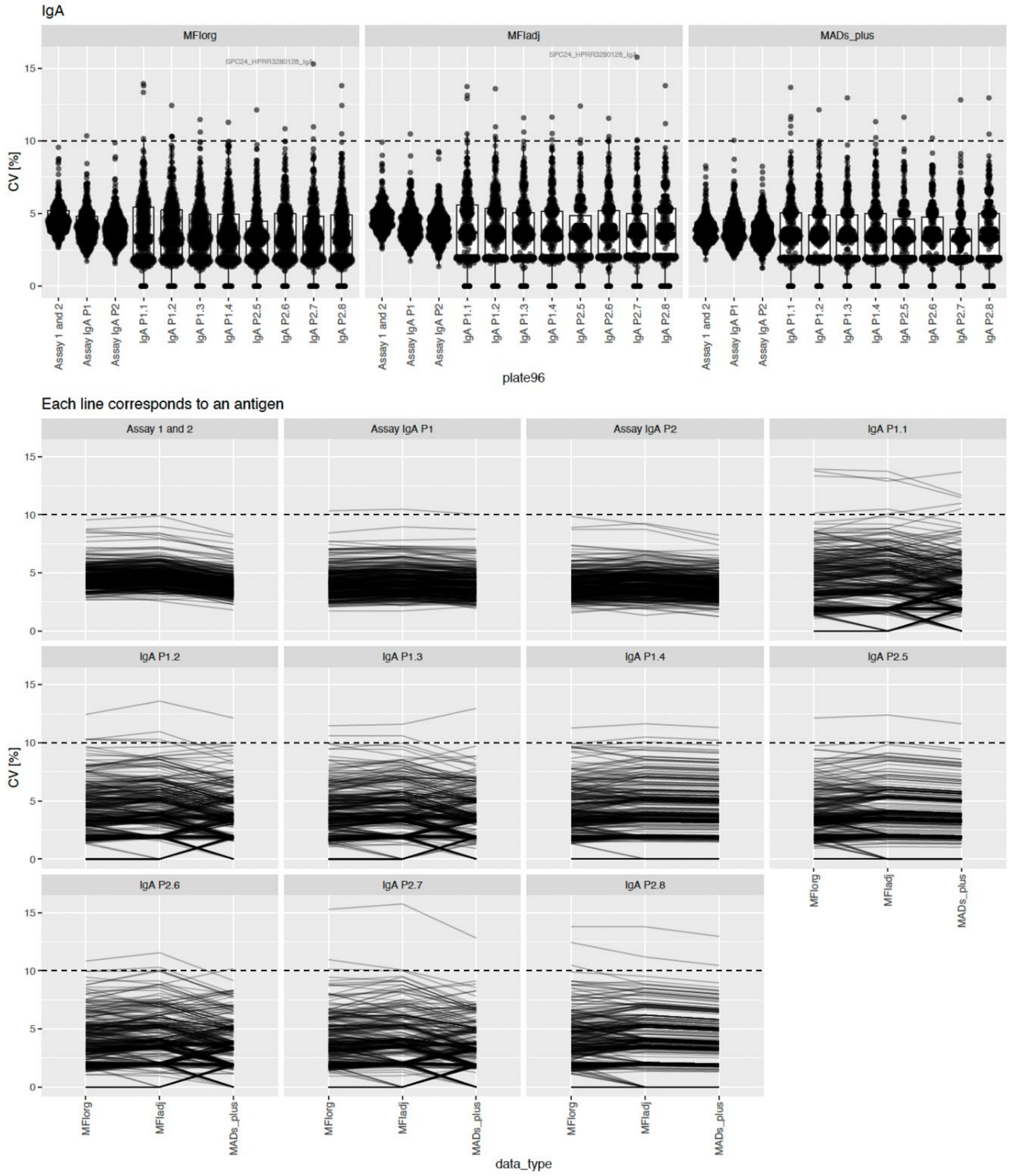
IgA
MFIorg
MFIadj
MADs_plus
SPC24_HPRR3280126_IgA
CV [%]
Assay 1 and 2
Assay IgA P1
Assay IgA P2
IgA P1.1
IgA P1.2
IgA P1.3
IgA P1.4
IgA P2.5
IgA P2.6
IgA P2.7
IgA P2.8
plate96
Each line corresponds to an antigen
data_type

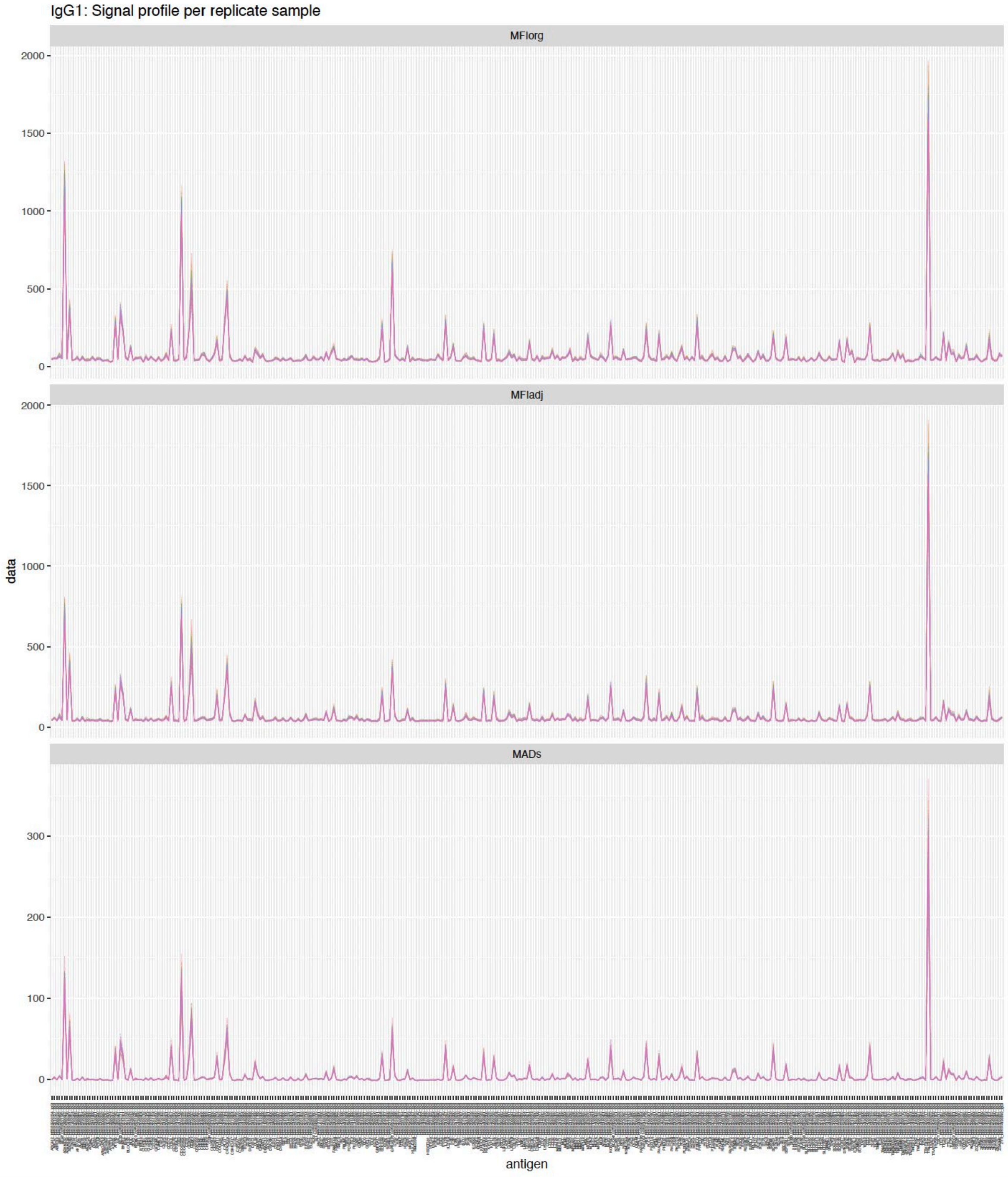
IgG1: Signal profile per replicate sample
MFIorg
MFIadj
MADs
data
antigen
2000
1500
1000
500
0
300
200
100

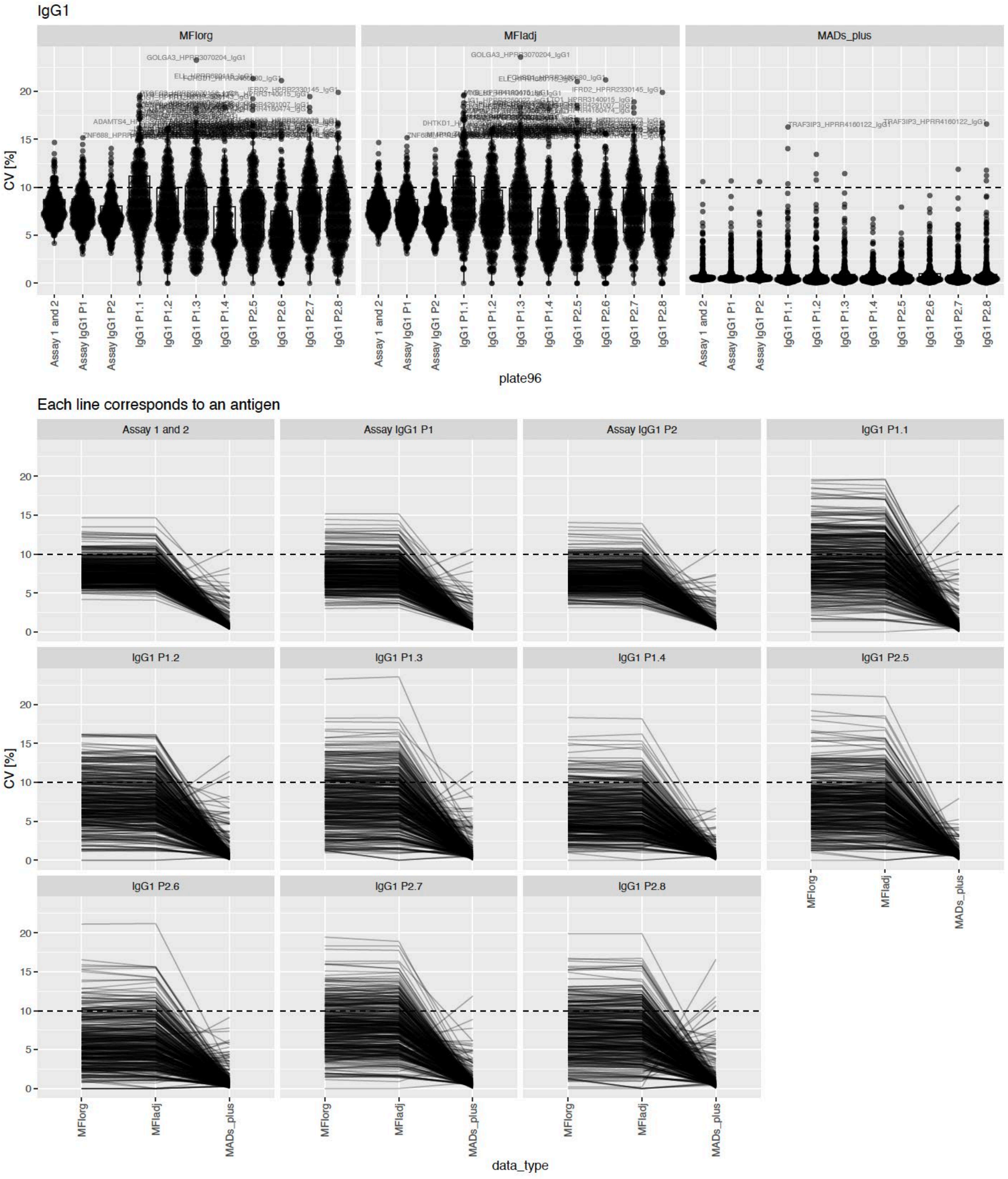
IgG1
MFIorg
MFIadj
MADs_plus
CV [%]
plate96
Assay 1 and 2
Assay IgG1 P1
Assay IgG1 P2
IgG1 P1.1
IgG1 P1.2
IgG1 P1.3
IgG1 P1.4
IgG1 P2.5
IgG1 P2.6
IgG1 P2.7
IgG1 P2.8
GOLGA3_HPRR3070204_IgG1
IFRD2_HPRR2330145_IgG1
TRAF3IP3_HPRR4160122_IgG1
Each line corresponds to an antigen
data_type

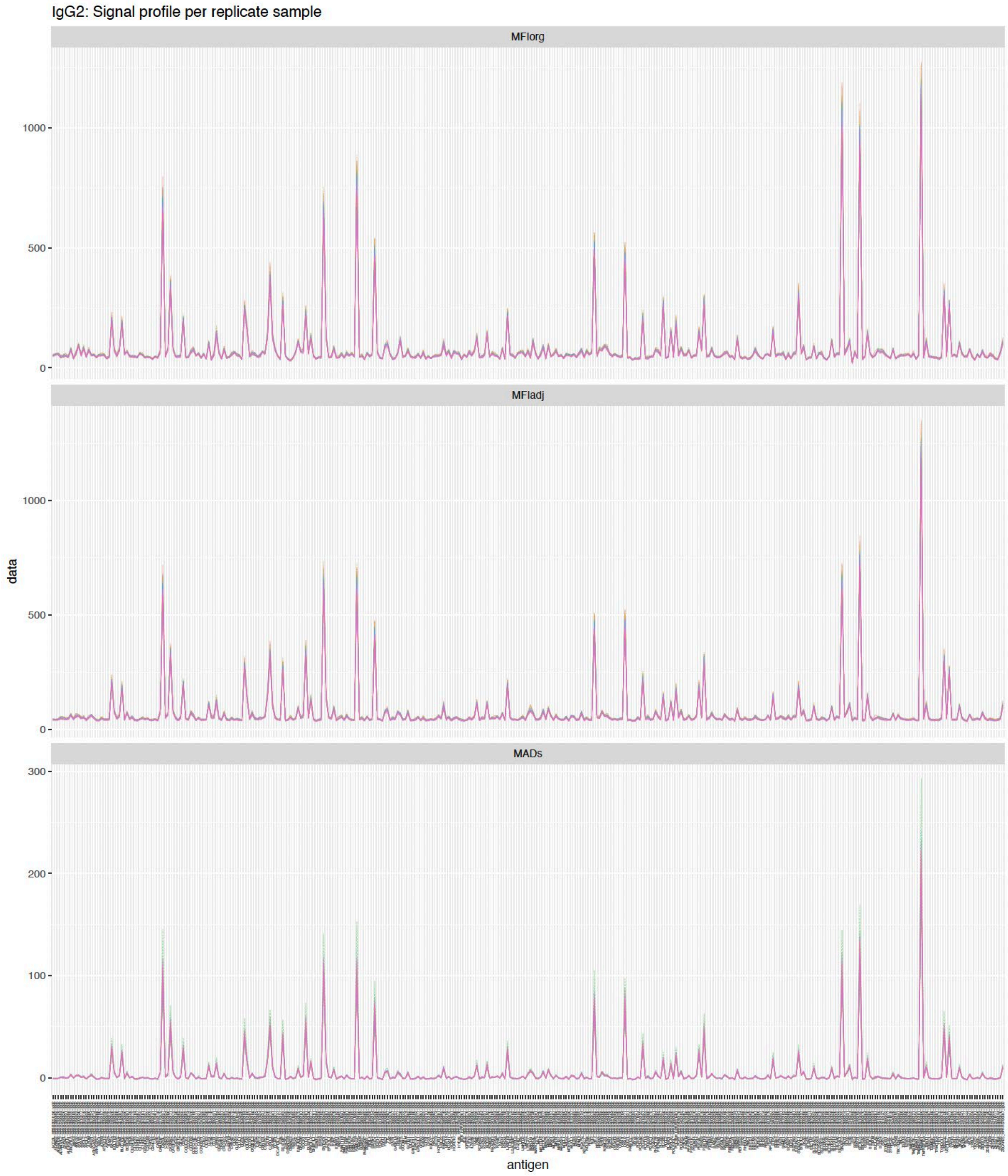

IgG2: Signal profile per replicate sample
MFIorg
MFIadj
MADs
data
antigen
1000
500
0
1000
500
0
300
200
100
0

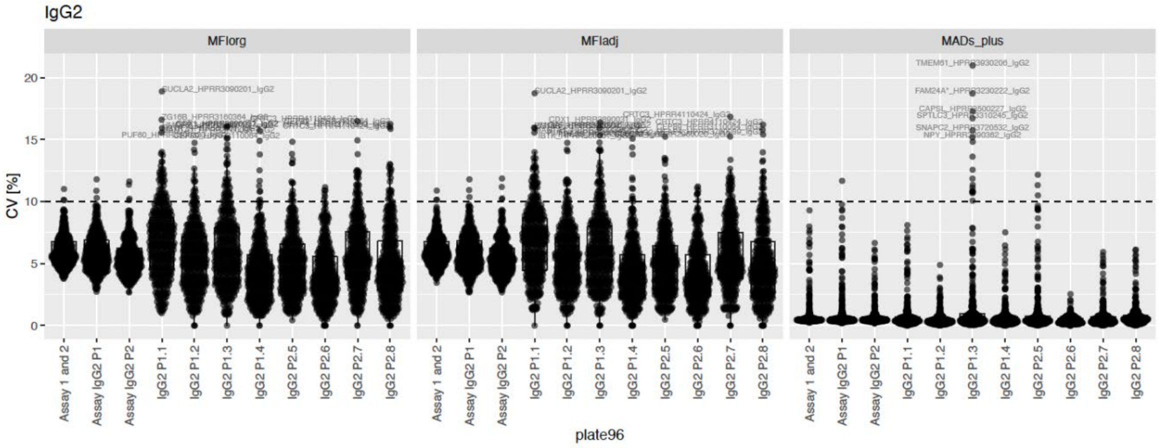
IgG2
MFlorg
MFladj
MADs_plus
CV [%]
plate96
Assay 1 and 2
Assay IgG2 P1
Assay IgG2 P2
IgG2 P1.1
IgG2 P1.2
IgG2 P1.3
IgG2 P1.4
IgG2 P2.5
IgG2 P2.6
IgG2 P2.7
IgG2 P2.8

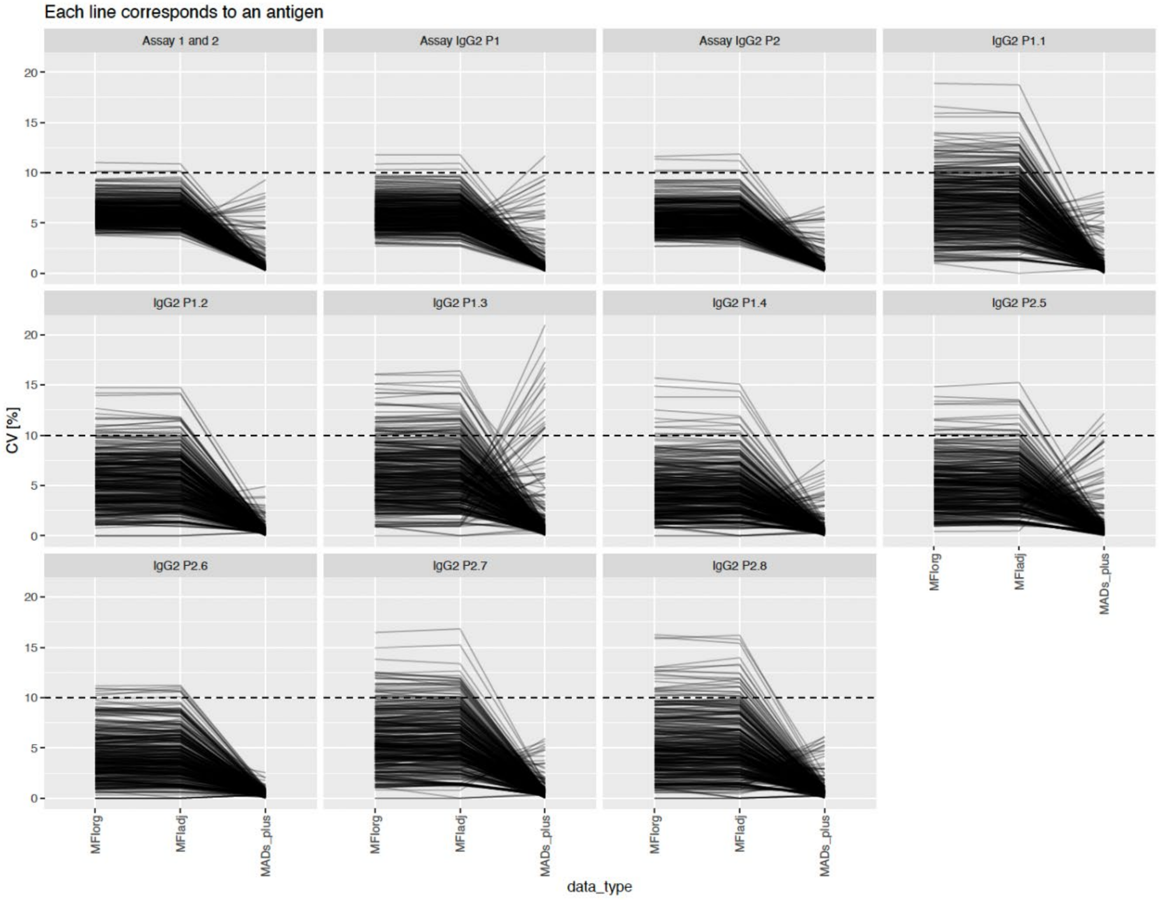
Each line corresponds to an antigen
Assay 1 and 2
Assay IgG2 P1
Assay IgG2 P2
IgG2 P1.1
IgG2 P1.2
IgG2 P1.3
IgG2 P1.4
IgG2 P2.5
IgG2 P2.6
IgG2 P2.7
IgG2 P2.8
CV [%]
MFlorg
MFladj
MADs_plus
data_type

Patients

Sera were analyzed at baseline for most individuals (n=405) and at the timepoint closest to any severe event (liver transplantation, hepatobiliary cancer, or death) in 47. Deviation from the selection criteria was noticed retrospectively as one patient from the "progressor" group had received Humira (Adalimumab) before sampling. The rational for the selection and grouping of PSC patients was based on the hypothesis that unknown autoantibody could drive PSC phenotypes. Groups were designed to mainly differ by one single feature that could be explained by the presence of an autoantibody such as the presence of IBD or of advanced stage of the disease. Furthermore, matching at the group level was performed for several criteria that could potentially induce bias (such as total IgG and IgA levels, sex, age). In each group, we aimed to reach a balance between representativity and heterogeneity. For example, various types of IBD were included to reflect the clinical setting, and both advanced and early disease stage were included within the non-IBD or the CCA groups. The size of the groups also aimed to find a balance between pools containing too few individuals (with the risk of being not representative and missing detection autoantibodies with low prevalence), and too many individuals (with the risk of diluting below the detection limit of autoantibodies with low prevalence). We estimated that groups of six to eight individuals could allow a relatively good representativity of each phenotype while allowing detection of autoantibodies with prevalence as low as 12,5% (if at least one out of eight patients would have it). A total of 33 patients were included on five arrays which we considered as a reasonable number of individuals and replicates to identify autoantibodies in PSC. Small duct PSC was diagnosed in cases with clinical findings of PSC (cholestasis without any other cause), presence of IBD, and a biopsy with typical findings of PSC. AIH overlap diagnosis was set in cases with typical features of AIH in addition to PSC cholangiographic changes (increased transaminases and total IgG and a biopsy showing interface hepatitis5. Cirrhosis diagnosis was set in cases with clinical or radiological signs of cirrhosis, presence of portal hypertension and/or elastography measure >20kPa or biopsy (F4). PSC patients were considered having an active

IBD when requiring intensified treatment (increased doses of 5-ASA, a course of steroids or introduction of azathioprine or biological treatment). Sera from healthy donors (n=91), individuals with other liver diseases (n=62) and indiviuals with IBD (n=62) served as control samples. IBD controls were matched at a group level for age at IBD diagnosis, subtype of IBD and colectomy. Healthy donor controls were matched for sex and age at time of sampling. Liver disease controls comprised individuals with AIH (n=15), PBC (n=13), cirrhosis (alcoholic, metabolic or cryptogenic) (n=14), non-PSC-CCA (n=9), and non-PSC-cholangiopathy in need of ERCP (n=10). Hepatitis B and C were exclusion criteria in the first phase of the study as well as in the second phase for the controls. Although this was not an exclusion criterion for the PSC patients in the second phase, only one patient was positive for PCR HCV-RNA at inclusion (2014) but eradicated after treatment and considered cured (2016). Ethical approval was acquired from the Regional Ethical Board, Stockholm and Uppsala, Sweden (Dnr 06/245-1, 2011/2-6, 2012/2141-31/1, 2013/188-31/1, 2013/2084-31/1, 2021-05560-02).

Study limitations

The study has some inerrant limitations due to its design and the method used. Specifically, (i) The selection of patients, especially in the first phase might have influenced the results obtained in the second phase. Although 33 individuals into five different planar arrays have been used, we can’t exclude that different results would have been obtained with different patients. (ii) The features of the planar array itself define limits in the datasets produced. Although the antigens included in the assay are generally longer than peptides and may therefore present in a folded structure, we can’t exclude that this structure is not identical to the physiological state on the full-length protein *in vivo*. We also can not detect conformational epitopes potentially given by multi-protein complexes. Finally the proteins fragments do not contain post-translational modifications. (iii) The biological generation and biochemical properties

of the AAB also limits the interpretation of the data. This mainly relates to the antigenic specifity of the immune response detected. We can't exclude cross-reactivity due to oligoclonality, polyclonality and low avidity. Such cross-reactivity could happen between several protein fragments used in the planar or the bead arrays (similar linear or conformational epitopes) but could also be due to any other antigenic response developed during the life of the patients (similar viral, bacterial or food antigens for examples). (iv) The potential bias caused by unknow biological factors. As we used serum and not purified immunoglobulins, we can't exclude that an unkown biological product might interfer in the assays. (v) The selection of the protein fragments. We can't exclude that patients have AABs targeting regions outside the chosen fragments. (vi) The inerrant statistical underpower in proteome profiling. Although the statistic laws do not always apply in biology, we can't exclude that some of the results are false positives. Performing such a large screen with a rare disease is always a challenge especially in the case of PSC that is slow progressing with even rarer severe events such as presence of cancer. In that case with a group size of n=17 (cancers) and a proteome screen of >42,000 AAB we can't exclude that some findings are "statistical false positives". However, as all individuals enrolled in the study were from Sweden, we can't distinguish between a statistical false positive result and a true biologic positive result seen specifically in the Swedish population (for example due to genetic and/or environmental particularities). Altogether these remarks highlight the need of more targeted international replication studies.

Statistical analysis

To detect associations between autoantibodies and severe events, we compared the samples taken close to the events with samples taken at inclusion from patients that did not develop any event during the follow-up. Some patients were reported with multiple events

such as both “transplanted” and “death”, or “HB cancer” and “death”. In those cases, we assigned these patients to the single event that occurred first. “HB cancer” comprised CCA, GBC, HCC, and “death” comprised various causes related to non-hepatobiliary cancers (such as breast or colorectal) or others (such as COVID-19 or stroke). No data was imputed. The numbers of patients used for each analysis are available in the Supplementary table 3. Details on data handling, including normalized steps, quality controls are provided in the Supplementary material and methods document. Within the highlighted clusters in Figure 2, the top significant results were selected with a maximum of six AABs to illustrate the relationship with biological, radiological and clinical parameters in Figure 3. The function ggballoonplot from ggpubr package was used for Figure 2. The clustering was performed using the Ward’s method (function hclust, method ward.D2). ROC curves were mainly used to depict associations, not to establish predictive models to be used in a real-life clinical setting. Statistics of sQTL and eQTL were extracted from GTEX, co-expression analysis done using AnalyseR, diagrams (Chord and Sankey) with Raw graph2.0, odds ratio computed with MedCalc. For AID, p-values were selected based on the most significant associations with thyroid and skin-related AID (the most common in our cohort). For hepatic decompensation, p-values were selected based on the most significant associations with either ascites, variceal bleeding, or encephalopathy at time of sampling. Statistics for each of these parameters individually are provided in the supplementary material. Enrichment patterns were defined according to the Human Protein Atlas classification. Tissue enriched: at least four-fold higher mRNA level in a particular tissue compared to any other tissue; group enriched: at least four-fold higher average mRNA level in a group of 2-5 tissues compared to any other tissue; tissue enhanced: at least four-fold higher mRNA level in a particular tissue compared to the average level in all other tissues; cell type enriched genes: at least four-fold higher expression levels in one cell type as compared with any other analyzed cell type; cell type group enriched genes: enriched expression in a small number of cell types (2 to 10); cell type enhanced genes: only moderately elevated expression. ROC performances were computed based on logistic regression (Prisms 9). Analysis of interactions was not included in the models.

Data availability

Public access to the data is restricted by Swedish Law and prohibit the release of individual-level datasets that could potentially allow a personal identification. Consequently, only summary-level of such data are allowed to be publicly released. In this study, this particularly concerns the clinical metadata and patients' s characteristics analyzed in relation to the autoantibodies. However, data access can be granted in the framework of a data transfers agreement. Anyone wishing to gain access to the data can contact Martin Cornillet and Annika Bergquist (martin.cornillet.jeannin@ki.se, annika.bergquist@ki.se).

Contributions

All authors contributed to the study (technically or intellectually), the review of the manuscript and the interpretation of data. MC and AB designed and financed the study. MC led the data analysis and draft of the manuscript with major contribution of ALB, AB, and DS.

Acknowledgement

We thank Ronald Sjöberg, Margaretha Anggraeni Andersson, and Ceke Hellström, "Scilifelab Autoimmunity and Serology Profiling unit" for constructing the antigen arrays and generating the data. We also thank Linda Nordström for her technical assistance.

Funding

This project received funding from the Swedish Research Council (2020-06250 to MC and 2022-01255 to AB), CIMED (FoUI-9626 71 to MC and FoUI-973336 to AB), Region Stockholm (RS2020-0731 to AB) and The Swedish Cancer Society (23 2665 Pj 01 H to AB).
