# Supplementary material for: Proteome-scale autoantibody profiling in PSC: Associations with clinical phenotypes and evidence for neuroendocrine deregulations
Source: JHEP Rep. 2025 Dec 23;8(3):101719. doi: 10.1016/j.jhepr.2025.101719 (PMC12925457; doi:10.1016/j.jhepr.2025.101719)

MST1 rs3197999

Tissue	Samples	NES	p-value	m-value
Brain - Cerebellum	209	0.427	8.5e-8	1.00
Thyroid	574	0.325	1.4e-22	1.00
Brain - Hypothalamus	170	0.320	6.2e-6	1.00
Brain - Amygdala	129	0.292	7.8e-5	1.00
Brain - Hippocampus	165	0.283	1.9e-5	1.00
Brain - Caudate (basal ganglia)	194	0.279	5.6e-5	1.00
Brain - Nucleus accumbens (basal ganglia)	202	0.278	2.5e-6	1.00
Brain - Frontal Cortex (BA9)	175	0.261	6.7e-5	1.00
Brain - Putamen (basal ganglia)	170	0.258	6.3e-4	0.979
Vagina	141	0.252	8.2e-4	1.00
Brain - Substantia nigra	114	0.242	0.007	0.985
Brain - Anterior cingulate cortex (BA24)	147	0.239	6.1e-4	1.00
Artery - Coronary	213	0.223	9.3e-5	1.00
Nerve - Tibial	532	0.221	1.6e-15	1.00
Esophagus - Mucosa	497	0.216	5.5e-16	1.00
Skin - Not Sun Exposed (Suprapubic)	517	0.214	3.1e-16	1.00
Brain - Spinal cord (cervical c-1)	126	0.213	0.01	1.00
Brain - Cortex	205	0.207	1.1e-3	1.00
Minor Salivary Gland	144	0.205	1.5e-3	1.00
Skin - Sun Exposed (Lower leg)	605	0.196	3.6e-16	1.00
Colon - Sigmoid	318	0.193	2.0e-5	1.00
Artery - Tibial	584	0.188	6.1e-8	1.00
Brain - Cerebellar Hemisphere	175	0.184	0.02	0.984
Spleen	227	0.179	1.8e-3	1.00
Lung	515	0.176	6.8e-7	1.00
Artery - Aorta	387	0.169	1.4e-5	1.00
Stomach	324	0.167	4.9e-6	1.00
Adipose - Subcutaneous	581	0.166	7.0e-9	1.00
Prostate	221	0.165	2.7e-5	1.00
Esophagus - Gastroesophageal Junction	330	0.156	3.9e-4	1.00
Uterus	129	0.154	0.03	0.984
Heart - Atrial Appendage	372	0.144	1.1e-4	1.00
Colon - Transverse	368	0.143	4.5e-4	1.00
Muscle - Skeletal	706	0.139	2.1e-6	1.00
Ovary	167	0.133	0.03	1.00
Kidney - Cortex	73	0.131	0.2	0.937
Heart - Left Ventricle	386	0.131	1.4e-4	1.00
Esophagus - Muscularis	465	0.123	7.6e-5	1.00
Adipose - Visceral (Omentum)	469	0.112	7.8e-7	1.00
Adrenal Gland	233	0.109	0.1	0.905
Breast - Mammary Tissue	396	0.103	3.8e-4	0.995
Testis	322	0.0848	0.02	0.888
Whole Blood	670	0.0820	0.007	0.700
Cells - EBV-transformed lymphocytes	147	0.0770	0.2	0.794
Cells - Cultured fibroblasts	483	0.0754	6.9e-7	0.00
Pituitary	237	0.0336	0.6	0.502
Pancreas	305	0.0248	0.6	0.194
Liver	208	0.00966	0.9	0.257
Small Intestine - Terminal Ileum	174	-0.00794	0.9	0.00

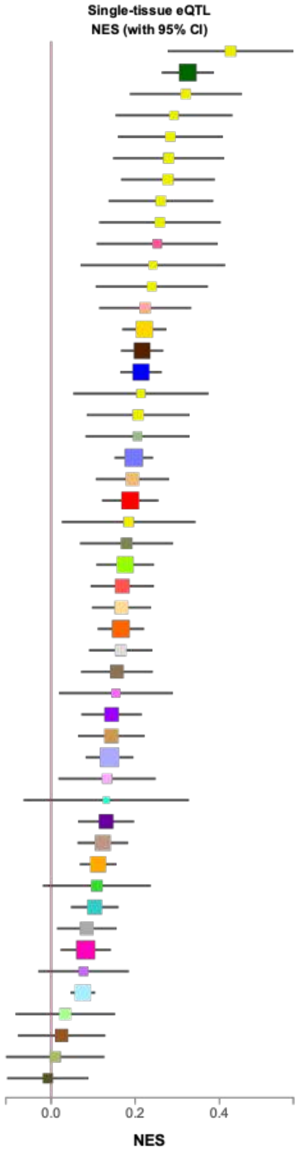

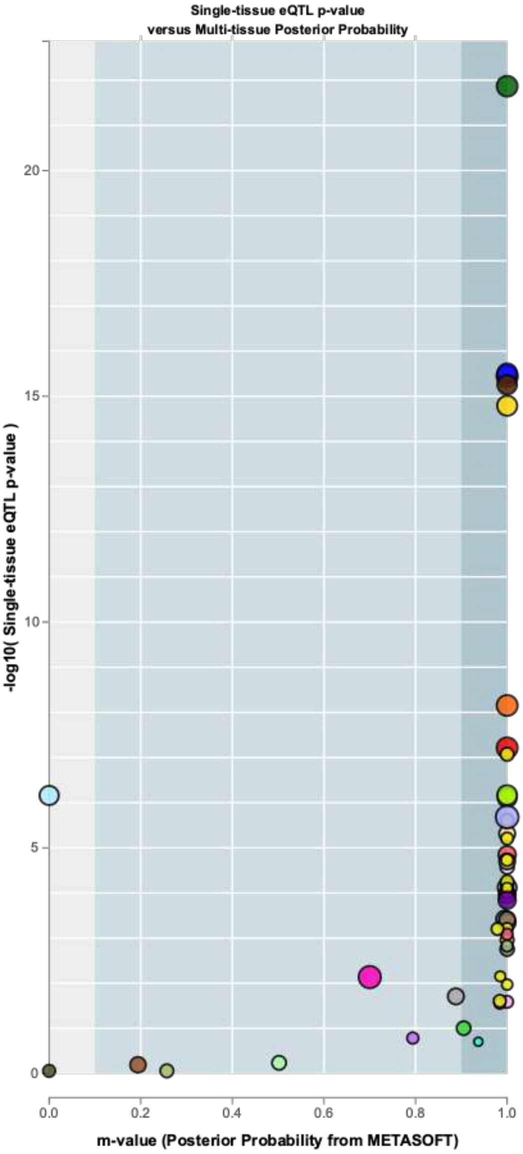

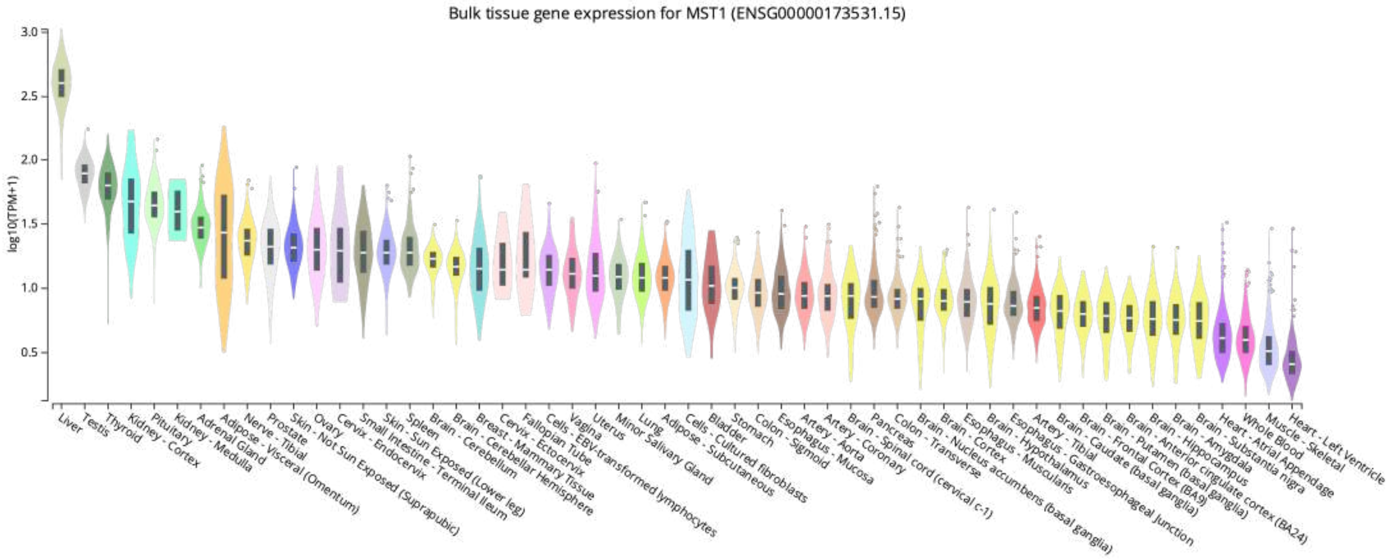

Exon Expression of MST1: ENSG00000173531.15 macrophage stimulating 1 [Source:HGNC Symbol;Acc:HGNC:7380]

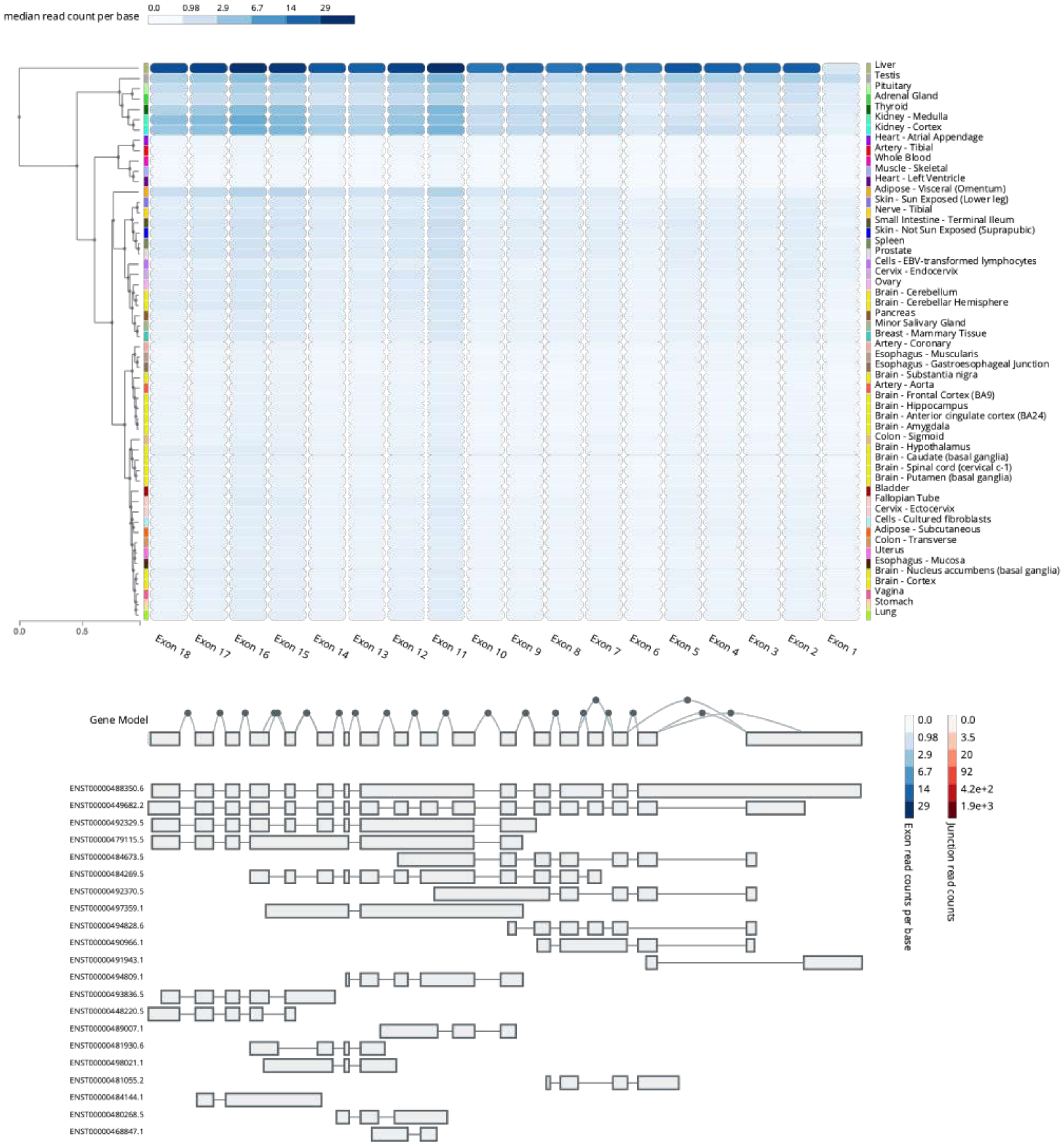

IL21 rs13140464

Tissue	Samples	NES	p-value	m-value
Brain - Substantia nigra	114	0.376	0.02	0.902
Ovary	167	0.177	0.04	0.926
Artery - Coronary	213	0.158	4.2e-3	0.984
Brain - Hypothalamus	170	0.156	0.2	0.842
Artery - Tibial	584	0.150	2.1e-4	0.999
Heart - Atrial Appendage	372	0.144	0.01	0.978
Muscle - Skeletal	706	0.128	0.007	0.988
Brain - Spinal cord (cervical c-1)	126	0.127	0.3	0.831
Adipose - Visceral (Omentum)	469	0.124	2.2e-3	0.994
Breast - Mammary Tissue	396	0.116	0.01	0.983
Testis	322	0.116	0.1	0.882
Heart - Left Ventricle	386	0.109	0.08	0.915
Esophagus - Gastroesophageal Junction	330	0.107	0.09	0.899
Esophagus - Muscularis	465	0.103	0.07	0.910
Adrenal Gland	233	0.0939	0.3	0.876
Pituitary	237	0.0915	0.2	0.832
Nerve - Tibial	532	0.0897	0.04	0.943
Stomach	324	0.0883	0.1	0.844
Minor Salivary Gland	144	0.0854	0.4	0.791
Artery - Aorta	387	0.0844	0.08	0.931
Vagina	141	0.0808	0.4	0.775
Colon - Sigmoid	318	0.0732	0.3	0.803
Lung	515	0.0627	0.03	0.929
Small Intestine - Terminal Ileum	174	0.0433	0.2	0.779
Whole Blood	670	0.0406	0.02	0.687
Prostate	221	0.0311	0.7	0.688
Colon - Transverse	368	0.0218	0.5	0.490
Spleen	227	0.0189	0.8	0.659
Adipose - Subcutaneous	581	0.0140	0.7	0.398
Cells - EBV-transformed lymphocytes	147	0.0127	0.9	0.691
Skin - Sun Exposed (Lower leg)	605	0.00823	0.8	0.314
Brain - Frontal Cortex (BA9)	175	-	-	-
Brain - Amygdala	129	-	-	-
Brain - Caudate (basal ganglia)	194	-	-	-
Brain - Cerebellum	209	-	-	-
Pancreas	305	-	-	-
Brain - Anterior cingulate cortex (BA24)	147	-	-	-
Brain - Hippocampus	165	-	-	-
Brain - Cortex	205	-	-	-
Brain - Nucleus accumbens (basal ganglia)	202	-	-	-
Brain - Cerebellar Hemisphere	175	-	-	-
Cells - Cultured fibroblasts	483	-	-	-
Brain - Putamen (basal ganglia)	170	-	-	-
Thyroid	574	-0.0152	0.6	0.106
Kidney - Cortex	73	-0.0246	0.8	0.653
Skin - Not Sun Exposed (Suprapubic)	517	-0.0296	0.5	0.165
Liver	208	-0.0302	0.7	0.495
Esophagus - Mucosa	497	-0.0561	0.06	0.00500
Uterus	129	-0.0959	0.4	0.602

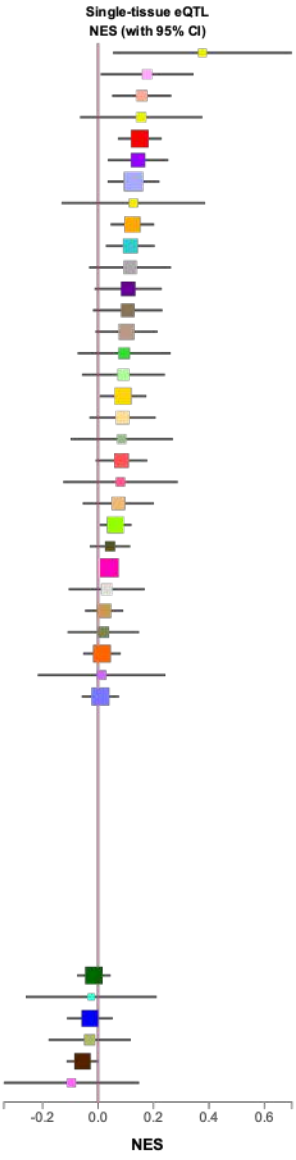

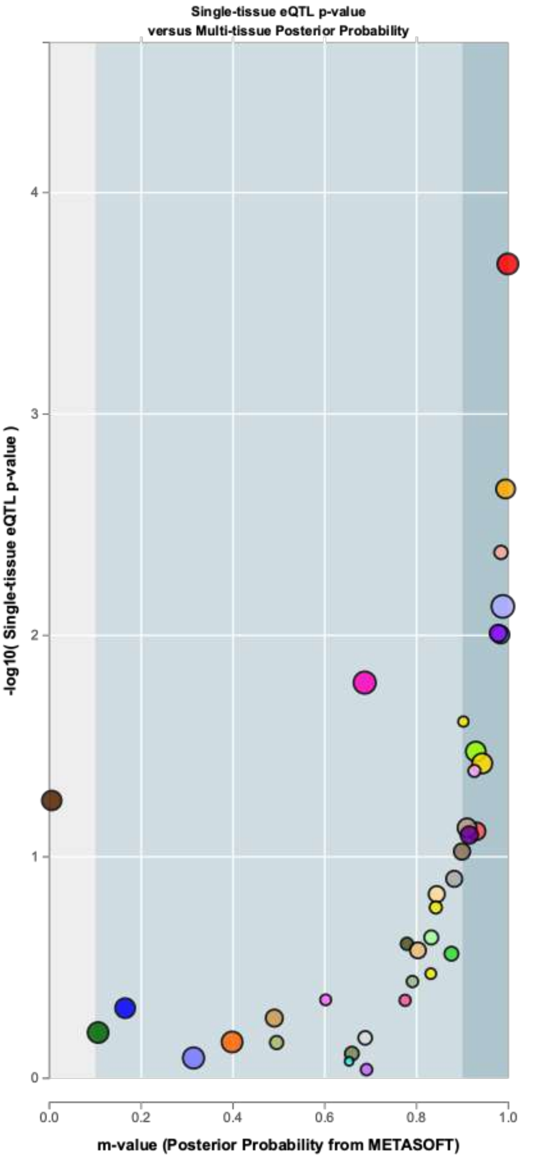

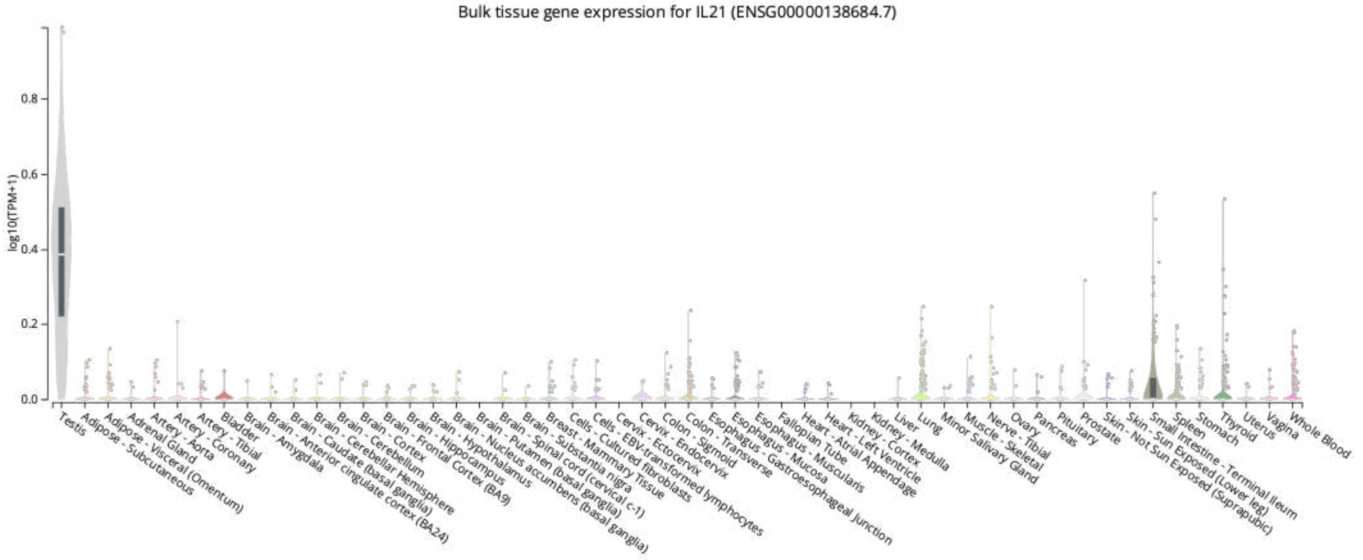

Exon Expression of IL21: ENSG00000138684.7 interleukin 21 [Source:HGNC Symbol;Acc:HGNC:6005]

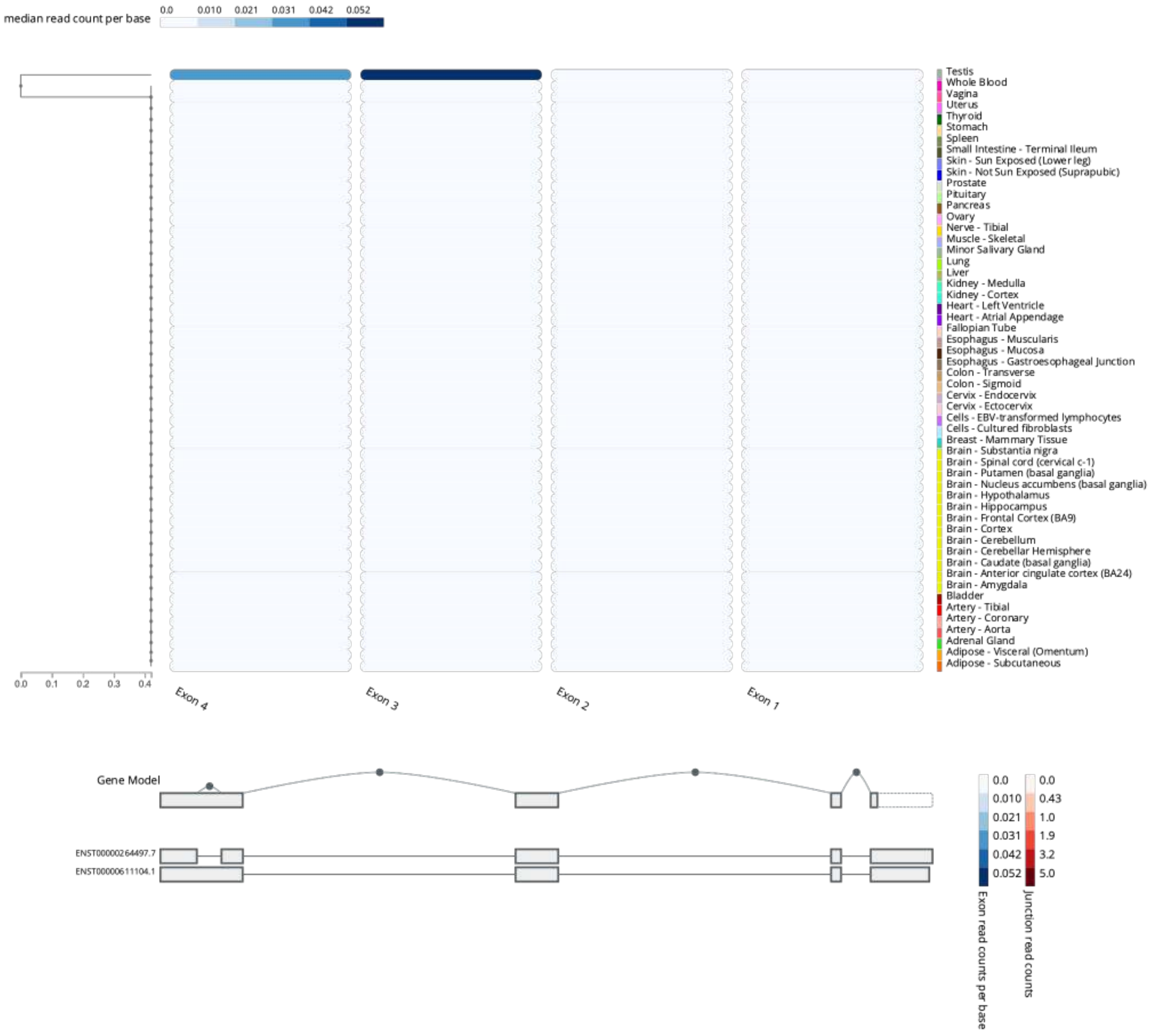

CD226 rs1788097

Tissue	Samples	NES	p-value	m-value
Small Intestine - Terminal Ileum	174	0.0629	0.2	0.0910
Breast - Mammary Tissue	396	0.0460	0.3	0.0140
Brain - Substantia nigra	114	0.0357	0.6	0.314
Prostate	221	0.0308	0.6	0.0930
Ovary	167	0.0286	0.7	0.368
Skin - Not Sun Exposed (Suprapubic)	517	0.0207	0.5	0.0390
Minor Salivary Gland	144	0.0116	0.9	0.420
Cells - Cultured fibroblasts	483	0.00869	0.9	0.235
Thyroid	574	-0.00301	0.9	0.0340
Nerve - Tibial	532	-0.00366	0.9	0.111
Pancreas	305	-0.0103	0.9	0.424
Colon - Transverse	368	-0.0216	0.5	0.185
Skin - Sun Exposed (Lower leg)	605	-0.0244	0.5	0.206
Artery - Aorta	387	-0.0259	0.5	0.406
Muscle - Skeletal	706	-0.0369	0.2	0.349
Esophagus - Muscularis	465	-0.0371	0.4	0.541
Uterus	129	-0.0410	0.7	0.701
Artery - Coronary	213	-0.0456	0.4	0.655
Adipose - Visceral (Omentum)	469	-0.0474	0.2	0.667
Brain - Cerebellar Hemisphere	175	-0.0532	0.5	0.684
Colon - Sigmoid	318	-0.0537	0.3	0.689
Esophagus - Mucosa	497	-0.0539	0.09	0.743
Esophagus - Gastroesophageal Junction	330	-0.0581	0.3	0.663
Brain - Anterior cingulate cortex (BA24)	147	-0.0603	0.5	0.637
Brain - Amygdala	129	-0.0607	0.5	0.724
Adrenal Gland	233	-0.0738	0.3	0.702
Artery - Tibial	584	-0.0754	0.04	0.916
Adipose - Subcutaneous	581	-0.0773	0.03	0.940
Whole Blood	670	-0.0778	5.1e-5	1.00
Spleen	227	-0.0860	0.1	0.869
Vagina	141	-0.0926	0.3	0.747
Liver	208	-0.101	0.1	0.911
Brain - Frontal Cortex (BA9)	175	-0.102	0.1	0.868
Kidney - Cortex	73	-0.109	0.4	0.697
Stomach	324	-0.114	0.005	0.987
Brain - Hippocampus	165	-0.114	0.09	0.897
Lung	515	-0.120	5.3e-4	1.00
Brain - Spinal cord (cervical c-1)	126	-0.122	0.1	0.880
Brain - Cerebellum	209	-0.126	0.09	0.861
Heart - Left Ventricle	386	-0.133	0.04	0.955
Brain - Hypothalamus	170	-0.134	0.05	0.931
Heart - Atrial Appendage	372	-0.145	0.01	0.984
Brain - Cortex	205	-0.177	0.01	0.968
Brain - Nucleus accumbens (basal ganglia)	202	-0.187	1.7e-3	1.00
Cells - EBV-transformed lymphocytes	147	-0.193	0.06	0.862
Brain - Putamen (basal ganglia)	170	-0.229	3.9e-4	0.992
Brain - Caudate (basal ganglia)	194	-0.251	1.2e-4	0.999
Pituitary	237	-0.254	3.9e-4	0.980
Testis	322	-0.334	5.4e-9	1.00

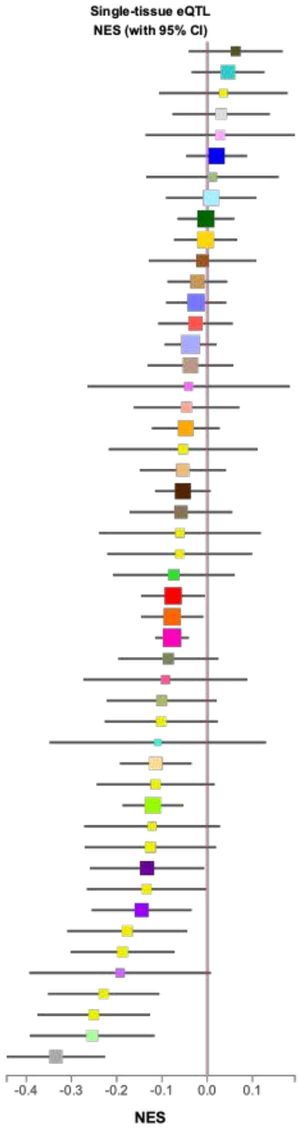

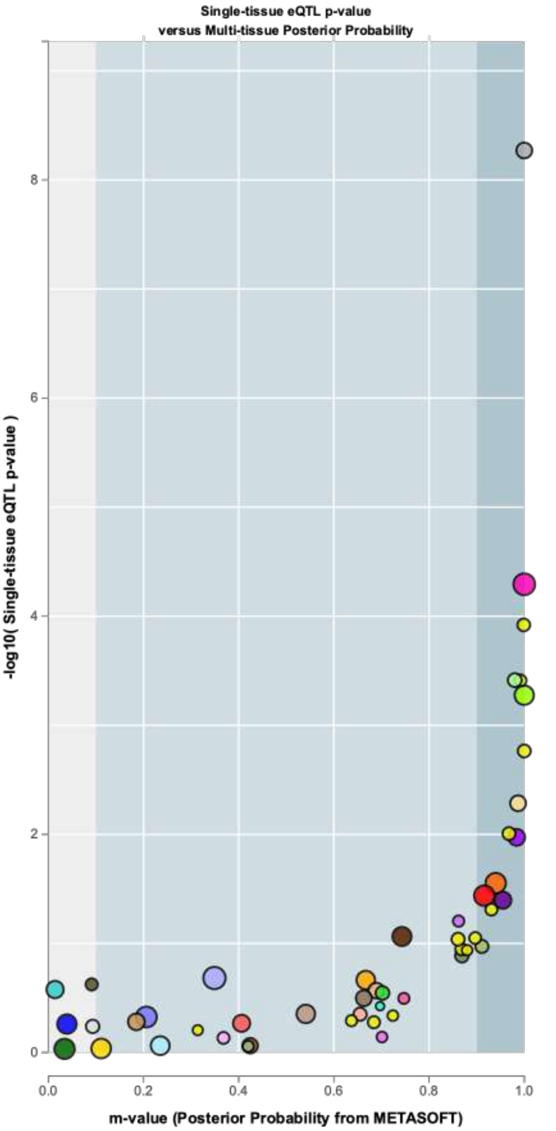

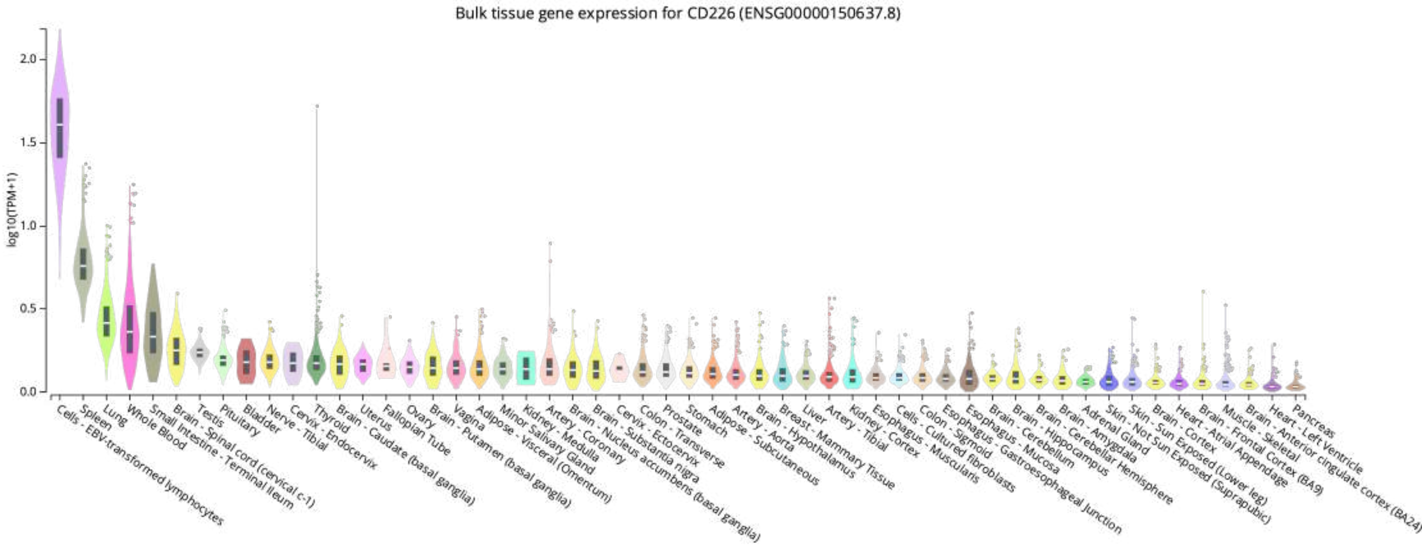

Exon Expression of CD226: ENSG00000150637.8 CD226 molecule [Source:HGNC Symbol;Acc:HGNC:16961]

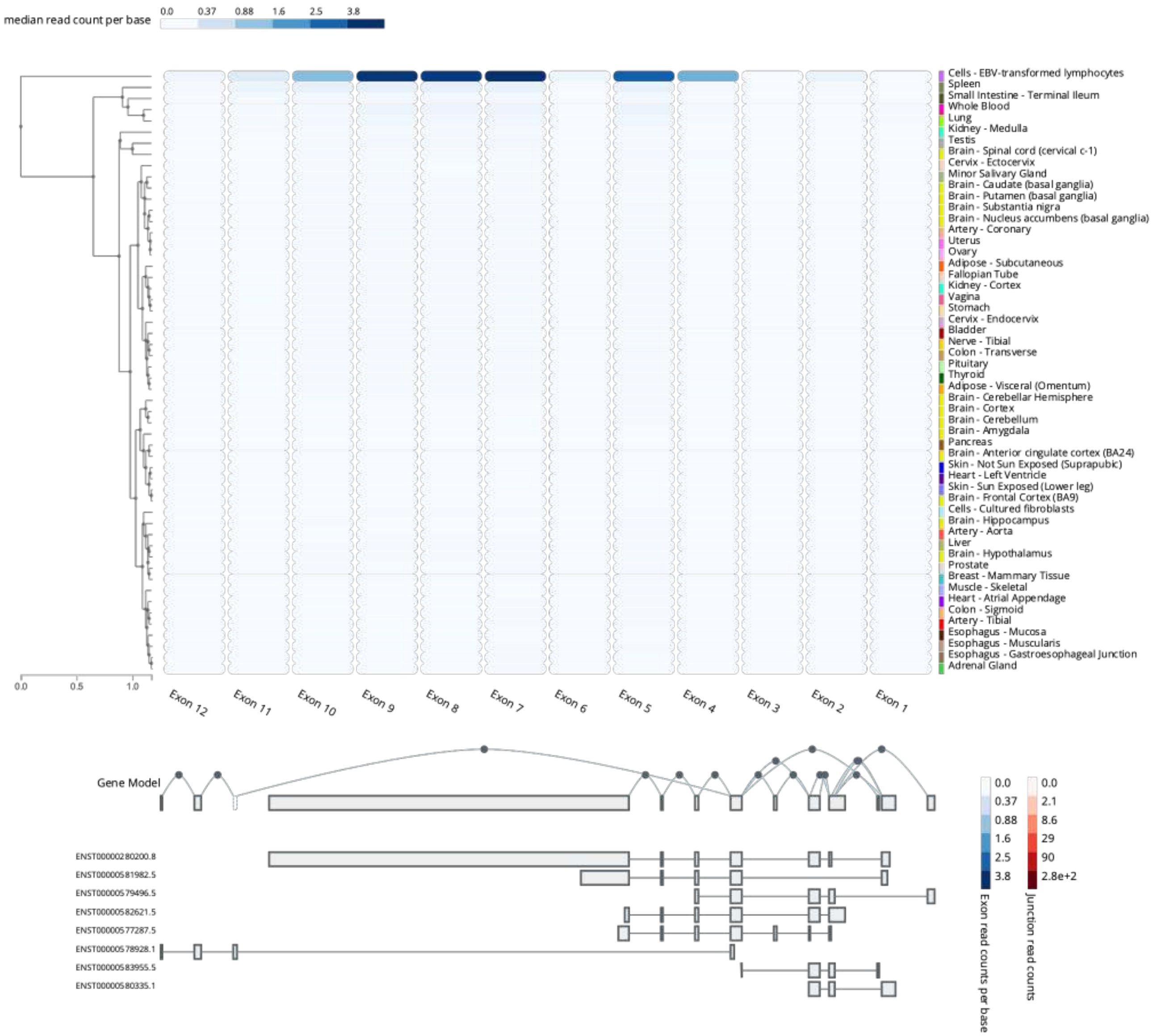

CCDC88B rs663743

Tissue	Samples	NES	p-value	m-value
Cells - Cultured fibroblasts	483	0.0844	0.1	0.00
Uterus	129	0.0281	0.8	0.280
Testis	322	0.0230	0.5	0.00
Liver	208	-0.00216	1	0.00
Small Intestine - Terminal Ileum	174	-0.0216	0.5	0.00
Breast - Mammary Tissue	396	-0.0251	0.4	0.00
Adrenal Gland	233	-0.0495	0.4	0.113
Lung	515	-0.0525	0.01	0.00
Muscle - Skeletal	706	-0.0600	0.09	0.00200
Whole Blood	670	-0.0961	9.1e-8	0.547
Thyroid	574	-0.0975	6.1e-6	0.604
Adipose - Subcutaneous	581	-0.0997	3.4e-5	0.726
Brain - Substantia nigra	114	-0.101	0.2	0.804
Heart - Left Ventricle	386	-0.104	0.008	0.778
Spleen	227	-0.112	0.006	0.932
Adipose - Visceral (Omentum)	469	-0.117	7.6e-6	1.00
Pancreas	305	-0.117	0.007	0.966
Brain - Spinal cord (cervical c-1)	126	-0.131	0.006	0.987
Prostate	221	-0.137	1.5e-3	1.00
Artery - Coronary	213	-0.138	1.8e-3	1.00
Artery - Tibial	584	-0.141	1.3e-9	1.00
Vagina	141	-0.141	0.1	0.847
Kidney - Cortex	73	-0.143	0.07	0.881
Nerve - Tibial	532	-0.155	1.2e-8	1.00
Brain - Hypothalamus	170	-0.156	1.6e-3	1.00
Stomach	324	-0.158	2.9e-4	1.00
Brain - Anterior cingulate cortex (BA24)	147	-0.160	0.02	1.00
Artery - Aorta	387	-0.162	4.0e-8	1.00
Brain - Amygdala	129	-0.165	0.02	0.988
Minor Salivary Gland	144	-0.187	0.04	0.980
Cells - EBV-transformed lymphocytes	147	-0.190	0.006	0.997
Skin - Not Sun Exposed (Suprapubic)	517	-0.194	4.6e-9	1.00
Heart - Atrial Appendage	372	-0.200	9.8e-7	1.00
Brain - Hippocampus	165	-0.201	2.2e-4	1.00
Colon - Transverse	368	-0.215	1.5e-10	1.00
Skin - Sun Exposed (Lower leg)	605	-0.233	9.2e-17	1.00
Pituitary	237	-0.257	1.6e-7	1.00
Ovary	167	-0.281	7.7e-4	1.00
Colon - Sigmoid	318	-0.298	3.3e-14	1.00
Esophagus - Muscularis	465	-0.298	2.7e-24	1.00
Brain - Caudate (basal ganglia)	194	-0.298	1.0e-9	1.00
Brain - Putamen (basal ganglia)	170	-0.323	5.1e-7	1.00
Esophagus - Gastroesophageal Junction	330	-0.329	3.1e-16	1.00
Brain - Nucleus accumbens (basal ganglia)	202	-0.342	2.1e-7	1.00
Brain - Frontal Cortex (BA9)	175	-0.360	7.9e-10	1.00
Esophagus - Mucosa	497	-0.403	2.1e-21	1.00
Brain - Cortex	205	-0.469	1.6e-13	1.00
Brain - Cerebellar Hemisphere	175	-0.828	1.3e-20	1.00
Brain - Cerebellum	209	-0.925	1.5e-26	1.00

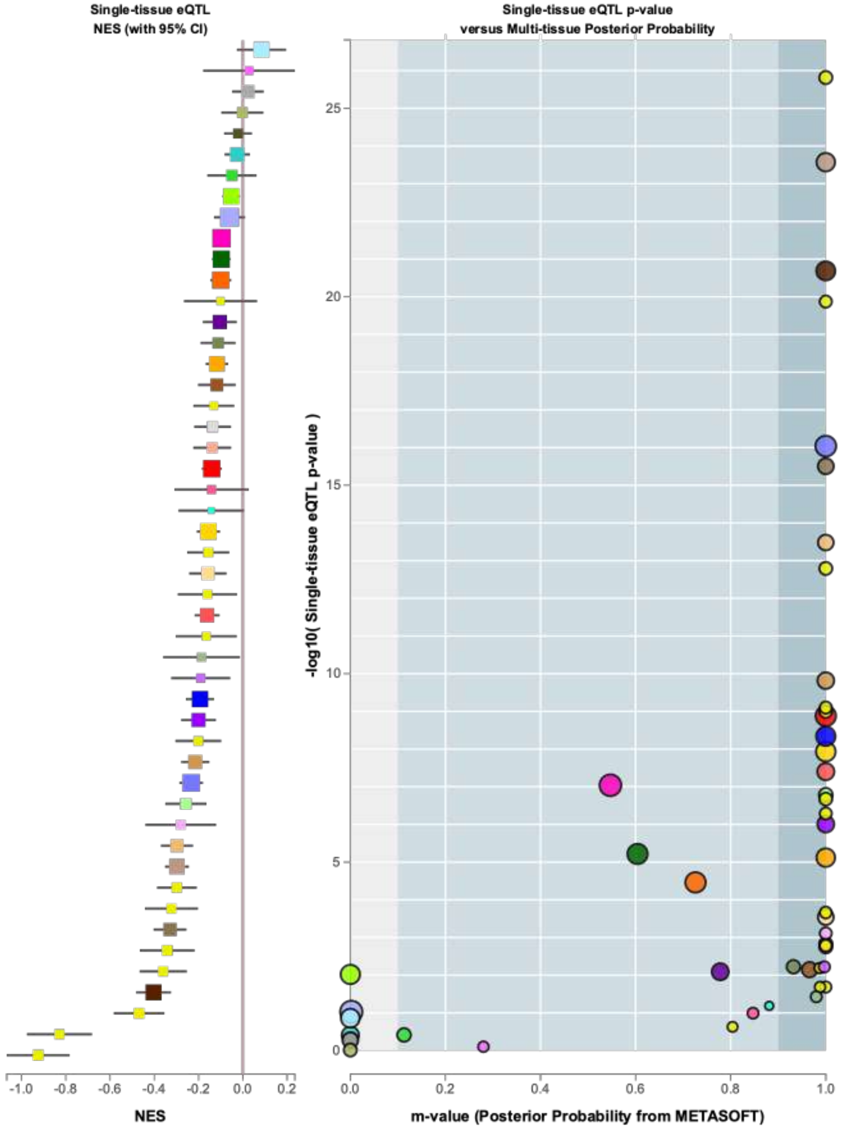

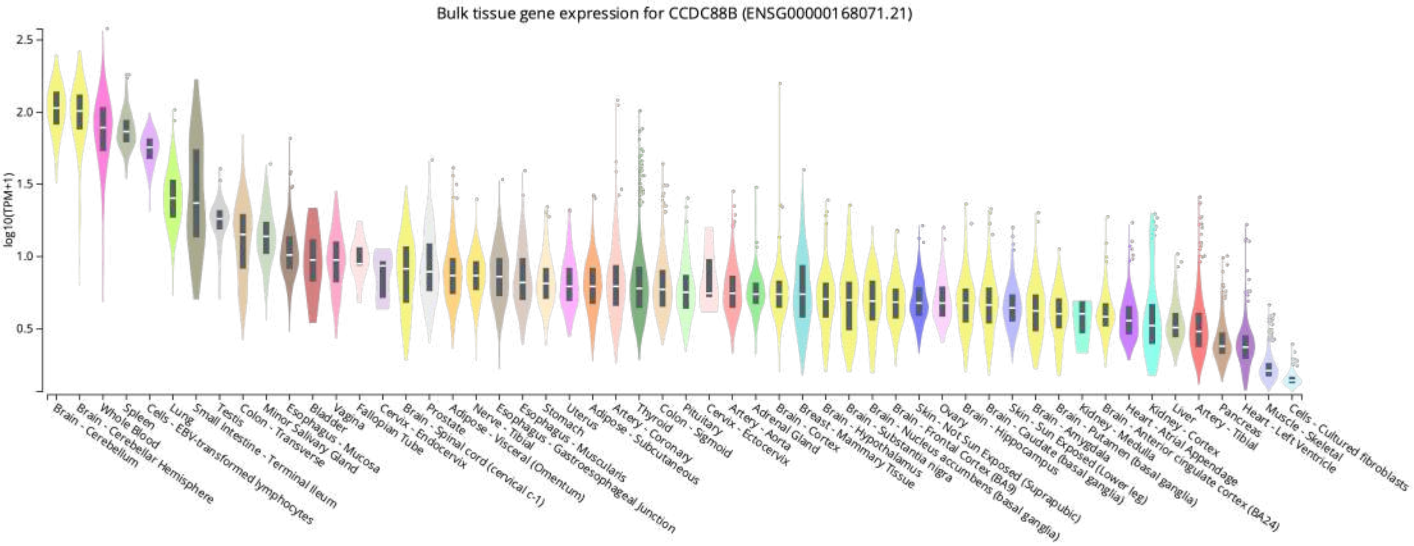

Exon Expression of CCDC88B: ENSG00000168071.21 coiled-coil domain containing 88B [Source:HGNC Symbol;Acc:HGNC:26757]

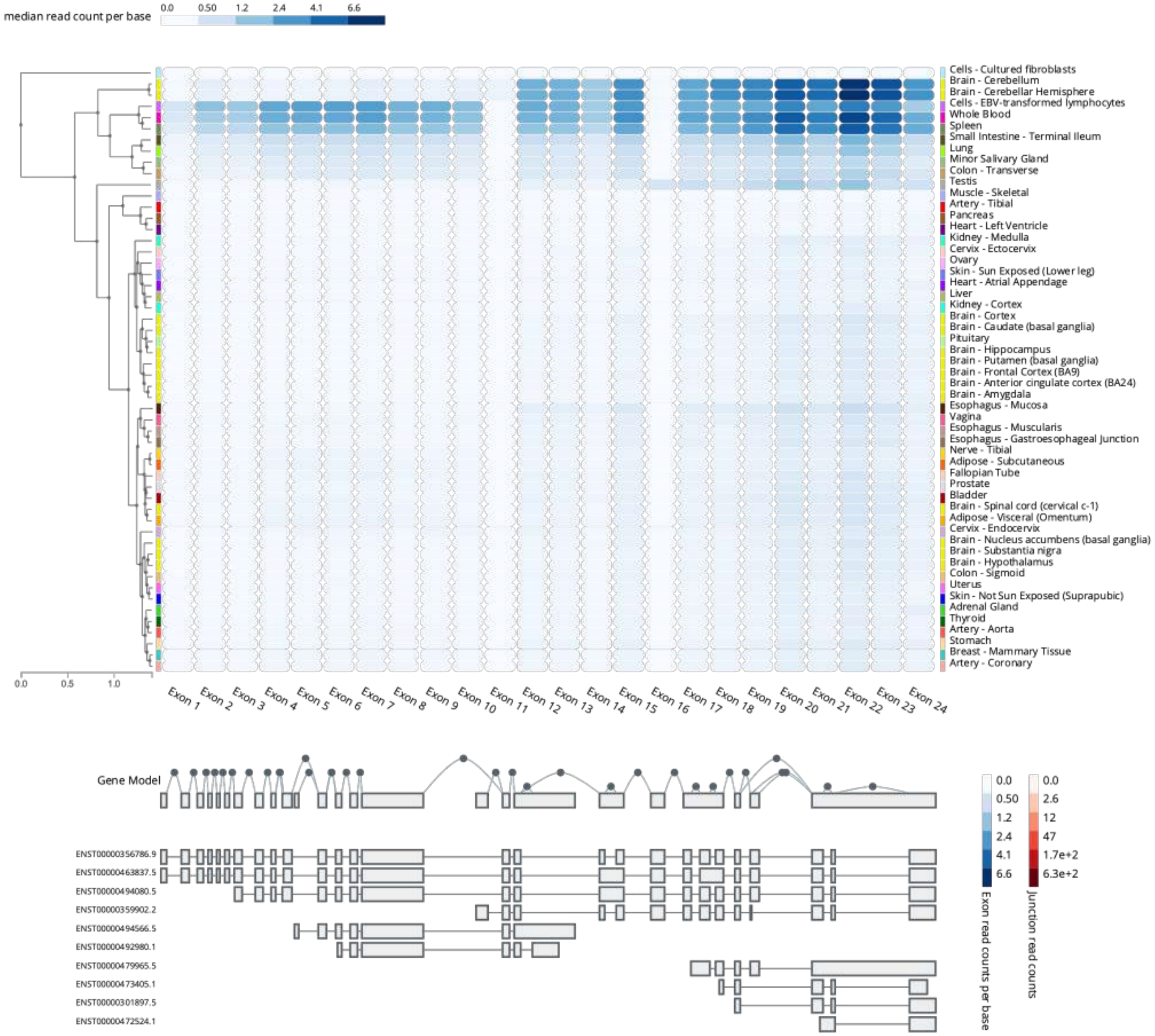

CD28 rs7426056

Tissue	Samples	NES	p-value	m-value
Brain - Substantia nigra	114	0.376	0.02	0.902
Ovary	167	0.177	0.04	0.926
Artery - Coronary	213	0.158	4.2e-3	0.984
Brain - Hypothalamus	170	0.156	0.2	0.842
Artery - Tibial	584	0.150	2.1e-4	0.999
Heart - Atrial Appendage	372	0.144	0.01	0.978
Muscle - Skeletal	706	0.128	0.007	0.988
Brain - Spinal cord (cervical c-1)	126	0.127	0.3	0.831
Adipose - Visceral (Omentum)	469	0.124	2.2e-3	0.994
Breast - Mammary Tissue	396	0.116	0.01	0.983
Testis	322	0.116	0.1	0.882
Heart - Left Ventricle	386	0.109	0.08	0.915
Esophagus - Gastroesophageal Junction	330	0.107	0.09	0.899
Esophagus - Muscularis	465	0.103	0.07	0.910
Adrenal Gland	233	0.0939	0.3	0.876
Pituitary	237	0.0915	0.2	0.832
Nerve - Tibial	532	0.0897	0.04	0.943
Stomach	324	0.0883	0.1	0.844
Minor Salivary Gland	144	0.0854	0.4	0.791
Artery - Aorta	387	0.0844	0.08	0.931
Vagina	141	0.0808	0.4	0.775
Colon - Sigmoid	318	0.0732	0.3	0.803
Lung	515	0.0627	0.03	0.929
Small Intestine - Terminal Ileum	174	0.0433	0.2	0.779
Whole Blood	670	0.0406	0.02	0.687
Prostate	221	0.0311	0.7	0.688
Colon - Transverse	368	0.0218	0.5	0.490
Spleen	227	0.0189	0.8	0.659
Adipose - Subcutaneous	581	0.0140	0.7	0.398
Cells - EBV-transformed lymphocytes	147	0.0127	0.9	0.691
Skin - Sun Exposed (Lower leg)	605	0.00823	0.8	0.314
Brain - Frontal Cortex (BA9)	175	-	-	-
Brain - Amygdala	129	-	-	-
Brain - Caudate (basal ganglia)	194	-	-	-
Brain - Cerebellum	209	-	-	-
Pancreas	305	-	-	-
Brain - Anterior cingulate cortex (BA24)	147	-	-	-
Brain - Hippocampus	165	-	-	-
Brain - Cortex	205	-	-	-
Brain - Nucleus accumbens (basal ganglia)	202	-	-	-
Brain - Cerebellar Hemisphere	175	-	-	-
Cells - Cultured fibroblasts	483	-	-	-
Brain - Putamen (basal ganglia)	170	-	-	-
Thyroid	574	-0.0152	0.6	0.106
Kidney - Cortex	73	-0.0246	0.8	0.653
Skin - Not Sun Exposed (Suprapubic)	517	-0.0296	0.5	0.165
Liver	208	-0.0302	0.7	0.495
Esophagus - Mucosa	497	-0.0561	0.06	0.00500
Uterus	129	-0.0959	0.4	0.602

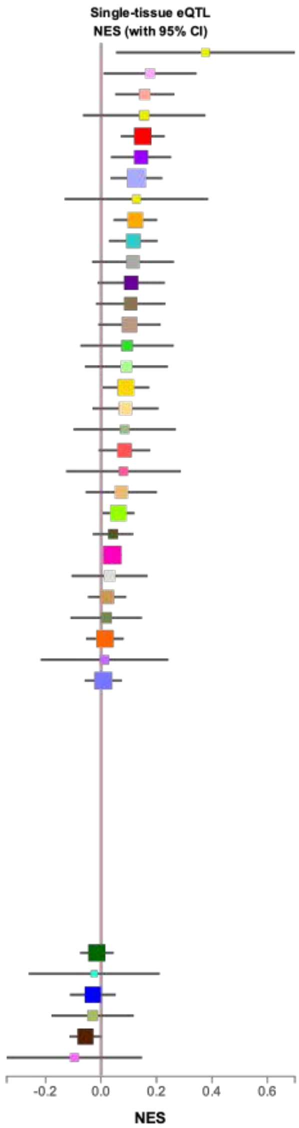

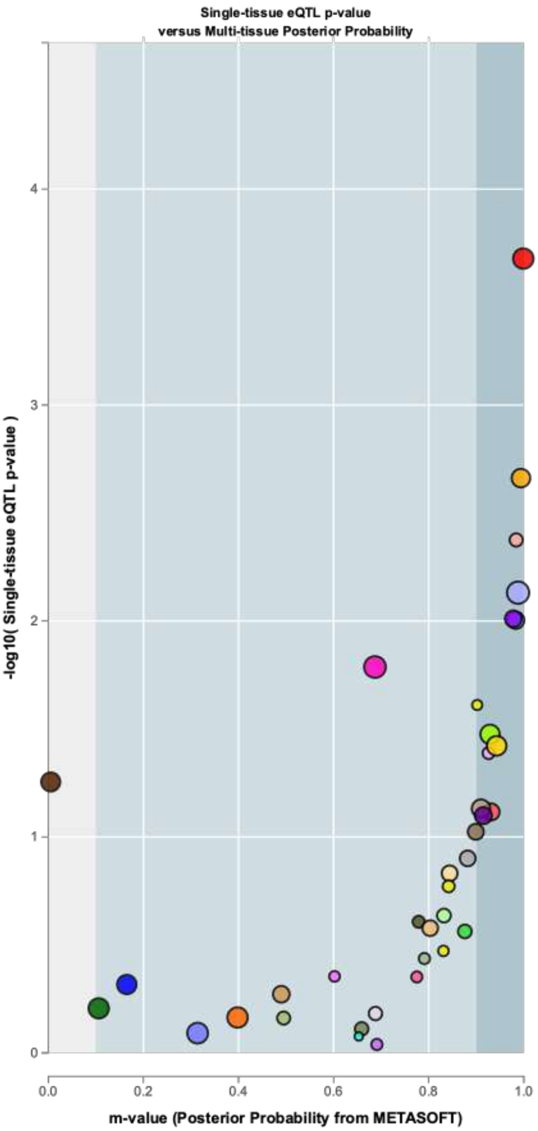

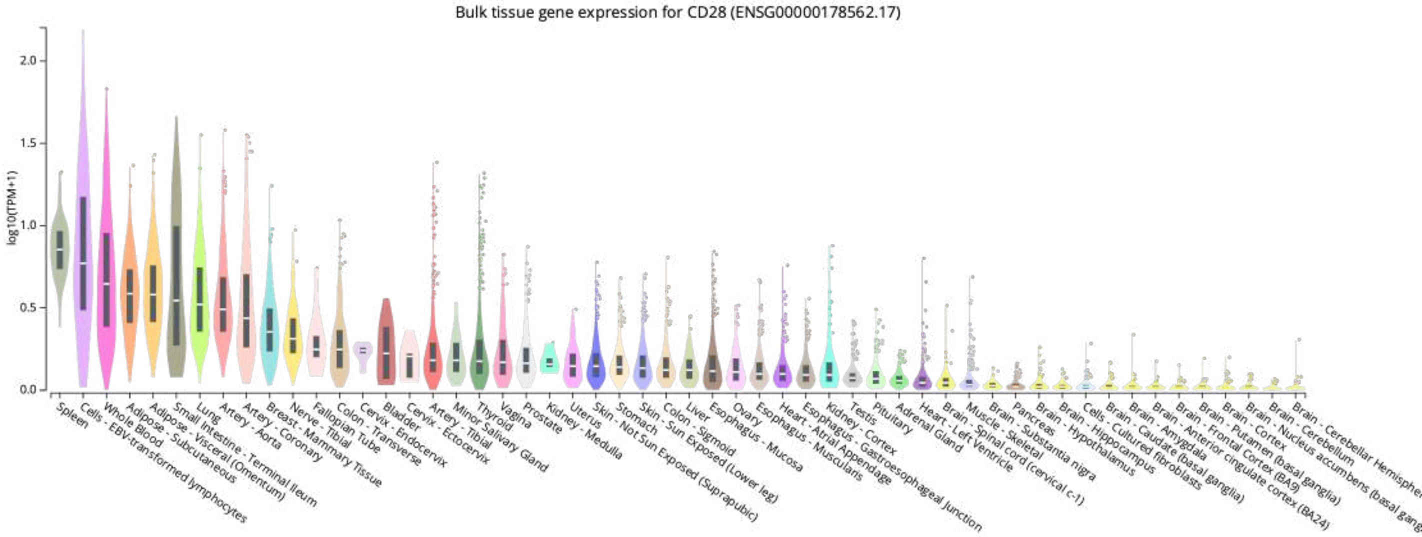

Exon Expression of CD28: ENSG00000178562.17 CD28 molecule [Source:HGNC Symbol;Acc:HGNC:1653]

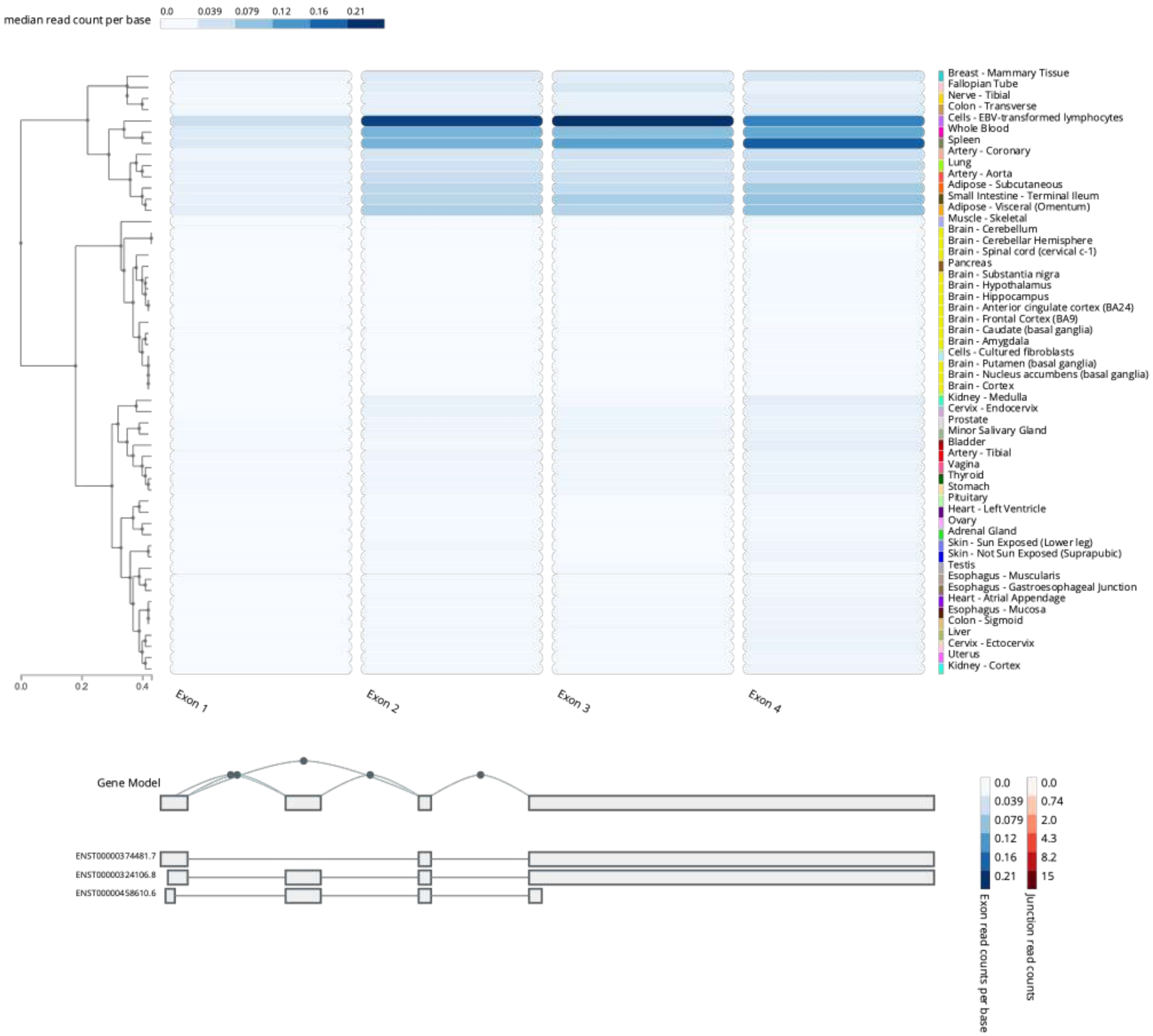

PRKD2 rs60652743

Tissue	Samples	NES	p-value	m-value
Brain - Cortex	205	0.517	1.1e-10	1.00
Brain - Anterior cingulate cortex (BA24)	147	0.516	5.2e-9	1.00
Cells - Cultured fibroblasts	483	0.503	3.4e-36	1.00
Brain - Amygdala	129	0.492	5.2e-7	1.00
Adrenal Gland	233	0.445	2.4e-7	1.00
Brain - Hippocampus	165	0.428	2.1e-7	1.00
Nerve - Tibial	532	0.412	3.5e-23	1.00
Brain - Frontal Cortex (BA9)	175	0.399	1.0e-6	1.00
Artery - Aorta	387	0.390	4.8e-15	1.00
Pituitary	237	0.389	4.1e-9	1.00
Liver	208	0.368	4.6e-6	1.00
Pancreas	305	0.368	1.3e-11	1.00
Brain - Substantia nigra	114	0.364	2.0e-4	1.00
Esophagus - Muscularis	465	0.360	4.1e-21	1.00
Cells - EBV-transformed lymphocytes	147	0.359	2.0e-4	1.00
Brain - Hypothalamus	170	0.345	3.2e-8	1.00
Heart - Atrial Appendage	372	0.341	4.8e-14	1.00
Esophagus - Gastroesophageal Junction	330	0.330	5.8e-13	1.00
Brain - Spinal cord (cervical c-1)	126	0.322	9.6e-5	1.00
Muscle - Skeletal	706	0.318	2.6e-19	1.00
Brain - Putamen (basal ganglia)	170	0.311	4.7e-5	1.00
Colon - Sigmoid	318	0.299	3.4e-10	1.00
Artery - Tibial	584	0.286	1.4e-25	1.00
Artery - Coronary	213	0.270	1.1e-5	1.00
Thyroid	574	0.250	1.1e-11	1.00
Whole Blood	670	0.243	1.3e-31	1.00
Testis	322	0.242	2.9e-9	1.00
Breast - Mammary Tissue	396	0.240	2.0e-9	1.00
Stomach	324	0.226	1.1e-8	1.00
Brain - Caudate (basal ganglia)	194	0.223	1.7e-3	0.960
Spleen	227	0.222	1.1e-3	1.00
Brain - Nucleus accumbens (basal ganglia)	202	0.215	2.4e-4	1.00
Ovary	167	0.212	0.03	0.956
Adipose - Visceral (Omentum)	469	0.206	4.1e-9	1.00
Small Intestine - Terminal Ileum	174	0.203	3.1e-4	1.00
Minor Salivary Gland	144	0.203	4.6e-3	0.979
Adipose - Subcutaneous	581	0.200	9.8e-10	1.00
Heart - Left Ventricle	386	0.197	3.9e-8	1.00
Brain - Cerebellum	209	0.196	7.0e-4	1.00
Prostate	221	0.189	6.4e-5	1.00
Lung	515	0.176	9.0e-10	1.00
Colon - Transverse	368	0.163	1.6e-7	1.00
Uterus	129	0.142	0.1	0.933
Brain - Cerebellar Hemisphere	175	0.139	0.08	0.846
Kidney - Cortex	73	0.110	0.4	0.859
Esophagus - Mucosa	497	0.0722	0.07	0.00
Vagina	141	0.0716	0.5	0.584
Skin - Not Sun Exposed (Suprapubic)	517	0.0496	0.04	0.00
Skin - Sun Exposed (Lower leg)	605	-0.00312	0.9	0.00

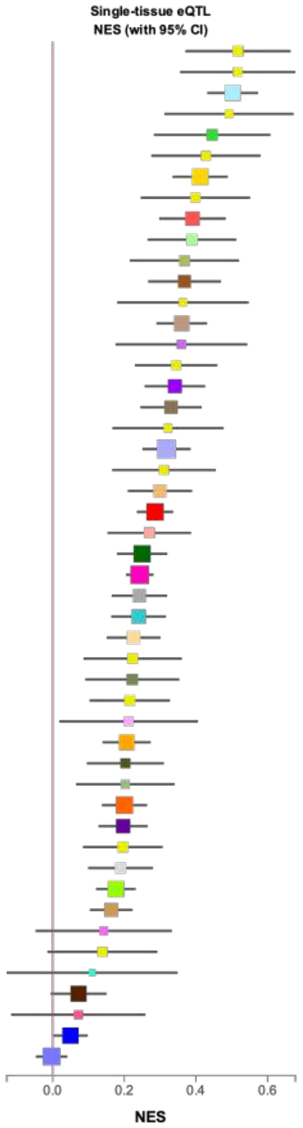

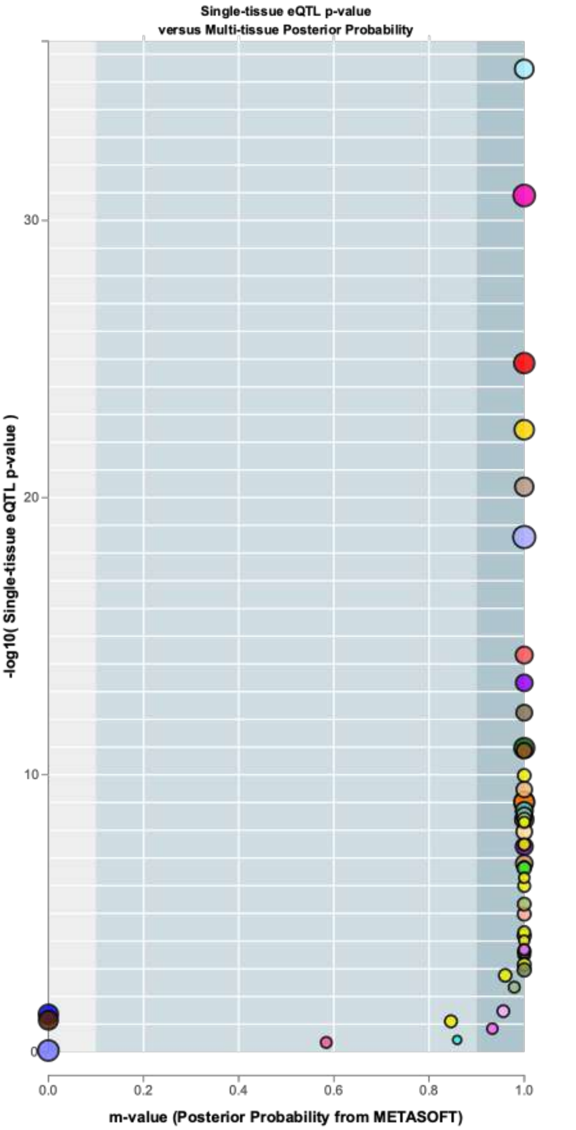

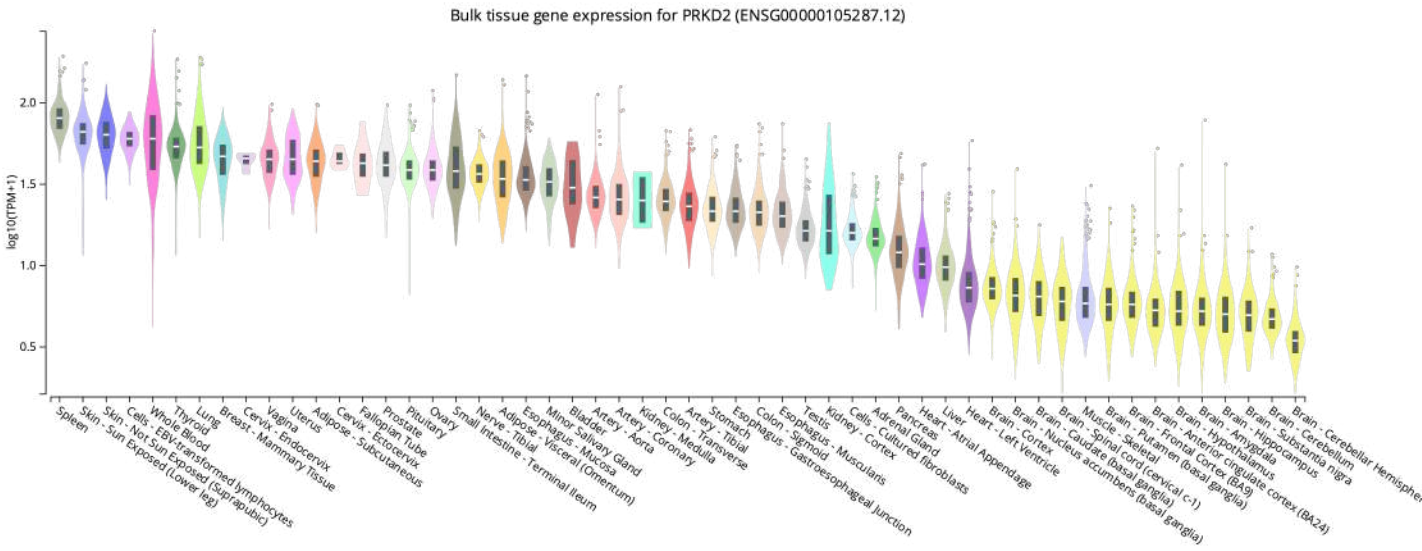

Exon Expression of PRKD2: ENSG00000105287.12 protein kinase D2 [Source:HGNC Symbol;Acc:HGNC:17293]

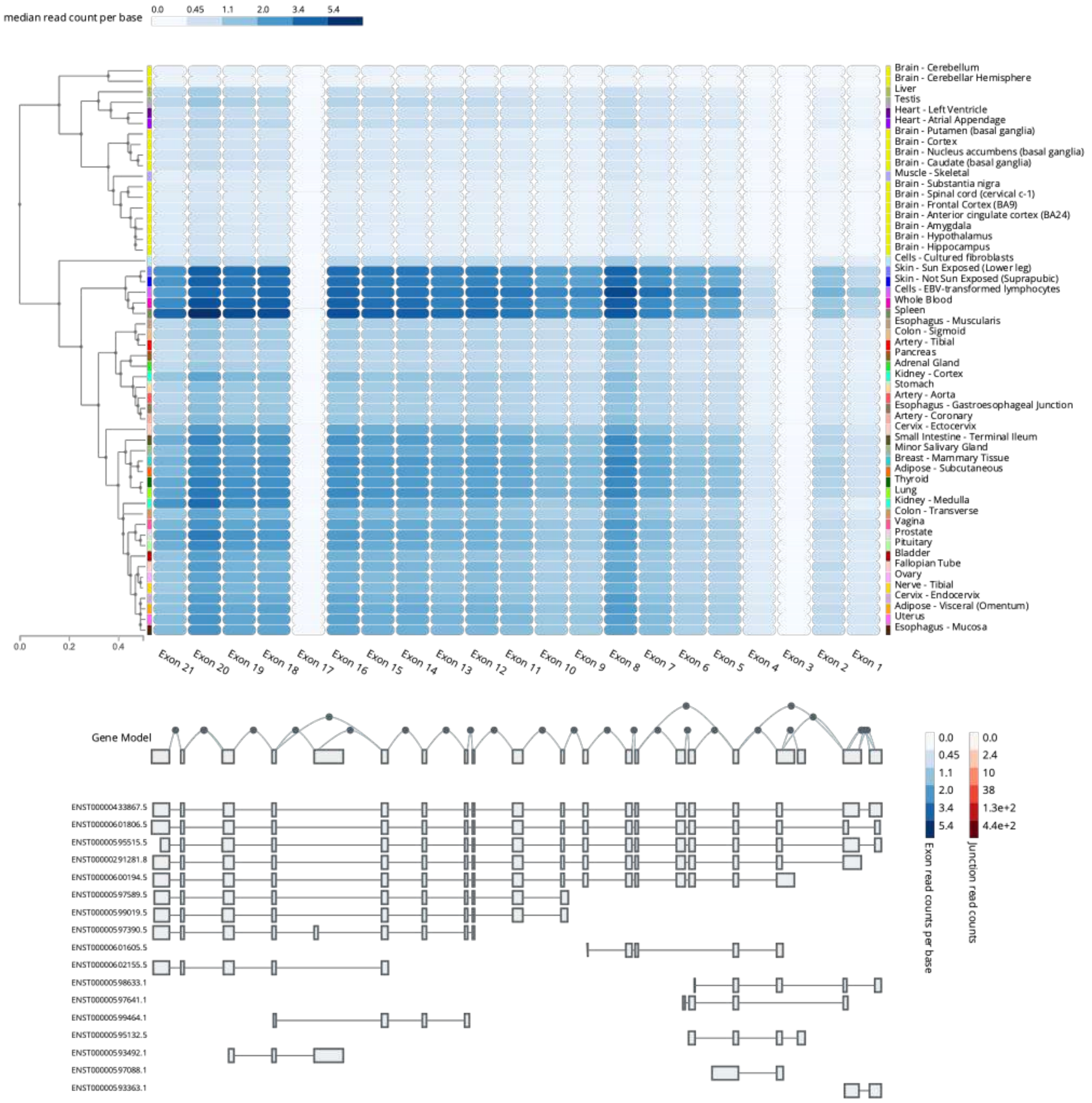

MMEL1 rs3748816

Tissue	Samples	NES	p-value	m-value
Brain - Hypothalamus	170	0.257	1.5e-4	0.00
Brain - Hippocampus	165	0.222	2.5e-3	0.00
Brain - Anterior cingulate cortex (BA24)	147	0.189	0.04	0.00
Pancreas	305	0.0607	0.4	0.00
Cells - EBV-transformed lymphocytes	147	0.0341	0.7	0.00
Esophagus - Mucosa	497	0.0261	0.5	0.00
Brain - Amygdala	129	0.0252	0.8	0.00
Stomach	324	0.0141	0.7	0.00
Muscle - Skeletal	706	-	-	-
Heart - Left Ventricle	386	-	-	-
Cells - Cultured fibroblasts	483	-	-	-
Nerve - Tibial	532	-0.00201	1	0.00
Breast - Mammary Tissue	396	-0.00823	0.9	0.00
Pituitary	237	-0.0112	0.9	0.00
Brain - Frontal Cortex (BA9)	175	-0.0158	0.8	0.00
Brain - Cortex	205	-0.0166	0.8	0.00
Brain - Cerebellum	209	-0.0379	0.7	0.00
Skin - Not Sun Exposed (Suprapubic)	517	-0.0556	0.2	0.00
Testis	322	-0.0723	3.2e-3	0.00
Brain - Spinal cord (cervical c-1)	126	-0.106	0.3	0.00
Vagina	141	-0.110	0.3	0.00
Brain - Substantia nigra	114	-0.127	0.2	0.0100
Esophagus - Muscularis	465	-0.145	1.9e-3	0.00
Brain - Cerebellar Hemisphere	175	-0.148	0.2	0.0100
Prostate	221	-0.149	0.009	0.00
Artery - Aorta	387	-0.157	0.01	0.00
Skin - Sun Exposed (Lower leg)	605	-0.167	1.8e-4	0.00
Colon - Sigmoid	318	-0.176	3.8e-4	0.00
Adipose - Subcutaneous	581	-0.187	1.8e-5	0.00
Artery - Coronary	213	-0.198	0.005	0.00200
Colon - Transverse	368	-0.214	7.3e-8	0.0170
Esophagus - Gastroesophageal Junction	330	-0.238	3.5e-5	0.369
Minor Salivary Gland	144	-0.249	0.01	0.451
Ovary	167	-0.258	0.02	0.499
Kidney - Cortex	73	-0.259	0.1	0.486
Uterus	129	-0.260	0.03	0.426
Brain - Putamen (basal ganglia)	170	-0.277	1.3e-3	0.954
Heart - Atrial Appendage	372	-0.325	4.3e-7	1.00
Adrenal Gland	233	-0.331	2.4e-5	0.977
Adipose - Visceral (Omentum)	469	-0.346	5.7e-23	1.00
Small Intestine - Terminal Ileum	174	-0.390	4.8e-15	1.00
Thyroid	574	-0.431	2.7e-28	1.00
Artery - Tibial	584	-0.448	2.8e-26	1.00
Brain - Caudate (basal ganglia)	194	-0.463	2.7e-12	1.00
Whole Blood	670	-0.494	1.6e-25	1.00
Liver	208	-0.659	1.6e-14	1.00
Brain - Nucleus accumbens (basal ganglia)	202	-0.706	3.7e-24	1.00
Lung	515	-0.732	4.3e-65	1.00
Spleen	227	-0.747	2.5e-25	1.00

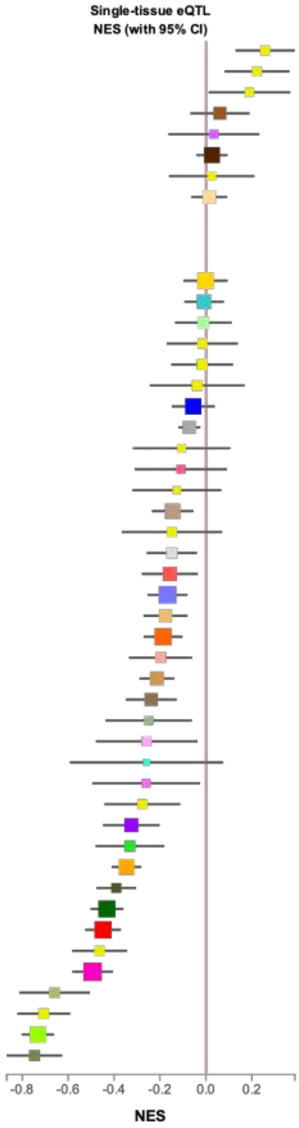

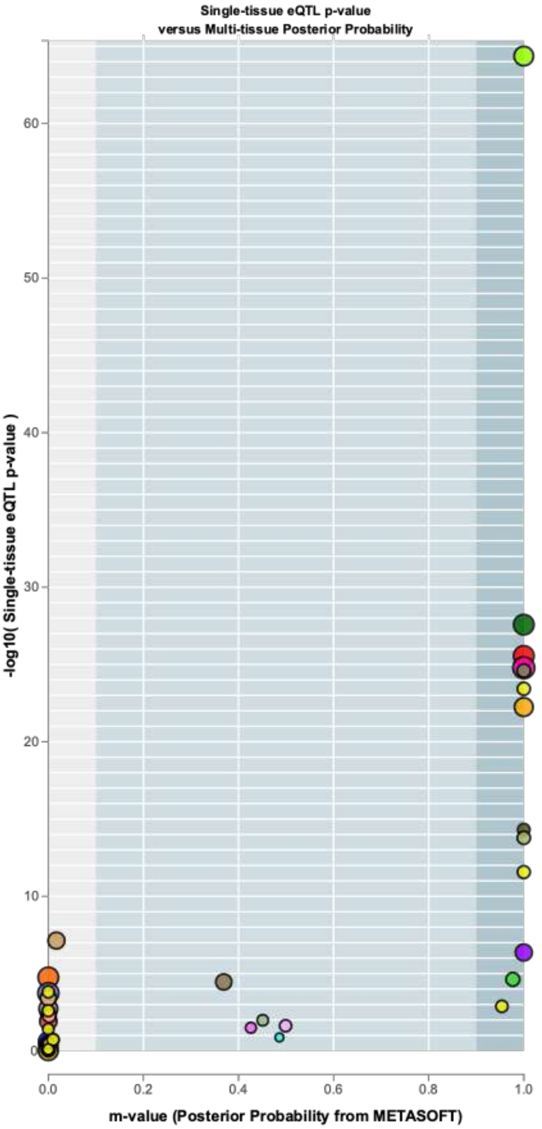

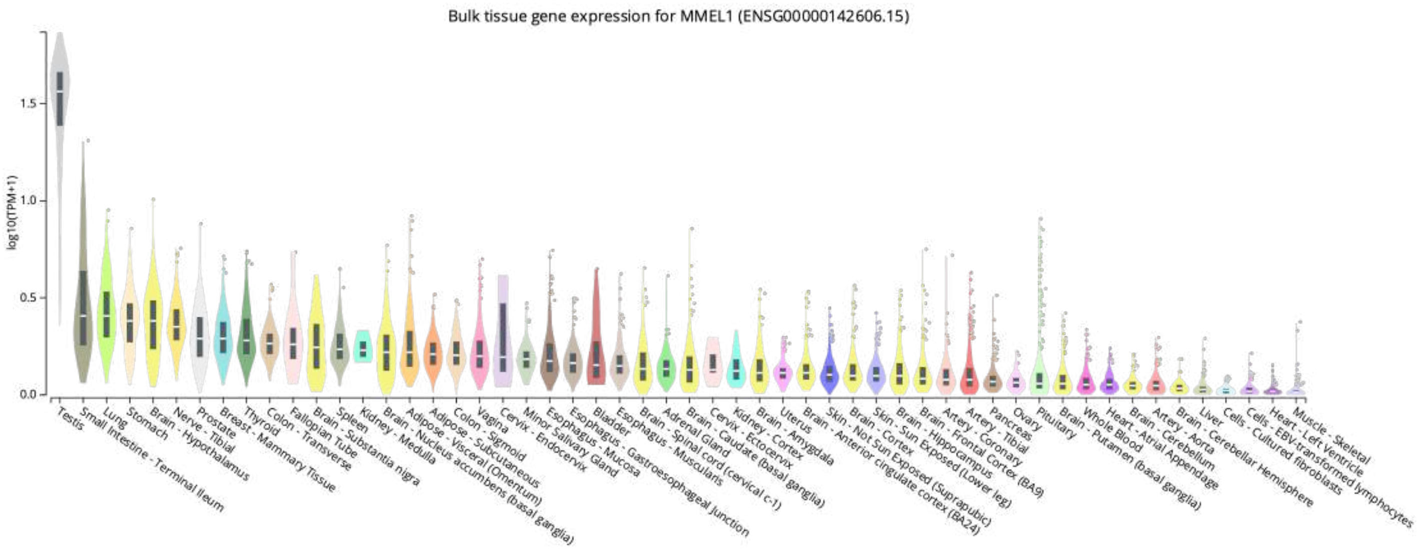

Exon Expression of MMEL1: ENSG00000142606.15 membrane metalloendopeptidase like 1 [Source:HGNC Symbol;Acc:HGNC:14668]

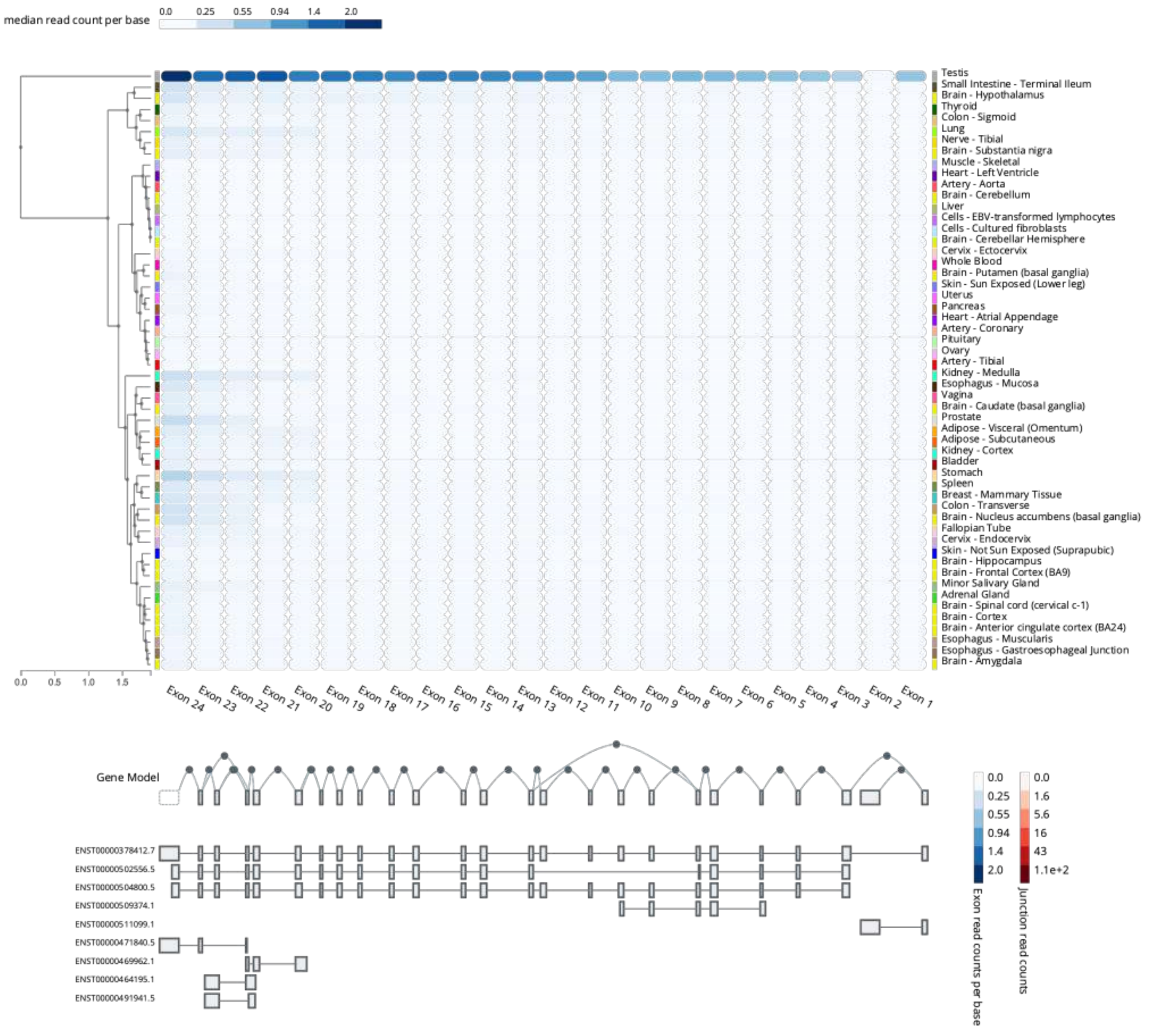

CLEC16A rs725613

Tissue	Samples	NES	p-value	m-value
Liver	208	0.176	1.5e-4	0.998
Small Intestine - Terminal Ileum	174	0.155	5.1e-4	0.998
Uterus	129	0.120	0.1	0.578
Spleen	227	0.112	0.08	0.658
Vagina	141	0.103	0.1	0.615
Testis	322	0.100	7.2e-4	0.993
Brain - Amygdala	129	0.0995	0.1	0.610
Colon - Transverse	368	0.0915	2.2e-3	0.989
Adipose - Subcutaneous	581	0.0910	3.0e-4	1.00
Brain - Caudate (basal ganglia)	194	0.0895	0.1	0.550
Cells - Cultured fibroblasts	483	0.0823	4.0e-4	0.996
Breast - Mammary Tissue	396	0.0780	0.02	0.921
Artery - Coronary	213	0.0735	0.3	0.512
Whole Blood	670	0.0664	0.005	0.946
Adrenal Gland	233	0.0644	0.3	0.418
Brain - Spinal cord (cervical c-1)	126	0.0605	0.5	0.434
Brain - Frontal Cortex (BA9)	175	0.0574	0.4	0.416
Cells - EBV-transformed lymphocytes	147	0.0536	0.3	0.498
Colon - Sigmoid	318	0.0405	0.3	0.341
Nerve - Tibial	532	0.0345	0.1	0.263
Brain - Cortex	205	0.0337	0.5	0.360
Adipose - Visceral (Omentum)	469	0.0304	0.4	0.252
Brain - Hippocampus	165	0.0291	0.6	0.329
Brain - Hypothalamus	170	0.0284	0.6	0.279
Heart - Left Ventricle	386	0.0257	0.3	0.115
Esophagus - Muscularis	465	0.0203	0.5	0.138
Skin - Sun Exposed (Lower leg)	605	0.0199	0.3	0.0300
Esophagus - Mucosa	497	0.0188	0.5	0.0780
Pancreas	305	0.0176	0.7	0.210
Prostate	221	0.0147	0.8	0.194
Minor Salivary Gland	144	0.00662	0.9	0.256
Heart - Atrial Appendage	372	0.00511	0.9	0.0120
Brain - Substantia nigra	114	0.00433	0.9	0.204
Ovary	167	0.000325	1	0.180
Skin - Not Sun Exposed (Suprapubic)	517	-0.00113	1	0.0110
Artery - Tibial	584	-0.00804	0.8	0.0100
Stomach	324	-0.0291	0.4	0.00300
Muscle - Skeletal	706	-0.0315	0.2	0.00
Brain - Anterior cingulate cortex (BA24)	147	-0.0371	0.6	0.117
Esophagus - Gastroesophageal Junction	330	-0.0490	0.3	0.0200
Brain - Nucleus accumbens (basal ganglia)	202	-0.0500	0.4	0.0740
Pituitary	237	-0.0551	0.1	0.00
Artery - Aorta	387	-0.0599	0.08	0.00
Brain - Putamen (basal ganglia)	170	-0.0630	0.3	0.0500
Brain - Cerebellum	209	-0.0663	0.4	0.0540
Brain - Cerebellar Hemisphere	175	-0.0745	0.3	0.0390
Lung	515	-0.0817	4.3e-3	0.00
Kidney - Cortex	73	-0.107	0.2	0.112
Thyroid	574	-0.127	1.5e-5	0.00

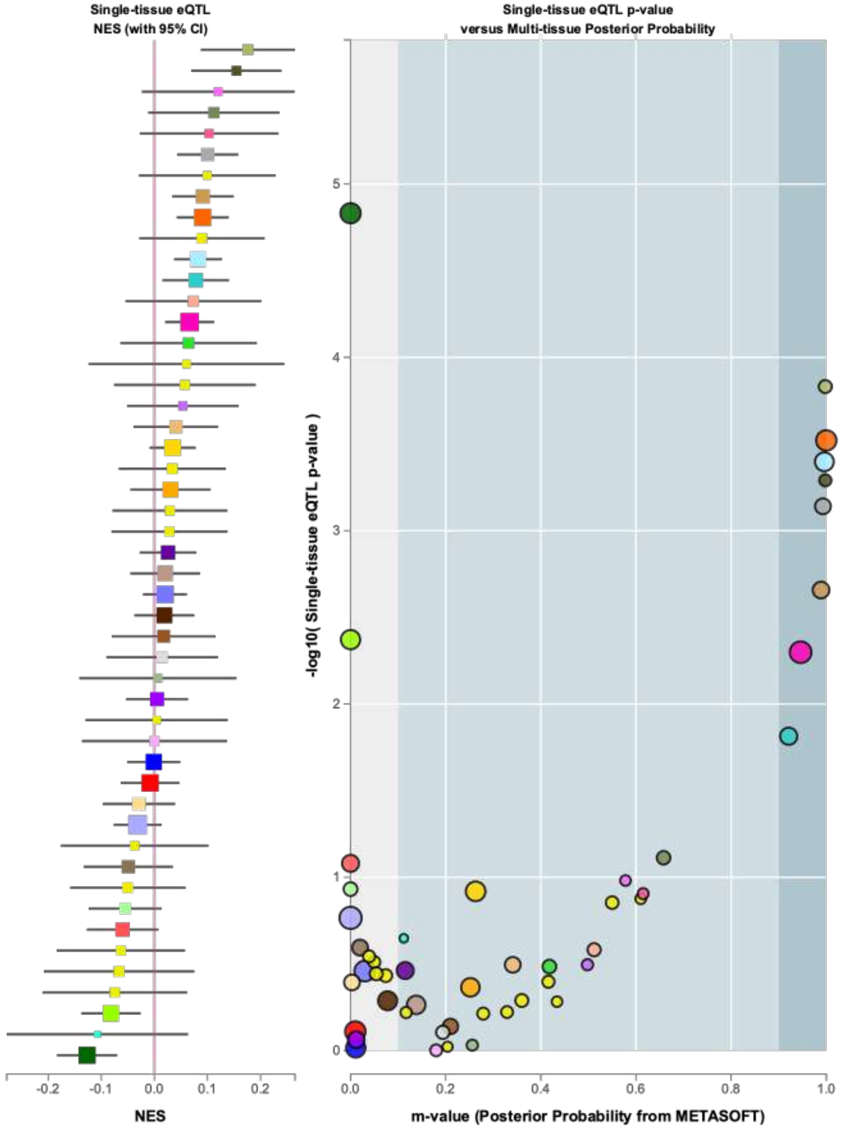

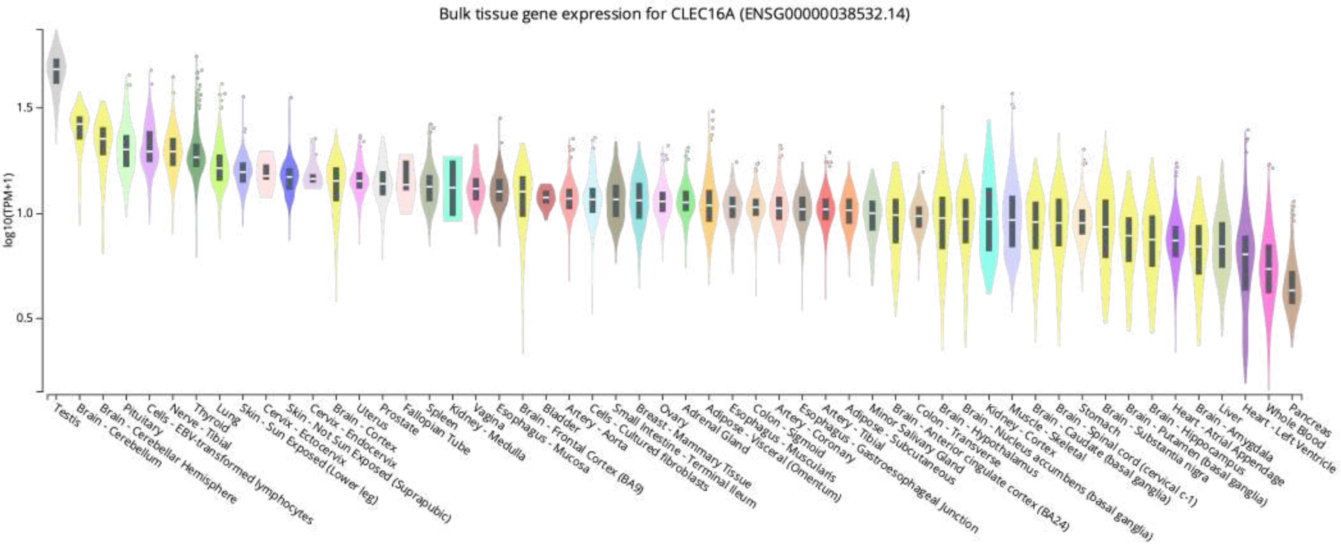

Exon Expression of CLEC16A: ENSG00000038532.14 C-type lectin domain containing 16A [Source:HGNC Symbol;Acc:HGNC:29013]

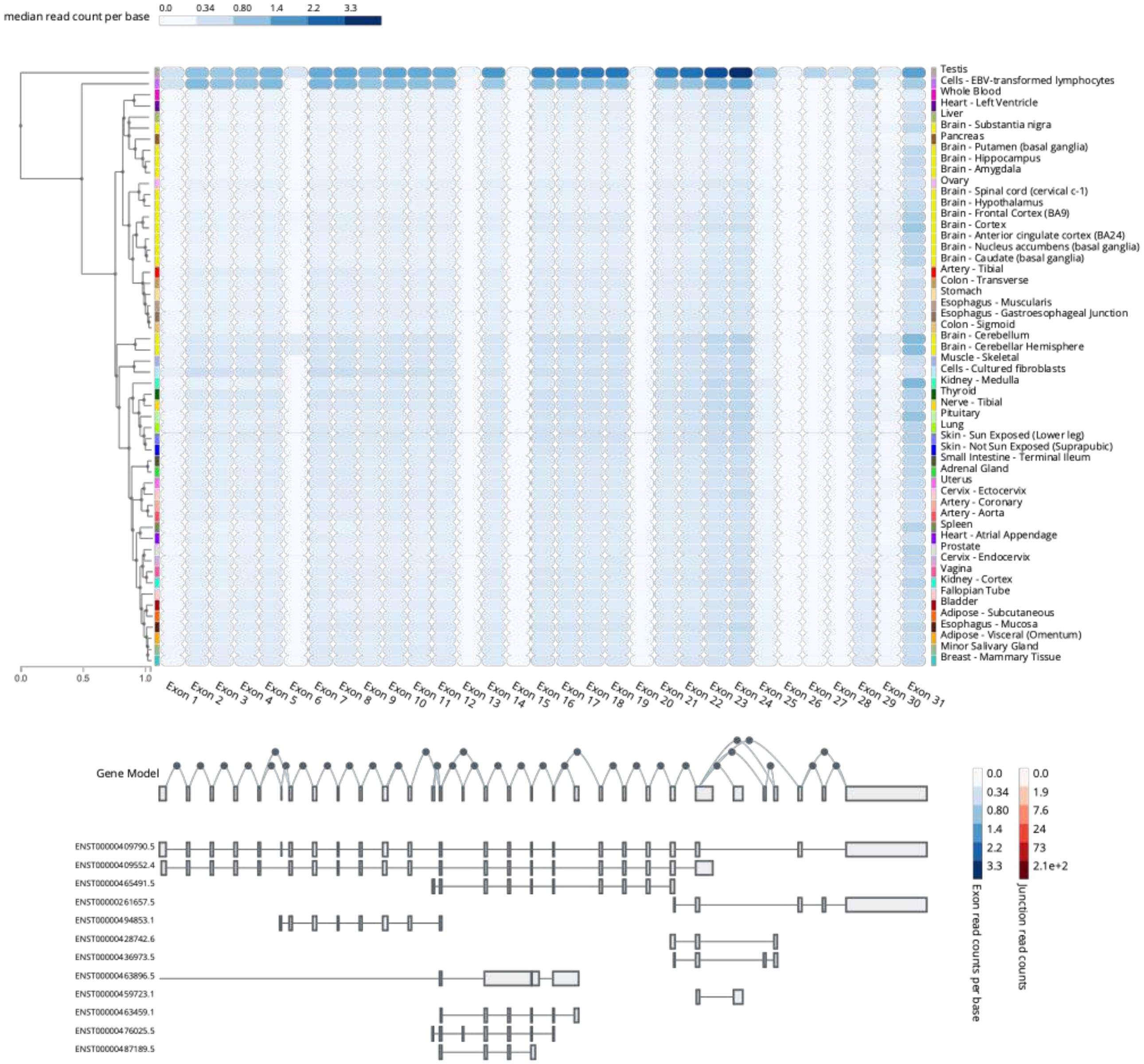

SIK2 rs7937682

Tissue	Samples	NES	p-value	m-value
Esophagus - Mucosa	497	0.260	5.1e-12	1.00
Minor Salivary Gland	144	0.244	0.01	0.923
Cells - EBV-transformed lymphocytes	147	0.232	1.7e-3	0.995
Brain - Caudate (basal ganglia)	194	0.217	8.4e-4	0.999
Cells - Cultured fibroblasts	483	0.204	2.1e-10	1.00
Brain - Spinal cord (cervical c-1)	126	0.190	0.01	0.875
Whole Blood	670	0.173	9.3e-14	1.00
Brain - Substantia nigra	114	0.165	0.09	0.723
Skin - Sun Exposed (Lower leg)	605	0.148	5.6e-10	1.00
Vagina	141	0.136	0.06	0.875
Adrenal Gland	233	0.132	0.005	0.928
Kidney - Cortex	73	0.121	0.3	0.480
Skin - Not Sun Exposed (Suprapubic)	517	0.115	1.0e-4	1.00
Spleen	227	0.102	0.06	0.696
Prostate	221	0.0964	0.06	0.628
Brain - Putamen (basal ganglia)	170	0.0911	0.1	0.575
Esophagus - Muscularis	465	0.0844	0.02	0.673
Esophagus - Gastroesophageal Junction	330	0.0796	0.1	0.404
Thyroid	574	0.0772	0.02	0.535
Adipose - Subcutaneous	581	0.0704	2.3e-3	0.309
Colon - Transverse	368	0.0699	0.08	0.307
Small Intestine - Terminal Ileum	174	0.0658	0.3	0.291
Testis	322	0.0534	0.3	0.168
Lung	515	0.0500	0.08	0.0660
Heart - Atrial Appendage	372	0.0493	0.2	0.0850
Brain - Hippocampus	165	0.0381	0.6	0.157
Brain - Cerebellum	209	0.0305	0.7	0.163
Brain - Cortex	205	0.0298	0.6	0.0970
Stomach	324	0.0286	0.5	0.0210
Breast - Mammary Tissue	396	0.0281	0.2	0.00
Adipose - Visceral (Omentum)	469	0.0245	0.3	0.00
Brain - Cerebellar Hemisphere	175	0.0214	0.8	0.0980
Brain - Anterior cingulate cortex (BA24)	147	0.0186	0.8	0.217
Colon - Sigmoid	318	0.0175	0.7	0.00900
Ovary	167	0.0116	0.8	0.0820
Uterus	129	0.00983	0.9	0.144
Artery - Tibial	584	0.000998	1	0.00
Liver	208	-0.00140	1	0.0840
Brain - Frontal Cortex (BA9)	175	-0.00401	0.9	0.00900
Brain - Nucleus accumbens (basal ganglia)	202	-0.00666	0.9	0.00
Muscle - Skeletal	706	-0.00883	0.7	0.00
Artery - Aorta	387	-0.0139	0.7	0.00
Pancreas	305	-0.0142	0.7	0.00
Artery - Coronary	213	-0.0163	0.8	0.00700
Heart - Left Ventricle	386	-0.0221	0.5	0.00
Pituitary	237	-0.0255	0.6	0.00
Nerve - Tibial	532	-0.0282	0.4	0.00
Brain - Amygdala	129	-0.0622	0.5	0.0800
Brain - Hypothalamus	170	-0.177	0.05	0.00100

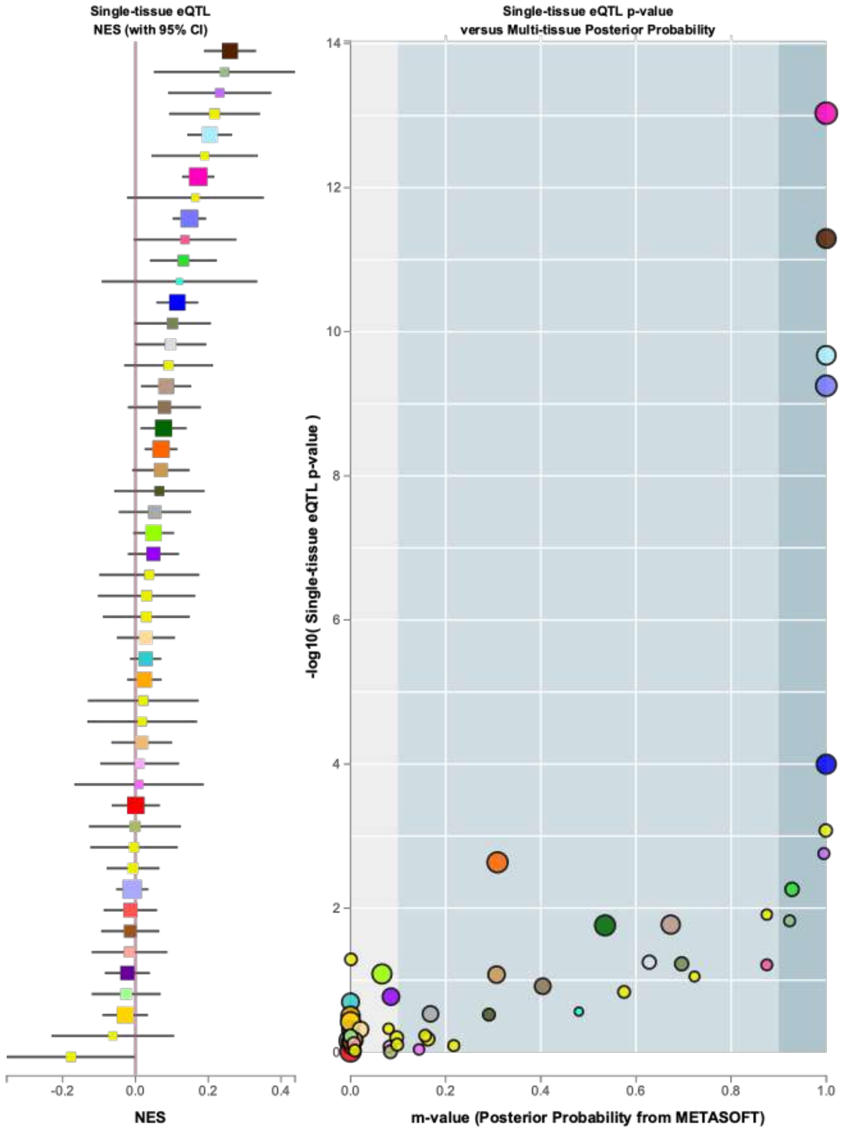

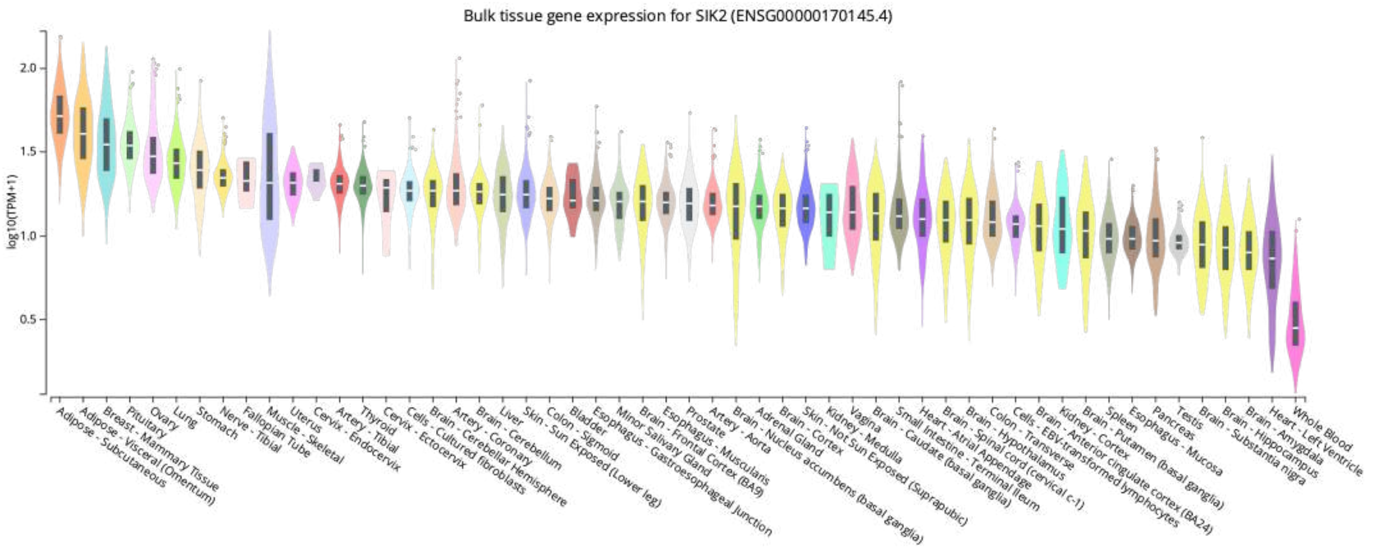

Exon Expression of SIK2: ENSG00000170145.4 salt inducible kinase 2 [Source:HGNC Symbol;Acc:HGNC:21680]

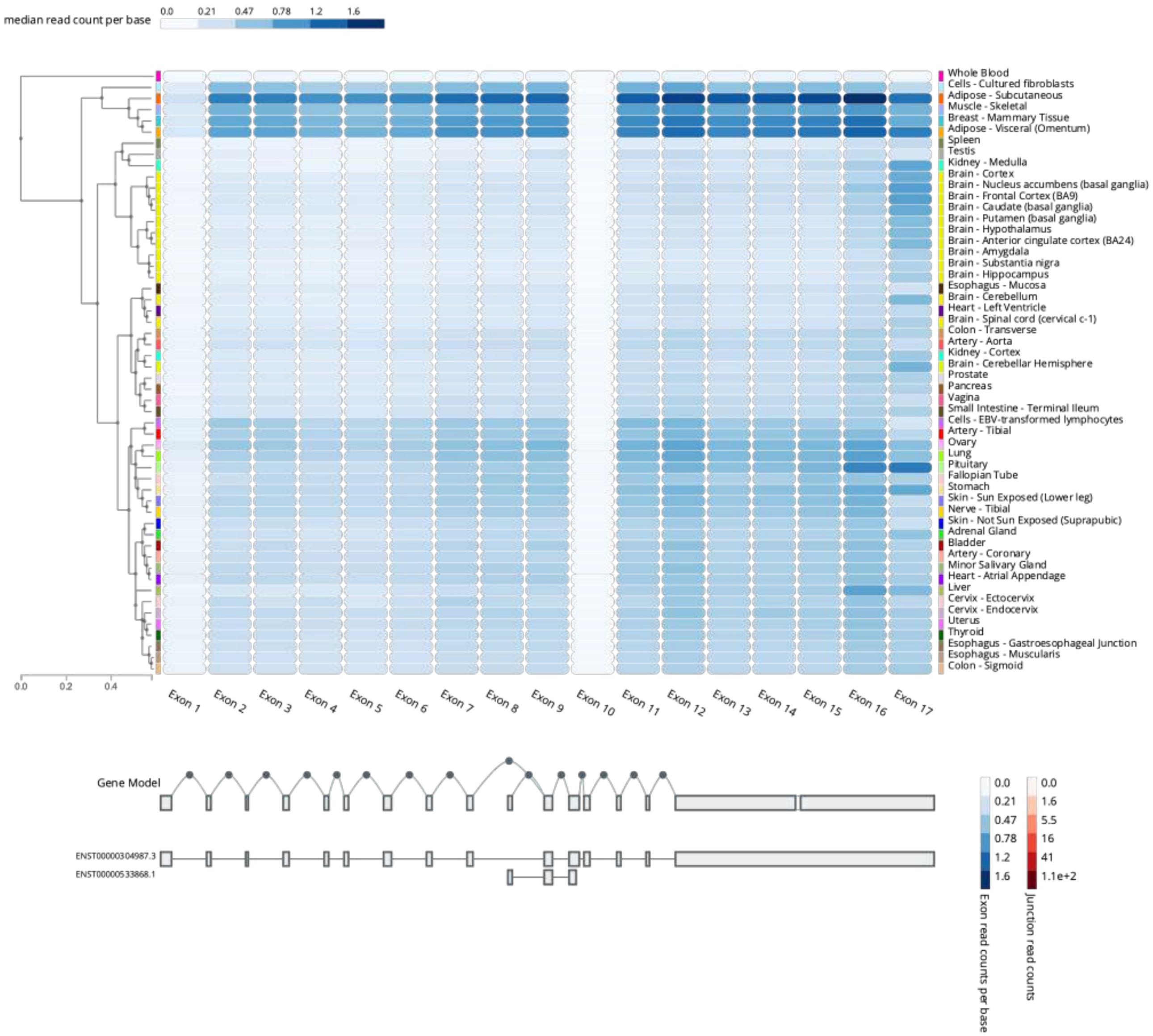

no significant eQTL

SH2B3 rs3184504

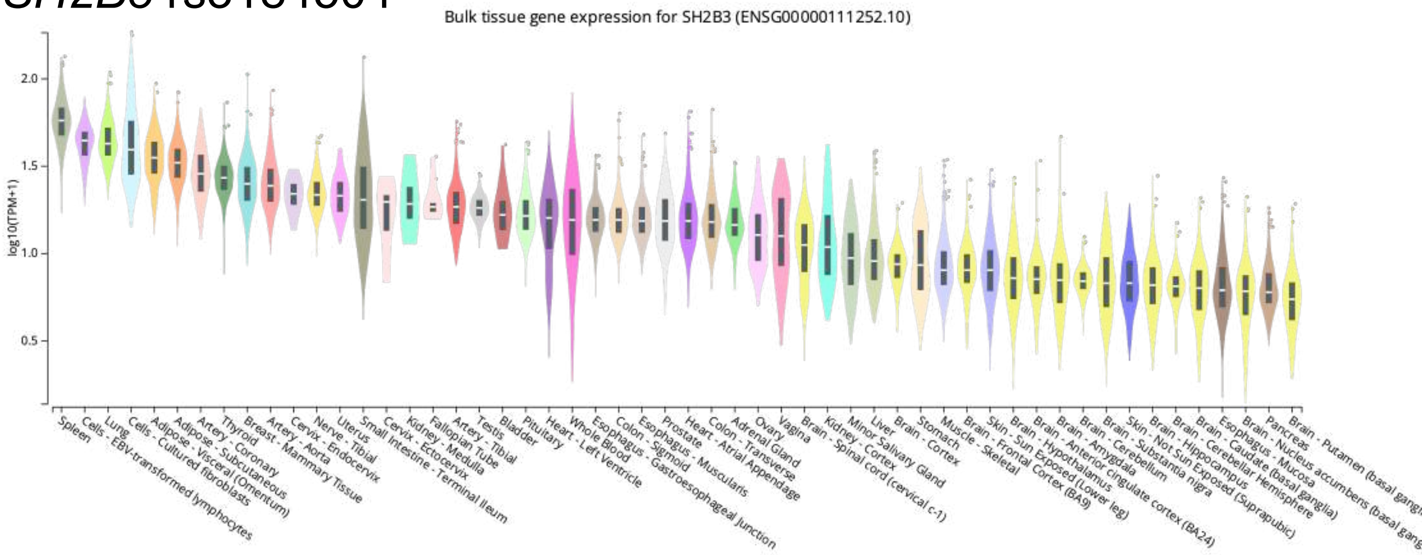

BACH2 rs56258221

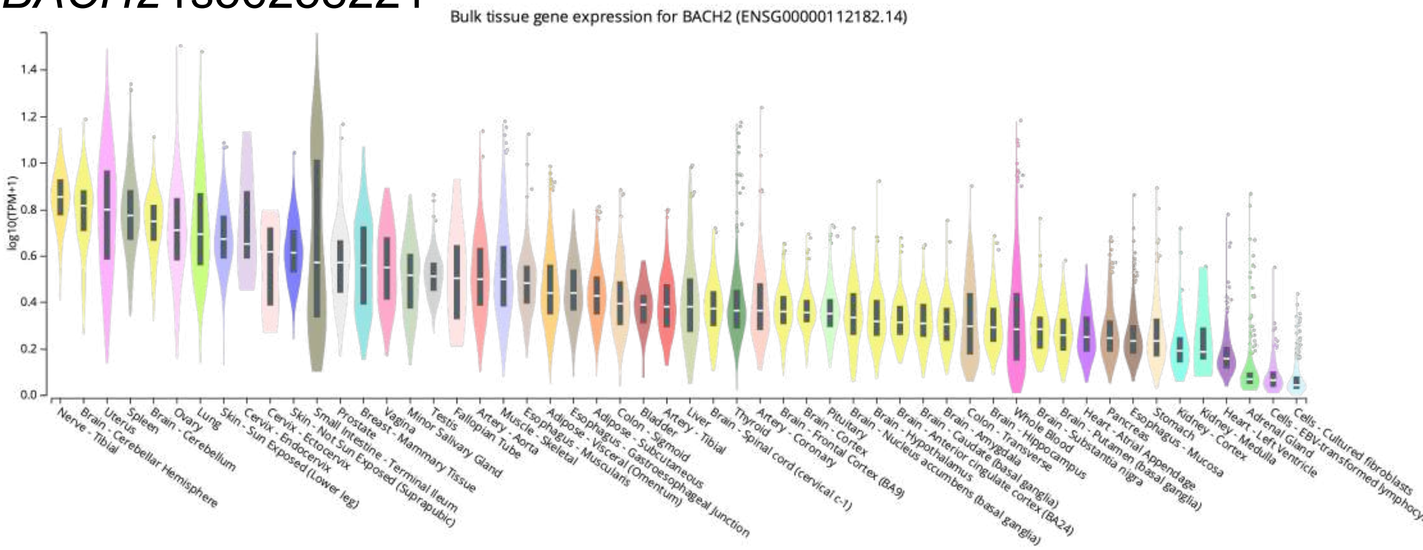

IL2RA rs4147359

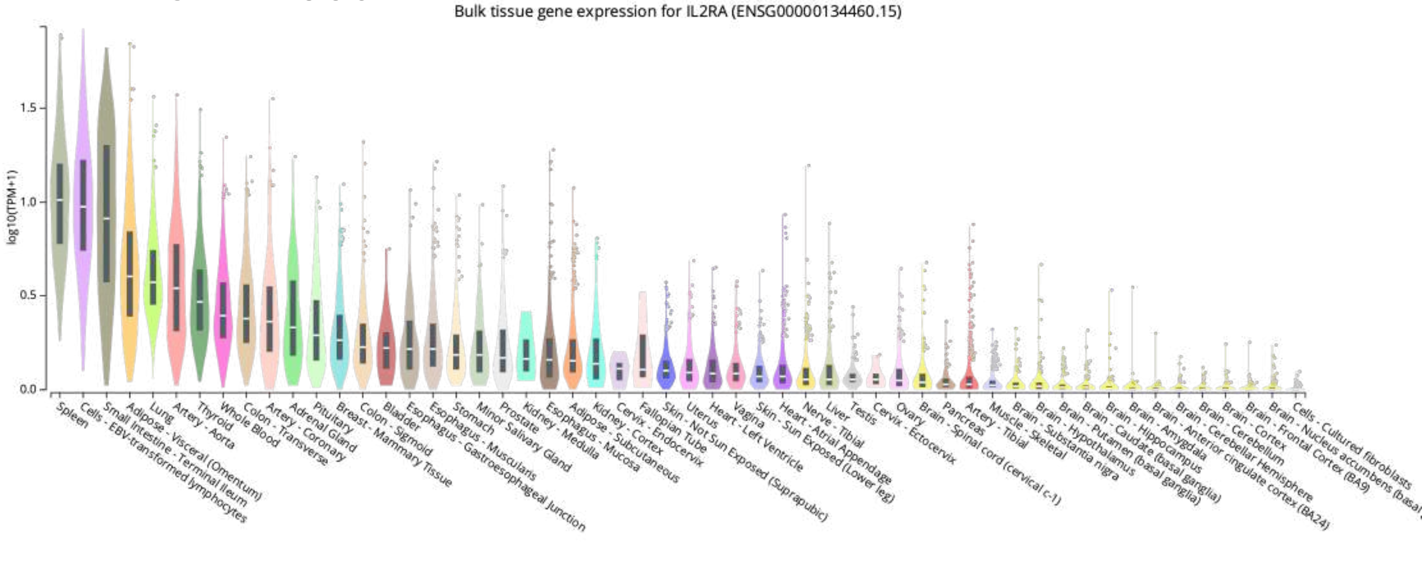

PRKD2 rs6065274

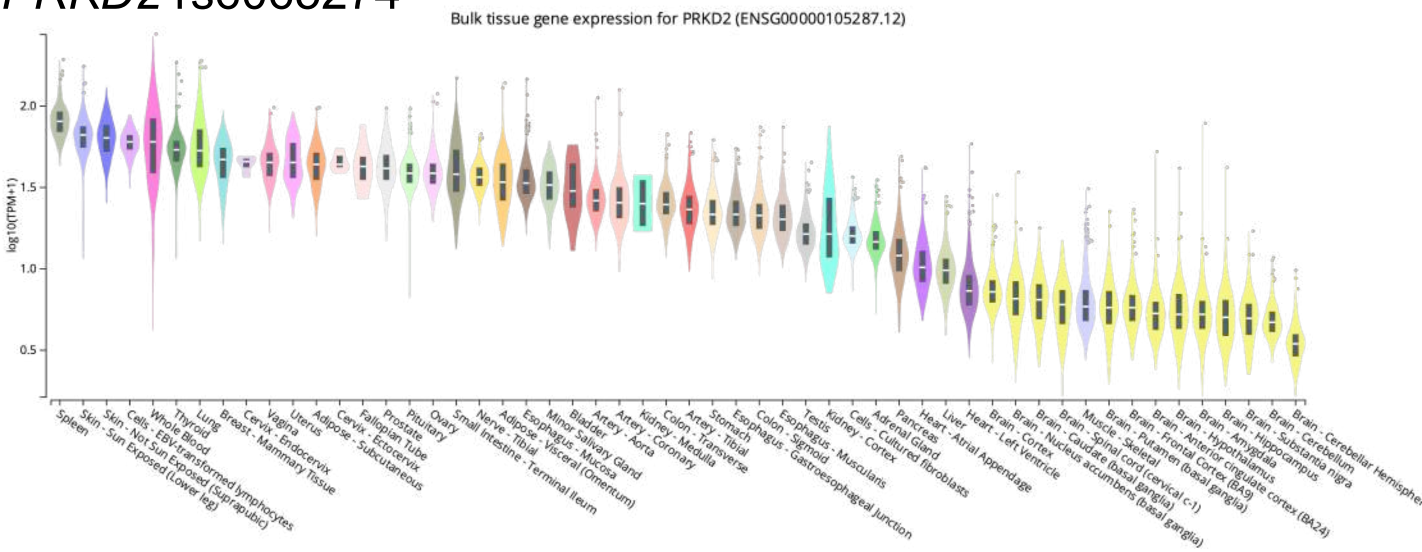

FOXP1 rs80060485

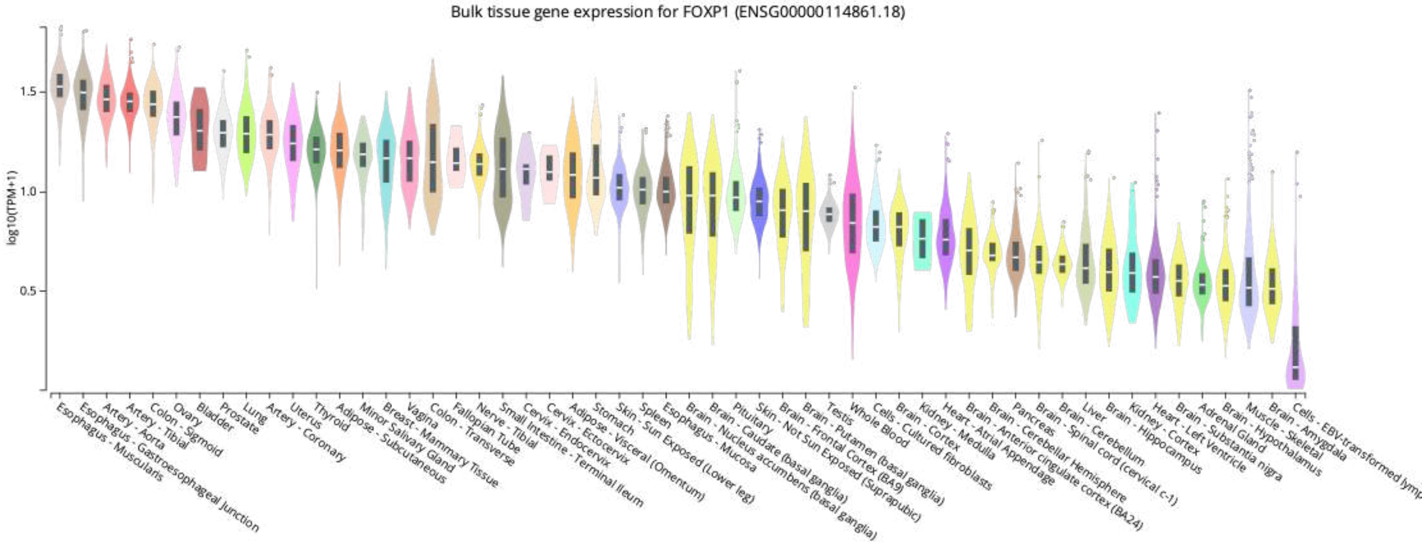

Supplement: Multimedia component 12 [file mmc12.pdf]
