# Supplementary material for: Proteome-scale autoantibody profiling in PSC: Associations with clinical phenotypes and evidence for neuroendocrine deregulations
Source: JHEP Rep. 2025 Dec 23;8(3):101719. doi: 10.1016/j.jhepr.2025.101719 (PMC12925457; doi:10.1016/j.jhepr.2025.101719)
Supplement: Multimedia component 13 [file mmc13.pdf]

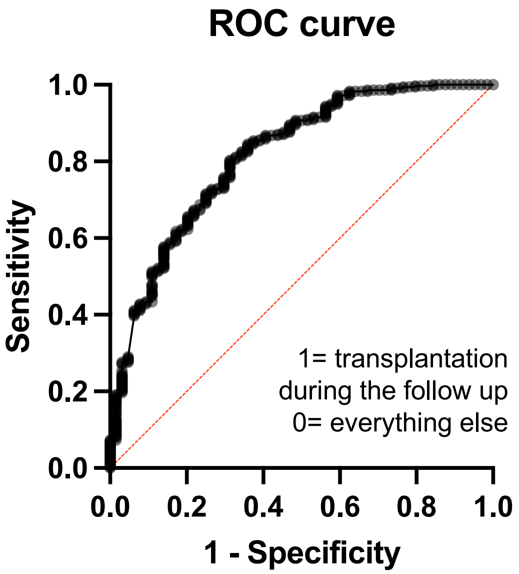

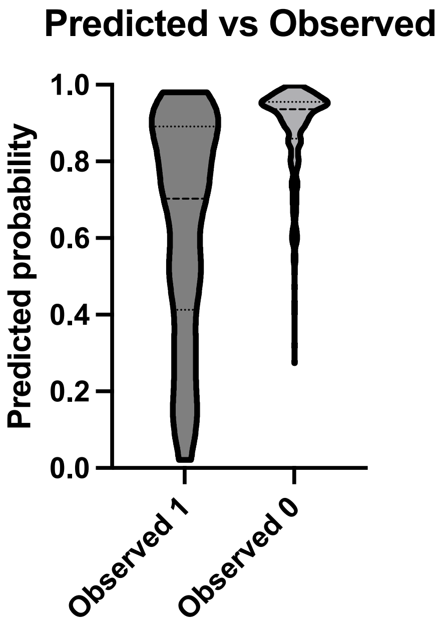

area under the ROC curve	
area	0.8153
std. error	0.03004
95% confidence interval	0.7564 to 0.8741
P-value	<0.0001

negative/positive predictive power	
negative predictive power (%)	80.77
positive predictive power (%)	89.06

predicted vs observed classifications

classification table	predicted 1	predicted 0	total	% correctly classified
observed 1	21	43	64	32,81
observed 0	5	350	355	98,59
total	26	393	419	88,54
